# Accomplishments and challenges in developing improved influenza vaccines: An evaluation of three years of progress toward the milestones of the influenza vaccines research and development roadmap

**DOI:** 10.1016/j.vaccine.2025.127431

**Published:** 2025-08-13

**Authors:** Julia T. Ostrowsky, Natalie C. Vestin, Angela J. Mehr, Angela K. Ulrich, Lauren Bigalke, Joseph S. Bresee, Martin H. Friede, Bruce G. Gellin, Keith P. Klugman, Usman N. Nakakana, Tian Yun Wang, Charlotte L. Weller, Michael T. Osterholm, Eve M. Lackritz, Kristine A. Moore, The Influenza Vaccines R and D Roadmap Taskforce, William Ampofo, Edward Belongia, Marco Cavaleri, Cheryl Cohen, Benjamin Cowling, Rebecca Jane Cox, Swati Gupta, Ian Gust, Scott E. Hensley, Irina Isakova-Sivak, Kari Johansen, Stacey Knobler, Florian Krammer, John C.W. Lim, John McCauley, Richard Pebody, Punnee Pitisuttithum, Rino Rappuoli, Tiago Rocca, Stacey Schultz-Cherry, Ethan C. Settembre, James Southern, Kanta Subbarao, John S. Tam, Rajeev Venkayya

**Affiliations:** a National Vaccine Institute and University of Ghana; Accra, Ghana; b Marshfield Clinic Research Institute, Marshfield, Wisconsin, USA; c European Medicines Agency, Amsterdam, The Netherlands; d National Institute for Communicable Diseases and University of the Witwatersrand, Johannesburg, South Africa; e University of Hong Kong, Hong Kong Special Administrative Region; f Department of Clinical Science, University of Bergen, Bergen, Norway; g International AIDS Vaccine Institute, New York City, NY, USA; h University of Melbourne, Melbourne, Australia; i University of Pennsylvania, Philadelphia, Pennsylvania, USA; j Institute of Experimental Medicine, St. Petersburg, Russia; k Public Health Agency of Sweden, Stockholm, Sweden; l Independent Public Health Consultant, Washington, DC, USA; m Icahn School of Medicine at Mount Sinai, New York City, NY, USA; n Ignaz Semmelweis Institute, Interuniversity Institute for Infection Research, Medical University of Vienna, Vienna, Austria; o Duke-National University of Singapore Medical School, Singapore; p Frances Crick Institute (retired), London, UK; q UK Health Security Agency, London, UK; r Mahidol University, Bangkok, Thailand; s Fondazione Biotecnopolo, Siena, Italy; t Instituto Butantan, São Paulo, Brazil; u St Jude Children’s Research Hospital, Memphis, Tennessee, USA; v Seqirus, Waltham, Massachusetts, USA; w Adviser to South African Health Products Regulatory Authority, Cape Town, South Africa; x Laval University, Quebec City, Quebec, Canada, and University of Melbourne, Doherty Institute, Melbourne, Australia; y Hong Kong Polytechnic University, Hong Kong Special Administrative Region; z Aerium Therapeutics, Foxboro, Massachusetts, USA; a Center for Infectious Disease Research and Policy, C315 Mayo Memorial Building, MMC 263, 420 Delaware Street, SE, Minneapolis, MN 55455. 612-626-6770, United States of America; b The Task Force for Global Health, Atlanta, GA, USA; c World Health Organization, Geneva, Switzerland; d Georgetown University (formerly with The Rockefeller Foundation during this project), Washington, DC, USA; e The Gates Foundation, Seattle, Washington, USA; f Wellcome Trust, London, United Kingdom

**Keywords:** Influenza, Pandemic preparedness, Seasonal influenza vaccines, Universal influenza vaccines, Broadly protective influenza vaccines

## Abstract

Influenza vaccines that provide more effective immunity to seasonal influenza as well as protection against a broad range of emerging influenza viruses with pandemic potential are needed to reduce the public-health burden of influenza and enhance pandemic preparedness. The Influenza Vaccines Research and Development (R&D) Roadmap (IVR) was published in 2021 to serve as a strategic planning tool to advance influenza vaccine R&D. Following IVR publication, a 3-year monitoring, evaluation, and adjustment (ME&A) program was implemented to assess progress in meeting the milestones outlined in the IVR. As of mid-May 2025, 16 (17%) of the 93 milestones had been accomplished or partially accomplished, with the majority (67; 72%) in various stages of progress. Of the 35 milestones designated high-priority, five (14%) had been accomplished or partially accomplished, 29 (83%) are in progress, and no progress was identified for one (3%). Key accomplishments include: establishing longitudinal cohort studies to characterize immune responses to influenza virus infection and vaccination by age over time and by vaccine product; creating a comprehensive landscape of innovative influenza vaccine technologies in preclinical and clinical development; advancing next-generation and broadly protective influenza vaccine candidates into clinical trials; identifying relevant lessons learned from accelerated SARS-CoV-2 vaccine development during the COVID-19 pandemic; and initiating development of a full value of improved influenza vaccine assessment (FVIVA) to inform investment and guide the eventual uptake of improved vaccines globally. Persistent challenges include clarifying immune mechanisms for generating durable and broadly protective immunity, enhancing understanding of immune imprinting and the role of mucosal immunity in preventing infection and transmission, identifying correlates of protection, and exploring regulatory options for broadly protective influenza vaccine licensure. The IVR ME&A program provides a basis for ongoing critical review of progress in influenza vaccine R&D to inform decision-making on research priorities and funding.

## Highlights

• Improved seasonal influenza vaccines can reduce the global burden of influenza.
• Broadly protective flu vaccines will enhance preparedness for pandemic influenza.
• Many next-generation influenza vaccine candidates are in the R&D pipeline.
• Continued research is critical to resolve key issues in immunology and vaccinology.
• Consensus is needed to determine the basis for licensing broadly protective vaccines.

## 1 Introduction

The dramatic spread of avian influenza A (H5N1) viruses in poultry, dairy cows, and other mammals around the globe [1,2], as well as sporadic H5N1, H7N9, and H9N2 infections in humans, highlight the potential for rapid adaptation of influenza viruses and the possibility for a new pandemic influenza strain to emerge [3]. Current challenges in responding to novel viruses include the lag time required to develop a new strain-specific vaccine, limited capabilities for rapid scale-up of vaccine production, and the potential for extreme inequities in vaccine distribution as seen during the coronavirus 2019 (COVID-19) pandemic. A recent study in a nonhuman primate model demonstrated that pre-exposure prophylaxis with a broadly neutralizing antibody was effective in protecting against severe disease caused by a highly pathogenic strain of H5N1, supporting the utility of developing broadly protective vaccines for pandemic preparedness [4]. Availability of broadly protective influenza vaccines at the outset of a pandemic would provide immediate and ongoing measures for protection as new variants emerge, thereby saving countless lives and reducing socioeconomic and healthcare-system impacts [5]. Furthermore, broadly protective and durable vaccines that protect against a wider range of influenza viruses would benefit seasonal influenza vaccination programs, potentially obviating the need for annual revaccinations, reducing costs of immunization, expanding global vaccine demand, and improving vaccine equity and access.

In addition to concerns about pandemic preparedness, the current strain-specific approach to seasonal influenza vaccine formulation and vaccination has significant limitations. Chief among these are suboptimal vaccine effectiveness and the potential for antigenic mismatches between selection of geographically appropriate virus strains for vaccine production and circulating wild-type influenza strains. These factors, combined with the need for annual revaccinations, contribute to vaccine underuse and barriers to seasonal influenza vaccination programs in low- and middle-income countries (LMICs) [6,7]. Despite availability of seasonal influenza vaccines, the annual global influenza disease burden remains high, with an estimated 290,000 to 645,000 influenza-related deaths and over 5 million hospitalizations each year [8,9]. Thus, even incremental improvements in current seasonal influenza vaccines could substantially reduce global influenza morbidity and mortality and secondary societal and economic impacts.

In response to these needs, the Global Funders Consortium for Universal Influenza Vaccine Development (GFC), within the Task Force for Global Health, was established in 2017 to accelerate the development and availability of broadly protective and durable influenza vaccines [10]. One of the GFC's primary recommendations was to generate a research and development (R&D) roadmap for broadly protective influenza vaccines, given that the challenges of improving vaccines for a respiratory virus such as influenza virus are substantial, highly complex, interdisciplinary, and require a global perspective to meet the needs of diverse populations. The Influenza Vaccines R&D Roadmap (IVR) project was initiated in 2019 and published in 2021 to serve as a strategic planning tool to promote international stakeholder engagement, coordinate funding, and facilitate progress in influenza vaccine R&D [11].

**Fig. 1:**
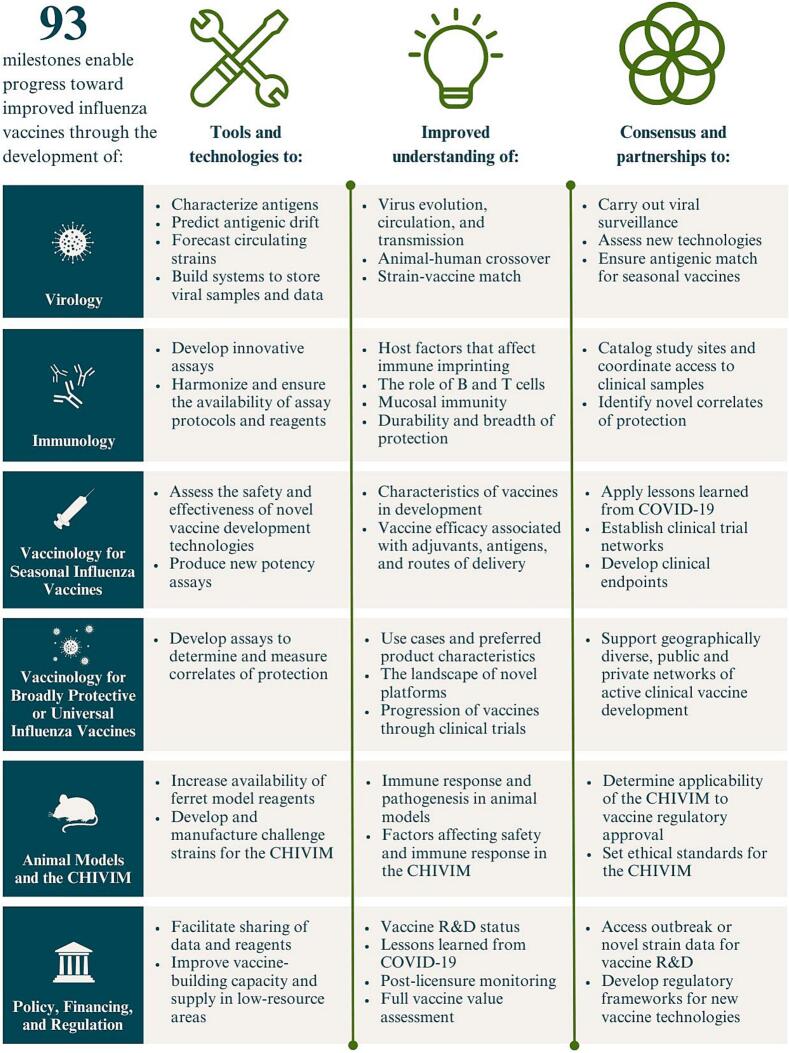
Conceptual overview of research activities by IVR topic area. Description: This figure illustrates the tools and technologies needed for each IVR topic area that will improve understanding of key concepts within those topic areas. The figure also describes the necessary areas where partnerships or consensus are needed within each of the topic areas.

Following a consensus-driven process that involved more than 100 subject-matter experts from 29 countries, the Center for Infectious Disease Research and Policy (CIDRAP) at the University of Minnesota published the IVR in 2021 [11]. The IVR highlights opportunities and challenges across six focus areas: virology; immunology; vaccinology for seasonal vaccines; vaccinology for broadly protective or universal vaccines; animal models and controlled human influenza virus infection models (CHIVIMs); and policy, financing, and regulation (Fig. 1). Each area contains strategic goals that cover broad concepts, and a set of more specific milestones that are measurable. Since initial publication of the IVR, the goals and milestones have been reviewed and updated annually with critical feedback from the IVR taskforce, a diverse group of experts from academia, industry, government, and nongovernmental organizations [12].

In addition to the roadmap, the IVR Initiative encompasses four complementary, interrelated components: a 3-year monitoring, evaluation, and adjustment (ME&A) program to assess progress in meeting the IVR goals and milestones; the Universal Influenza Vaccine Technology Landscape to compile publicly available information on universal, broadly protective, and next-generation influenza vaccines in development; the IVR Funding Tracker to monitor global investment in influenza vaccine R&D; and stakeholder outreach activities [12].

Given that the IVR provides a comprehensive framework for generating improved influenza vaccines, the purpose of this report is to summarize progress over the last 3 years toward meeting the IVR milestones as a way of capturing important progress and remaining challenges in influenza vaccine R&D.

## 2 Methods

Monitoring and evaluating progress toward the IVR goals and milestones involved four activities: literature review, peer review/expert input, monitoring candidate influenza vaccines in the development pipeline, and data compilation. Methods for each are detailed below.

### 2.1 Literature review

The CIDRAP team collected information relevant to R&D progress from a wide range of available English-language sources published between September 2021 and mid-May 2025 and organized them into an online reference manager database. Primary sources of information were Google Scholar and PubMed, using the following key search terms: universal, broadly protective, and next-generation influenza vaccines. Additional search terms included seasonal influenza, pandemic influenza, immunogenicity, mucosal immunity, animal models, controlled human infection models, correlates of protection, safety, efficacy, and regulatory*.* We also routinely reviewed publications from the Collaborative Influenza Vaccine Innovation Centers (CIVICs) and the Centers of Excellence in Influenza Research and Response (CEIRR), which are sponsored by the 10.13039/501100004477US 10.13039/100000060National Institute of Allergy and Infectious Diseases (NIAID), 10.13039/100000002National Institutes of Health (NIH). Additionally, we obtained information from preprint servers, such as medRxiv and bioRxiv; clinical trial registries, such as clinicaltrials.gov, EudraCT, and the International Clinical Trials Registry Platform; research funding databases (Europe PMC, NIH RePORT); and grey literature sources, such as conference proceedings, government documents, pharmaceutical company websites, industry news sources, and organizational reports (e.g., from the World Health Organization [WHO] and the Sabin Vaccine Institute).

### 2.2 Peer review/expert input

At the outset of the IVR Initiative, a steering group and an expert taskforce were formed to support development of the roadmap [11]. After the IVR was published, CIDRAP expanded these groups to support identification of progress on the IVR milestones. The CIDRAP team convened the steering group quarterly and the taskforce annually and worked with the GFC Industry Engagement Working Group to obtain information on industry perspectives and activities relevant to IVR milestones.

### 2.3 Monitoring candidate influenza vaccines in the development pipeline

The IVR ME&A program tracked vaccine development through the Universal Influenza Vaccine Technology Landscape [[13], [14], [15]], which compiles information on universal, broadly protective, and next-generation influenza vaccine candidates in preclinical and clinical development. The Landscape encompasses a range of vaccine technologies designed to provide broader and more durable immunity against circulating seasonal and pandemic influenza viruses, compared with current conventional strain-specific seasonal influenza vaccines. As of mid-May 2025, the Landscape database included 228 vaccine candidates based on six different platforms, including influenza viruses (24), nucleic acids (47), recombinant proteins (44), virus vectors (29), virus-like particles (VLPs) (24), and non-VLP nanoparticles (60). Of these, 186 are in preclinical development and 42 have reached clinical evaluation, with 26 in phase 2 or 3 clinical trials. The database is publicly accessible via the IVR website and is continuously updated as new information becomes available.

### 2.4 Data compilation

CIDRAP created a database that compiled information for each of the IVR goals and milestones. A milestone was designated high priority if taskforce members agreed that completing the milestone was critical for improving seasonal influenza virus vaccines or generating universal or broadly protective vaccines. Information from the sources identified above was incorporated into the database continuously, unless specified otherwise, and summarized annually for review by the IVR steering group and taskforce. Each milestone within the six areas of focus was categorized as “fully or partially accomplished” (with partially meaning that one or more aspects of the milestone were completed), “in progress,” or “no progress.” Milestones were categorized as “in progress” if the team identified available literature or ongoing research activities that supported moving the milestone toward completion. Some milestones are expected to remain “in progress” for a number of years, owing to the complexity of the research and the need for long-term research support. CIDRAP produced annual IVR ME&A progress reports in 2022 and 2023, which are posted on the IVR website, and presented updates each year to the GFC at their annual meeting. Ongoing engagement and recommendations from the IVR taskforce led to revisions, consolidation, or elimination of certain goals and milestones, accounting for the differences in total number of milestones in this report compared to the original IVR publication.

## 3 Results

**Fig. 2:**
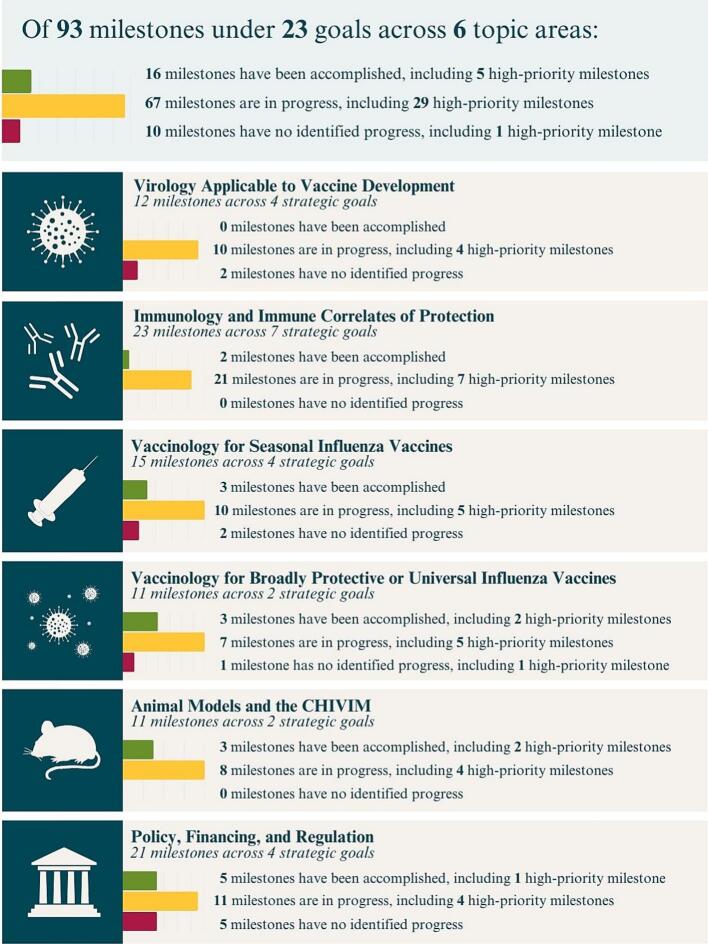
Progress on IVR milestones by topic area, May 2025. Description: This figure graphically shows progress by topic area for the 93 IVR milestones. Progress is organized into 3 categories: Accomplished, In Progress, or No Progress.

The IVR includes 23 broad strategic goals (Table 1) and 93 milestones across the six areas of focus, with 35 milestones considered high priority (Table 2). As of mid-May 2025, 16 (17%) of the 93 milestones had been accomplished (fully or partially), and the majority of all milestones were in various stages of progress (Fig. 2). All but one of the 35 milestones designated high priority have been accomplished or are in progress (Table 3). Detailed information on milestone progress is provided in Appendix A (supplemental material) along with additional supporting documentation. Highlights of important progress and persistent challenges for each topic area are provided below.

**Table 1:** Strategic goals identified in the IVR, May 2025.

Topic 1: Virology Applicable to Vaccine Development	
1.1	Improve understanding of human and animal influenza virus evolution.
1.2	Enhance the ability to forecast viruses that are likely to circulate in the upcoming season to improve the antigenic match between circulating influenza viruses and viral strains selected for vaccine production.
1.3	Improve the ability to detect and understand the emergence of novel influenza viruses with pandemic potential.
1.4	Enhance understanding of factors associated with viral transmissibility.
Topic 2: Immunology and Immune Correlates of Protection	
2.1	Promote the development and standardization of immunologic tools to inform the development of universal, broadly protective, and next-generation influenza vaccines.
2.2	Gain better understanding of human immunology to inform influenza vaccine development through research focused on new tools and technologies.
2.3	Improve understanding of aspects of the B-cell immune response to influenza infection and vaccination that are important for developing better vaccines and optimal strategies for vaccination, particularly in the context of partial preexisting immunity from continual exposure to influenza viruses.
2.4	Determine the impact of prior influenza virus infection or vaccination on future immune responses to influenza viruses or vaccines.
2.5	Clarify the role of T cells in generating or supporting protective immunity to influenza virus infection and/or vaccination.
2.6	Improve understanding of the role of mucosal immunity in protecting against influenza.
2.7	Develop novel correlates of protection for assessing seasonal influenza vaccines and broadly protective or universal influenza vaccines, as part of clinical studies that demonstrate efficacy against a disease endpoint.
Topic 3: Vaccinology for Seasonal Influenza Vaccines	
3.1	Promote strategies that shorten the lag time from identification of candidate vaccine viruses through the process of annual vaccine production and release.
3.2	Identify strategies and policies to optimize seasonal influenza vaccines and improve vaccine benefit-risk profiles.
3.3	Further assess the role of existing and new adjuvants in creating next-generation seasonal influenza vaccines.
3.4	Determine the role of NA as a vaccine antigen for improving the effectiveness and immunogenicity of seasonal influenza vaccines.
Topic 4: Vaccinology for Broadly Protective or Universal Influenza Vaccines	
4.1	Identify the most promising broadly protective or universal influenza vaccine candidates that elicit durable protection against influenza viruses in preclinical studies, with a focus on targeting conserved regions of the virus.
4.2	Evaluate the most promising broadly protective or universal influenza vaccine candidates, using at least several different platforms, in clinical trials.
Topic 5: Animal Models and the Controlled Human Influenza Virus Infection Model (CHIVIM)	
5.1	Optimize animal models for influenza vaccine research.
5.2	Address steps needed to further develop and refine the CHIVIM.
Topic 6: Policy, Financing, and Regulation	
6.1	Catalyze broad support and sustained funding for developing improved seasonal influenza vaccines and broadly protective or universal influenza vaccines.
6.2	Promote innovation for developing improved seasonal influenza vaccines and broadly protective or universal influenza vaccines.
6.3	Promote information sharing aimed at moving influenza vaccine development forward.
6.4	Address regulatory challenges in the evaluation and licensure of next-generation, broadly protective, and universal influenza vaccines.

**Table 2:** High-priority milestones by topic area, May 2025.

**Topic Area**	**Milestones**
Virology Applicable to Vaccine Development	• Milestone 1.1.a: Refine strategies to improve efficiencies in obtaining sequences and isolates of circulating influenza viruses in human populations and a broad range of animal species, including species not previously recognized as important for influenza, such as bovine. • Milestone 1.1.d: Develop a comprehensive landscape of population-based serosurveillance studies in different age groups and geographic regions to better understand the relationship between population immunity to seasonal influenza viruses and antigenic drift, which is needed to determine how serosurveillance data can be used to improve decisions regarding vaccine strain selection for seasonal influenza vaccine production. • Milestone 1.2.c: Continue to develop, harmonize, and implement methods (e.g., the use of predictive artificial intelligence and other new technologies) to inform antigenic characterization of H1N1 and H3N2 viruses. • Milestone 1.4.a: Review optimal study designs for evaluating influenza transmission, including controlled human influenza virus infection model (CHIVIM) studies and household and school-based transmission studies.
Immunology and Immune Correlates of Protection	• Milestone 2.2.b: Determine key mechanisms of durability of protective immunity following influenza virus infection, including the discovery of early biomarkers associated with durable immune responses. • Milestone 2.4.b: Determine through birth-year cohort or clinical studies how repeated influenza vaccinations affect immune responses to subsequent influenza vaccinations, including immune responses to HA, NA, and other antigens. • Milestone 2.4.c: Determine how the initial encounter with an influenza virus or vaccine (i.e., immune imprinting) affects B- and T-cell responses, including immunologic responses to subsequent influenza virus infection and/or vaccination. • Milestone 2.6.a: Characterize the role of mucosal immunity (including antibodies and T cells) in protecting against influenza virus infection, disease, and transmission. • Milestone 2.7.b: Develop correlates of protection for mucosal immunity based on different vaccine platforms (e.g., live-attenuated influenza virus) and routes of administration (e.g., intranasal). • Milestone 2.7.c: Conduct side-by-side standardized comparative studies of immune markers, beyond serum HAI titers, as potential correlates of protection in vaccine efficacy/effectiveness studies or in studies that use a controlled human influenza virus infection model. • Milestone 2.7.d: Develop correlates of protection for influenza vaccine candidates based on antigens other than HA (e.g., NA, NP, HA stem), for vaccines produced using novel platforms (e.g., nanoparticles and nucleic acids), and relevant to a variety of endpoints (e.g., cellular immune responses, severe disease).
Vaccinology for Seasonal Influenza Vaccines	• Milestone 3.2.b: Review the development and safety of novel vaccine platforms to identify how best to apply them to develop improved seasonal influenza vaccines. • Milestone 3.2.f: Evaluate the immunogenicity and efficacy of alternate routes of vaccine delivery (e.g., intranasal, oral, intradermal needle-free) to enhance mucosal immunity and/or block transmission. • Milestone 3.3.a: Determine, through clinical studies, if any promising adjuvant candidates under investigation can substantially improve vaccine efficacy in the elderly and assess their safety profiles. • Milestone 3.3.b: Determine, through clinical studies, if adjuvants substantially improve vaccine efficacy in the very young and assess their safety profiles. • Milestone 3.4.d: Determine if the presence of NA improves new or next-generation seasonal influenza vaccines, and, if so, establish the optimal dose of NA that improves immunogenicity and effectiveness and maintains an acceptable safety profile.
Vaccinology for Broadly Protective or Universal Influenza Vaccines	• Milestone 4.1.c: Continue research on new antigen design to enable the development of broadly protective or universal influenza vaccines. • Milestone 4.1.d: Convene a workshop to review the scientific challenges and recent advancements in development of novel vaccine platforms to determine how best to guide R&D for broadly protective influenza vaccines for regulatory approval, global scale-up, and rapid deployment in response to pandemic influenza viruses. • Milestone 4.1.e: Define selection criteria (e.g., including immunogenicity data from pre-immune animal models and considerations for global manufacturing capacity) for advancing preclinical influenza vaccine candidates into clinical evaluation. • Milestone 4.2.b: Define and prioritize clinical endpoints for evaluating the efficacy of broadly protective influenza vaccines (e.g., prevention of laboratory-confirmed influenza, severe complications of influenza, hospitalization, death) to compare outcomes across studies. • Milestone 4.2.c: Identify an initial set of vaccine candidates that demonstrate broad-based immunity—humoral, cell-mediated, or both—in preclinical research and assess them for safety and immunogenicity in phase 1 clinical trials in healthy adults. • Milestone 4.2.d: Determine biomarkers correlated with immune protection for different vaccine platforms. • Milestone 4.2.e: Continue to analyze outcomes from broadly protective vaccine candidates in phase 1 trials and advance promising candidates into phase 2 and phase 3 clinical trials, including in high-risk populations. • Milestone 4.2.f: Identify the most promising vaccine candidates from phase 2 trials and facilitate good manufacturing practice (GMP) and evaluation in phase 3 trials, such as through sponsorship in public-private partnerships.
Animal Models and the Controlled Human Influenza Infection Model (CHIVIM)	• Milestone 5.1.b: Sustain funding for validated reagents, updated viral stocks, and harmonized assays, which are needed to evaluate innate and adaptive immune responses in ferrets and other animals such as hamsters and to facilitate comparison of studies across laboratories. • Milestone 5.1.e: Develop comparative vaccine studies using the high-risk ferret model to inform predictions of vaccine responses in high-risk human populations. • Milestone 5.1.f: Review a comprehensive analysis of the predictive value of different animal models, including natural hosts such as pigs and horses, for influenza vaccine studies (for both seasonal and broadly protective vaccines). • Milestone 5.1.g: Develop and validate animal models for evaluating immune responses—including durability and mucosal immunity—to broadly protective influenza vaccines. • Milestone 5.2.a: Generate guidance, including ethical and safety considerations, for using the CHIVIM. • Milestone 5.2.b: Ensure the broad availability to investigators of a biorepository of diverse, well-characterized challenge viruses, cell lines, and human samples for manufacture and CHIVIM studies.
Policy, Financing, and Regulation	• Milestone 6.1.a: Develop and disseminate a full value of vaccine assessment (FVVA; also referred to as the full value of influenza vaccine assessment [FVIVA]) for improved seasonal and broadly protective, universal influenza vaccines that addresses different vaccine use cases and includes an assessment for low- and middle-income countries (LMICs). • Milestone 6.1.b: Develop targeted and creative communications and advocacy strategies and necessary communication tools that build on the FVIVA and provide information on economic costs, the risk of future influenza pandemics, and the need for investment in influenza vaccine R&D. • Milestone 6.2.a: Distill lessons learned for influenza vaccines from experience with COVID-19 vaccine R&D, including clinical research and study designs, manufacturing, distribution, advocacy, financing, and global collaboration. • Milestone 6.3.b: Assess and document the ongoing impact of the Nagoya Protocol and possibly related national Access and Benefit Sharing (ABS) legislation, on sharing of influenza isolates and gene sequences in relation to influenza vaccine R&D; share findings with key policymakers (e.g., ministries of health/agriculture and global trade); and determine strategies to address potential unintended consequences. • Milestone 6.4.a: Convene a workshop to address regulatory science issues regarding the evaluation of next-generation and broadly protective influenza vaccines for pandemic preparedness and seasonal influenza vaccination and publish a consensus summary report.

**Table 3:** Summary of progress toward meeting high-priority IVR milestones by topic area, May 2025.

Topic Area	No.	Accomplished	In Progress	No Progress Identified
Virology Applicable to Vaccine Development	4	0	4	0
Immunology and Immune Correlates of Protection	7	0	7	0
Vaccinology for Seasonal Influenza Vaccines	5	0	5	0
Vaccinology for Broadly Protective or Universal Influenza Vaccines	8	2	5	1
Animal Models and the Controlled Human Influenza Virus Infection Model (CHIVIM)	6	2	4	0
Policy, Financing, and Regulation	5	1	4	0
**TOTAL**	**35**	**5 (14%)**	**29 (83%)**	**1 (3%)**

## 4 Virology

### 4.1 Key progress

The virology area focuses on improving antigenic characterization of influenza viruses for seasonal influenza vaccine production and assessing transmissibility of influenza viruses. Improving the selection of vaccine seed strains depends in part on strengthening the ability to forecast viruses likely to be circulating in the upcoming season and to characterize the antigenicity of the predominant circulating strains of H1N1, H3N2, and influenza B viruses. Improving the ability to forecast antigenic changes in influenza viruses will require monitoring mutation occurrence in circulating strains and antigenic changes affecting the dynamics of influenza epidemics, primarily through the use of machine learning tools [16]. To make substantial progress in understanding population-based immunity and factors affecting antigenic drift, mapping of human immune response landscapes through serosurveillance studies is essential. Greater availability, precision, and diversity of antigen data will support the goal of predicting upcoming dominant strains [17,18], identifying hemagglutinin (HA) and neuraminidase (NA) evolution drivers and their effects on epidemic trajectories [19], and identifying mutations that can lead to antibody escape [20].

A high-priority milestone in this area focuses on developing, standardizing, and implementing methods to improve antigenic characterization of H1N1 and H3N2 viruses. Recent progress to this end includes identifying mechanisms that underpin drift using a modified Bayesian model of genetic and antigenic data [21], the development of a high-throughput protocol to sequence the H3N2 genome directly from respiratory samples [22], and an attribute network embedding technique to predict antigenic distances between H3N2 strains [23]. Several recent studies have also advanced the development of computational methods to improve evolutionary forecasting of H3N2 [[24], [25], [26]].

### 4.2 Persistent challenges

Several milestones in this area focus on the use of machine-learning models and phylogenetic analysis. Optimizing these tools requires further efforts to build consensus for standardization and appropriate use of deep-sequencing technology and systems biology to improve antigenic characterization of influenza viruses, identify new epitopes, determine the frequency with which animal strains infect humans, and ultimately predict emergence of novel viruses. Additionally, three high-priority milestones emphasize the consistently unmet need to expand global surveillance of influenza viruses and the availability of sequences and isolates of circulating viruses in humans and a broad range of animal species to improve detection of novel viruses with pandemic potential.

## 5 Immunology

### 5.1 Key progress

The immunology area highlights several key areas of research, such as enhancing understanding of B- and T-cell responses to influenza infection, clarifying how immune imprinting affects subsequent response to viral exposure or vaccination, defining the role of mucosal immunity in protecting against infection and transmission, and providing additional assessment tools for influenza vaccine R&D, such as longitudinal cohort studies, reagents, and assays. Identifying new correlates of protection is critical for evaluating the efficacy of broadly protective influenza vaccines, which may involve novel vaccine platforms, mucosal routes of administration, and antigen targets other than the HA head [27,28]. T-cell correlates of protection have been identified for an oral virus-vectored next-generation influenza vaccine candidate [29,30], and researchers associated with the FLUCOP consortium on standardization and development of assays for influenza vaccine correlates of protection have advanced understanding of immune-marker correlates of protection by comparing serology titers from harmonized HA inhibition (HAI) and microneutralization (MN) assays [31].

Two strategic goals focus on improving understanding of B- and T-cell responses to influenza virus infection and vaccination. Recent research has evaluated factors that trigger and control memory B-cell responses—with an emphasis on those that contribute to breadth and durability of immunity—in the lungs, lymph nodes, and other germinal centers, following infection and vaccination [32,33]. Additional work has supported defining mechanisms that contribute to post-immunization effector B-cell antibody production [34] and clarifying pathways that contribute to protective immunity by vaccine-elicited IgG antibodies that target the more conserved HA stem region [[35], [36], [37]]. Another study showed that co-immunization with HA stem immunogens of group 1 and group 2 influenza A viruses elicited broadly cross-protective antibodies in animals, which may offer a strategy for generating broadly protective vaccines [38]. Progress in understanding the presence and evolution of broadly protective and long-lasting antibodies produced by plasma cells has involved assessments of the role of antigen-secreting plasma cells in the development of durable humoral immunity and the maintenance of serum antibody titers [39,40]. Recent animal and human studies of T-cell responses to influenza infection or vaccination have examined a range of topics applicable to influenza vaccines, including intranasal CD4 T-cell elicitation and dose- and preexisting antibody-dependent CD8 T-cell activation [41], associations between specialized T-cell effectors and age [42,43], and the function and activation of CD8 and memory T cells in the lungs [44].

Several milestones aim to improve understanding of immune imprinting, acquired immune responses, and mechanisms of innate immunity that can inform the breadth and durability (i.e., 3 to 5 years or more) of protective immunity from improved influenza vaccines. Two of these milestones have been accomplished: 1) the periodic convening of an international workshop to identify advances in understanding immune responses to influenza infection and vaccination [45] and 2) the establishment of longitudinal clinical studies to characterize immune responses to infection and vaccination in age-group cohorts in different geographical locations [[46], [47], [48]]. Recent studies have also evaluated age-specific immune responses, immune priming, and antibody seroprevalence and durability following influenza virus infection or vaccination [42,[49], [50], [51]]; lineage-specific and cross-reactive immune imprinting [52,53]; the effects of repeated vaccination and accumulated immunity [54,55]; and broadly protective antibodies associated with antigenic humoral signatures [56].

### 5.2 Persistent challenges

Many of the milestones related to immunology represent fundamental research questions that remain unanswered and require additional understanding before generating practical outcomes for influenza vaccine development, such as navigating the effects of preexisting immunity and determining mechanisms for inducing broadly protective and durable B-cell memory responses. Accordingly, all except two of the 23 milestones (including all seven high-priority milestones) in the immunology area were identified as “in progress.” Ongoing research is needed to identify correlates of protection for evaluating broadly protective influenza vaccines, clarify mechanisms for generating durable and broadly protective immunity, and continue longitudinal studies that will provide data on immune imprinting and impacts of prior influenza vaccination on immune responses to future vaccinations.

## 6 Vaccinology for seasonal influenza vaccines

### 6.1 Key progress

The focus of this area is to optimize seasonal influenza vaccines. Three milestones have been accomplished: development of new potency assays to ensure timely release of annual vaccine formulations [57,58]; identification of preliminary lessons learned from the COVID-19 pandemic that are applicable to influenza vaccine R&D [59]; and documentation of at least two combined COVID-19 and seasonal influenza vaccines in clinical development [13], with one combination COVID-19/influenza mRNA vaccine completing a phase 3 clinical trial [60] and under regulatory review [61].

Improving seasonal influenza vaccines involves continuing the development of egg-independent platforms (including mRNA approaches), generating reverse genetics systems that can rapidly yield recombinant virus strains for vaccine production, and identifying new vaccine adjuvants. Recent clinical evaluations of the safety, effectiveness, and immunogenicity of next-generation seasonal vaccines have found that cell-based, mRNA, and recombinant vaccines elicit similar immune responses, including in children and older adults, compared with egg-based vaccines, and avoid the potential for egg-based mutations that may decrease effectiveness [62] [[63], [64], [65]]. Interest in the use of novel platforms—particularly influenza virus-based, mRNA, nanoparticles, and virus vectors—has led to 13 next-generation influenza vaccines and seven COVID/influenza combination vaccines in active clinical development as of the time of writing this report [13].

Adjuvants added to influenza vaccine formulations can play an important role in boosting the potency and breadth of vaccine-induced immune responses and can enable dose-sparing, which may be particularly important in LMICs by reducing both unit and administration costs. Two high-priority milestones call for evaluating the use of adjuvants in high-risk populations (i.e., older adults and the very young). Several clinical trials have found that adjuvants can safely elicit enhanced humoral and cellular immune responses and durable antibody seroprevalence to circulating and historical strains in older adults [[66], [67], [68]].

Inclusion of NA antigens may play an important role in influenza vaccine effectiveness by inducing immune responses that reduce disease severity and provide cross-protection, but since current seasonal influenza vaccines are designed to contain a standardized amount of HA, neither the NA content nor NA quality are evaluated [69]. A high-priority milestone is to determine whether the presence of NA improves seasonal vaccines and if so, to establish the optimal NA dose. A recent study of NA antigenic profiles for H1N1 and H1N1pdm09 virus circulating in humans showed broader cross-reactivity of anti-NA antibody responses when compared with the anti-HA response, supporting inclusion of NA in influenza vaccine preparations [70]. A household transmission study in Nicaragua showed that vaccines designed to elicit NA immunity in addition to HA immunity may prevent infection and reduce infectivity [71]. Data from numerous preclinical studies suggest the potential for NA antigens to enhance immunogenicity of different influenza vaccine constructs [13].

### 6.2 Persistent challenges

Although progress has been made in developing assays for measuring NA content in influenza vaccines, further work is needed on NA assay development before the antigenic variation of NA in seasonal vaccines can be determined and before methods can be established to confirm, measure, and standardize NA content in influenza vaccines; no progress was identified for these two milestones.

Evaluating the effectiveness of new approaches for seasonal influenza vaccines requires strengthening clinical trial partnerships across geographies and sectors to develop next-generation influenza vaccines and test combinations of licensed seasonal vaccines and adjuvants that are suitable for use in low-resource settings and in diverse age groups. Another major challenge is determining how best to assess the relative effectiveness of new vaccines vs. existing vaccines or vs. each other. The National Academy of Medicine's 2022 consensus report provides recommendations to optimize vaccine R&D to support the prevention and control of seasonal and pandemic influenza, based on the experience with COVID-19 vaccine development and deployment [72].

## 7 Vaccinology for broadly protective or universal influenza vaccines

### 7.1 Key progress

**Fig. 3:**
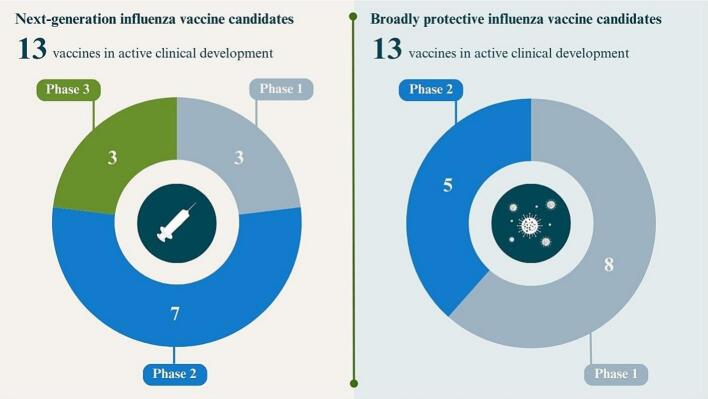
Next generation and broadly protective influenza vaccine candidates in clinical development, May 2025. Description: This figure uses 2 circle graphs to show how many candidate influenza vaccines—next generation or broadly protective—were in Phase 1, Phase 2, or Phase 3 clinical trials as of mid-May 2025.

Milestones in this area aim to move broadly protective influenza vaccines through the vaccine development pipeline toward licensure, as exemplified by a Chemistry, Manufacturing, and Controls framework developed by the Coalition for Epidemic Preparedness Innovations [73]. One high-priority milestone aims to continue research on new antigen design to facilitate the development of vaccines with broad-based protection. Two high-priority milestones have been accomplished, both of which facilitate the progression of broadly protective vaccine candidates through clinical trials. Thirteen broadly protective candidates are currently in active clinical development (Fig. 3).

### 7.2 Persistent challenges

Development of broadly protective vaccines builds on efforts to advance seasonal influenza vaccinology, as it applies to advances in next-generation technology and efforts to improve manufacturing capacity and vaccine access in LMICs. Despite this shared vaccinology ecosystem, important questions remain regarding antigen design and methods to evaluate immunogenicity, efficacy, and durability of vaccine candidates against viral strains that do not yet exist or circulate in humans. Further work is needed to assess issues such as impact of partial immunity on immune response to a broadly protective vaccine, clarify regulatory pathways for approval, and define endpoints (including clinical endpoints) for determining durability and efficacy in populations at risk of severe disease or lowered immune response.

## 8 Animal models and the controlled human influenza virus infection model

### 8.1 Key progress

This area's milestones outline steps to optimize animal models and the CHIVIM for influenza vaccine R&D. Two milestones that assess progress in expanding availability of animal-model resources have been partially accomplished, with NIAID networks developing reagents and data standards for influenza vaccine research and, in fulfillment of a high-priority milestone, providing free access to more than 1000 unique immunological reagents for the ferret model [[74], [75], [76]]. Progress has been made in determining the predictive value of macaque, swine, and ferret models in clarifying dose optimization [77,78] and the impact of vaccination on maternal immunity and fetal development [79]. Additionally, novel mouse models for evaluating immune responses and for screening T-cell epitopes have been generated [80,81]. Other in-progress milestones are supported by efforts to develop best practices for assessing viral transmission in ferrets [82], refine the ferret model to assess the role of airborne and aerosol transmission [83], and clarify the role of obesity in influenza pathogenesis using a diet-induced ferret obesity model [84].

One high-priority milestone to generate guidance for the CHIVIM has been accomplished with the development of ethical considerations for CHIVIM studies [85,86] and discussion of the role of CHIVIM studies to inform vaccine evaluation and regulatory approval [87,88]. One other CHIVIM high-priority milestone is in progress and entails ensuring the availability of challenge viruses, cell lines, and human samples [89].

### 8.2 Persistent challenges

Additional work is needed to comprehensively analyze the predictive value of different animal models for influenza vaccine studies. Also, further efforts are needed to develop standardized protocols and data-sharing mechanisms for conducting multicenter CHIVIM studies.

## 9 Policy, financing, and regulation

### 9.1 Key progress

Advancing influenza vaccine R&D requires strategies for promoting innovation and catalyzing broad support (including sustainable funding), exploring regulatory challenges associated with licensure of broadly protective influenza vaccines, and enhancing information sharing. Flu Hub, developed by NIAID, provides a new central repository for information on a range of influenza research resources, including research facilities, preclinical services, clinical resources, reagents, datasets, and bioinformatics tools [90]. Additionally, five milestones have been fully or partially accomplished: 1) development of the Universal Influenza Vaccine Technology Landscape [13,15]; 2) development of the IVR Funding Tracker [91], accessible on the IVR Initiative website; 3) the availability of basic research and clinical trial data, along with specific reagents, via NIAID's ImmPort platform [92]; 4) identification of lessons learned from the COVID-19 pandemic that are relevant to the sustainable or emergency development of improved influenza vaccines [93,94]; and 5) development of an influenza severity assessment for adults hospitalized with influenza [95].

In progress toward two high-priority milestones, global initiatives are underway that characterize the individual and societal value of safe and effective influenza vaccines. WHO is developing a full value of improved influenza vaccine assessment (FVIVA) intended to clarify use cases, market and deployment potential, and expected health and economic impacts with next-generation vaccines [6]. In support of this work, a growing body of research highlights the need for improved influenza vaccines to be cost-effective regarding their projected effects on infection burden, quality of life, caregiver requirements, national economies, and children's ability to thrive, particularly in LMICs [[96], [97], [98]]. With the aim of strengthening vaccine production infrastructure and the transition from annual influenza immunization campaigns to the rollout of broadly protective and durable vaccines when available, some progress has been made in technology transfer and alternative vaccine formulations (e.g., microneedle patches and aerosol formulations) suitable for use in low-resource areas [99,100], along with recent assessments of vaccine production capacity and the projected effects of supply and demand on equitable geographic access to seasonal and pandemic vaccines [101,102].

Broad scientific, economic, and regulatory support for all stages of the vaccine development process is crucial to facilitate progress of promising candidates through clinical evaluation, regulatory review, and licensure, particularly if evaluation includes alternatives to traditional phase 3 efficacy trials, such as immunobridging and postmarketing studies. In progress toward two high-priority milestones, mechanisms for international sharing of viruses for vaccine production have come under scrutiny after significant delays with requesting SARS-CoV-2 and influenza strains and sequences under the requirements of the Nagoya Protocol on Access to Genetic Resources and the Fair and Equitable Sharing of Benefits Arising from the Utilization to the Convention on Biological Diversity [[103], [104], [105]]. These efforts include advocacy for shortening the time from request to receipt for important pathogens or for exemption from the Nagoya Protocol [106,107]. Additionally, during the 16th meeting of the Conference of the Parties to the Convention on Biological Diversity, countries agreed on operationalization of a new global mechanism to share benefits from the use of digital genetic information [108].

In evidence for a related high-priority milestone, regulators, researchers, and funding organizations have begun incorporating regulatory science issues and pathways into guidance for clinical trials, vaccine R&D and evaluation, and pandemic influenza planning [[109], [110], [111]]. Two workshops have addressed regulatory issues regarding CHIVIM studies, adding to the ongoing development of consensus on how CHIVIM studies might be used to provide data for licensure of broadly protective vaccines [112,113].

### 9.2 Persistent challenges

More effort is needed to explore the feasibility of long-term public-private partnerships for developing broadly protective influenza vaccines. No progress was made on several milestones that require broad consensus on complex issues such as sharing intellectual property, harmonizing data standards and exploring mechanisms for data sharing, and developing approaches for clinical methodologies to demonstrate effectiveness of broadly protective influenza vaccines. Further work is also needed to review regulatory options and define the basis on which broadly protective influenza vaccines could be licensed, including approaches for using correlates of protection, immunobridging, and phase 4 confirmatory studies to de-risk efficacy evaluation.

## 10 Conclusion

Since launching the IVR Initiative in September 2021, CIDRAP has monitored progress toward meeting the specific milestones outlined in the IVR. The data summarized here indicate that important progress has been made on more than 80% of all milestones. Influenza vaccine R&D activities are ongoing, as shown by a robust pipeline for universal, broadly protective, and next-generation influenza vaccines [13,15].

Despite the many successes documented in this report and the vast array of scientific publications and ongoing research outlined in Appendix A (supplemental material), major hurdles still need to be overcome to realize the goals of improving seasonal vaccines and generating broadly protective influenza vaccines. Although many of the IVR milestones are in progress, fully completing them will require ongoing extensive research and resources. Because influenza viruses circulate seasonally each year, repeated influenza virus exposures and infections are common. Understanding the interplay of responses to initial infection (i.e., immune imprinting), repeat exposures to influenza viruses, and annual vaccinations on immune responses to vaccines by age group and by vaccine product requires prospective longitudinal studies. Additionally, improving influenza vaccines requires resolving fundamental immunologic issues, such as determining how durable immunity is achieved and maintained; identifying ways to stimulate broadly protective immune responses; and enhancing knowledge about mucosal immunity in protecting against influenza virus infection, disease, and transmission.

No progress was identified for 10 IVR milestones (see Appendix A for details). The reasons for lack of progress likely vary and remain speculative; however, several possible explanations are 1) some of the milestones cannot be moved forward until other issues are addressed, such as developing standardized assays for measuring NA, which are needed before resolving other NA-related milestones; 2) the scientific challenges for some milestones are considerable, such as developing correlates of protection to assess broadly protective influenza vaccines; 3) some milestones were likely not considered high enough priority to garner limited resources, with only one of these milestones ranked as high priority; and 4) several milestones require a consensus-driven process; such processes can be resource intensive and financially prohibitive without targeted funding to move them forward.

Ongoing political will and global coordination are needed to sustain the resources necessary to advance influenza vaccine candidates. This requires advocacy to inform and influence policy makers and key stakeholders. Additionally, growth in the global demand for seasonal influenza vaccines will serve as a pull mechanism for greater investment in influenza vaccine R&D. Given the myriad of competing global health priorities and current political landscapes, sustaining adequate support for the diverse range of scientific research required will be an ongoing challenge.

Limitations of this monitoring program include the following. Although we conducted extensive searches of available literature and other information and we engaged a wide network of global experts, important research developments may have been inaccessible to us, including non-English-language sources and proprietary information from the vaccine industry. Furthermore, important research studies that could contribute to influenza vaccine development may be occurring in other fields of research not detected via our methodology. Additionally, many of the IVR milestones are recorded as “in progress” and it may be unclear how far along a particular milestone may be toward being achieved. While progress is being made, some milestones will require ongoing support and additional research over time to be fully accomplished.

The IVR is intended to be a living document and has been reviewed and revised over the initial 3-year IVR ME&A program. Periodic future review and reporting on progress toward the IVR goals and milestones will continue to help guide influenza vaccine R&D activities, particularly by identifying R&D areas that need more support and coordination among researchers and funders. Through ongoing attention and investment to address remaining gaps and barriers, important progress will continue toward development of improved influenza vaccines to reduce the global burden of influenza and enhance preparedness for the next influenza pandemic.

## CRediT authorship contribution statement

**Julia T. Ostrowsky:** Writing – review & editing, Writing – original draft, Investigation, Conceptualization. **Natalie C. Vestin:** Writing – original draft, Data curation. **Angela J. Mehr:** Writing – review & editing, Conceptualization. **Angela K. Ulrich:** Writing – review & editing, Conceptualization. **Lauren Bigalke:** Writing – review & editing, Investigation. **Joseph S. Bresee:** Writing – review & editing, Conceptualization. **Martin H. Friede:** Writing – review & editing, Conceptualization. **Bruce G. Gellin:** Writing – review & editing, Conceptualization. **Keith P. Klugman:** Writing – review & editing, Conceptualization. **Usman N. Nakakana:** Writing – review & editing, Conceptualization. **Tian Yun Wang:** Writing – review & editing, Conceptualization. **Charlotte L. Weller:** Writing – review & editing, Conceptualization. **Michael T. Osterholm:** Writing – review & editing, Conceptualization. **Eve M. Lackritz:** Writing – review & editing, Conceptualization. **Kristine A. Moore:** Writing – review & editing, Writing – original draft, Supervision, Project administration, Methodology, Funding acquisition, Conceptualization. **William Ampofo:** Writing – review & editing, Conceptualization. **Edward Belongia:** Writing – review & editing, Conceptualization. **Marco Cavaleri:** Writing – review & editing, Conceptualization. **Cheryl Cohen:** Writing – review & editing, Conceptualization. **Benjamin Cowling:** Writing – review & editing, Conceptualization. **Rebecca Jane Cox:** Writing – review & editing, Conceptualization. **Swati Gupta:** Writing – review & editing, Conceptualization. **Ian Gust:** Writing – review & editing, Conceptualization. **Scott E. Hensley:** Writing – review & editing, Conceptualization. **Irina Isakova-Sivak:** Writing – review & editing, Conceptualization. **Kari Johansen:** Writing – review & editing, Conceptualization. **Stacey Knobler:** Writing – review & editing, Conceptualization. **Florian Krammer:** Writing – review & editing, Conceptualization. **John C.W. Lim:** Writing – review & editing, Conceptualization. **John McCauley:** Writing – review & editing, Conceptualization. **Richard Pebody:** Writing – review & editing, Conceptualization. **Punnee Pitisuttithum:** Writing – review & editing, Conceptualization. **Rino Rappuoli:** Writing – review & editing, Conceptualization. **Tiago Rocca:** Writing – review & editing, Conceptualization. **Stacey Schultz-Cherry:** Writing – review & editing, Conceptualization. **Ethan C. Settembre:** Writing – review & editing, Conceptualization. **James Southern:** Writing – review & editing, Conceptualization. **Kanta Subbarao:** Writing – review & editing, Conceptualization. **John S. Tam:** Writing – review & editing, Conceptualization. **Rajeev Venkayya:** Writing – review & editing, Conceptualization.

## Funding

The work for this project was completed with funding from the 10.13039/100010269Wellcome Trust (Wellcome grant # 225160/Z/22/Z).

## Declaration of competing interest

The authors declare the following financial interests/personal relationships that may be considered as potential competing interests. Julia Ostrowsky reports financial support was provided by Wellcome Trust. Lauren Bigalke, Eve Lackritz, Angela Mehr, Kristine Moore, Michael Osterholm, Angela Ulrich, Natalie Vestin reports financial support was provided by Wellcome Trust. Swati Gupta reports financial support was provided by International Aids Vaccine Initiative. Julia Ostrowsky reports travel was provided by Wellcome Trust. Lauren Bigalke, Eve Lackritz, Angela Mehr, Kristine Moore, Michael Osterholm, Angela Ulrich reports travel was provided by Wellcome Trust. William Ampofo, Edward Belongia, Rebecca Cox, Martin Friede, Bruce Gellin, Kari Johansen, Keith Klugman, Stacey Knobler, Florian Krammer, John McCauley, Punnee Pitisuttithum, Tiago Rocca, Stacey Schultz-Cherry, Ethan Settembre, Kanta Subbarao, John Tam reports travel was provided by Wellcome Trust. Benjamin Cowling reports a relationship with AstraZeneca Pharmaceuticals LP that includes: consulting or advisory. Benjamin Cowling reports a relationship with Fosun Pharma USA Inc that includes: consulting or advisory. Benjamin Cowling reports a relationship with GlaxoSmithKline Inc that includes: consulting or advisory. Benjamin Cowling reports a relationship with Haleon plc that includes: consulting or advisory. Benjamin Cowling reports a relationship with Moderna Inc that includes: consulting or advisory. Benjamin Cowling reports a relationship with Novavax Inc that includes: consulting or advisory. Benjamin Cowling reports a relationship with Pfizer Inc that includes: consulting or advisory. Benjamin Cowling reports a relationship with Roche that includes: consulting or advisory. Benjamin Cowling reports a relationship with Sanofi that includes: consulting or advisory. Ian Gust reports a relationship with CLS Services Ltd. that includes: equity or stocks. Scott Hensley reports a relationship with Sanofi that includes: consulting or advisory. Scott Hensley reports a relationship with Pfizer that includes: consulting or advisory. Scott Hensley reports a relationship with Lumen Bioscience Inc that includes: consulting or advisory. Scott Hensley reports a relationship with Novavax Inc that includes: consulting or advisory. Scott Hensley reports a relationship with Merck & Co Inc that includes: consulting or advisory. John McCauley reports a relationship with Sanofi Pasteur Inc that includes: consulting or advisory and travel reimbursement. Rafeev Venkayya reports a relationship with Coalition for Epidemic Preparedness Innovations UK Limited that includes: board membership. Florian Krammer reports a relationship with National Institutes of Health National Institute of Allergy and Infectious Diseases that includes: funding grants. Florian Krammer reports a relationship with FluLab that includes: funding grants. Florian Krammer reports a relationship with The Gates Foundation that includes: funding grants. Florian Krammer reports a relationship with National Institutes of Health National Cancer Institute that includes: funding grants. Florian Krammer reports a relationship with VIR that includes: funding grants. Florian Krammer reports a relationship with Dynavax Technologies Corporation that includes: funding grants. Florian Krammer reports a relationship with Pfizer that includes: consulting or advisory. Florian Krammer reports a relationship with Seqirus Inc that includes: consulting or advisory. Florian Krammer reports a relationship with Avimex Laboratories that includes: consulting or advisory. Florian Krammer reports a relationship with Third Rock Ventures LLC that includes: consulting or advisory. Florian Krammer reports a relationship with GSK that includes: consulting or advisory. Scott Hensley has patent Universal influenza vaccine using nuceloside-modified mRNA pending to Penn State. Scott Hensley has patent mRNA vaccine with hemagglutinin antigens from every influenza virus subtype pending to Penn State. Florian Krammer has patent #SARS-CoV-2 serological assays pending to Icahn School of Medicine at Mount Sinai. Florian Krammer has patent #NVD-based SARS-CoV-2 and influenza vaccines pending to Icahn School of Medicine at Mount Sinai. Florian Krammer has patent Influenza virus therapeutics pending to Icahn School of Medicine at Mount Sinai. Florian Krammer reports that he is an editor-in-chief for the journal, Vaccine. Rebecca Jane Cox reports that she is Deputy Chair of the International Society of Influenza and Other Respiratory Virus Diseases; she also advises the WHO, EU and EMA on respiratory viruses and vaccines. Tian Yu Wang reports she is employed by Wellcome Trust, which funded the project. Charlotte Weller reports that she is employed by Wellcome Trust, which funded the project. If there are other authors, they declare that they have no known competing financial interests or personal relationships that could have appeared to influence the work reported in this paper.

## Data Availability

No data was used for the research described in the article.

## Appendix A Supplementary data

Supplementary material: Summary of Research Outcomes Addressing the IVR Strategic Goals and Milestones Image 1
