# Supplementary material for: Accomplishments and challenges in developing improved influenza vaccines: An evaluation of three years of progress toward the milestones of the influenza vaccines research and development roadmap
Source: Vaccine. 2025 Aug 13;61:None. doi: 10.1016/j.vaccine.2025.127431 (PMC12447092; doi:10.1016/j.vaccine.2025.127431)
Supplement: Supplementary file 1 — Supplementary material: Summary of Research Outcomes Addressing the IVR Strategic Goals and Milestones [file mmc1.docx]

## **Center for Infectious Disease Research and Policy (CIDRAP)**

## **Influenza Vaccines R&D Roadmap (IVR) Monitoring, Evaluation, and Adjustment (ME&A)**

## **Summary of Research Outcomes Addressing the IVR Strategic Goals & Milestones**

Updated May 2025

This document provides detailed information on R&D progress toward achieving the milestones in each topic area of the Influenza Vaccines R&D Roadmap (IVR) since it was first published (September 2021), based on R&D activities and outcomes reported as of December 2024. Citation links are provided when available. Statements that are not referenced reflect additional input provided by IVR steering group and taskforce members. The IVR strategic goals and milestones were revised in early 2025 based on a review by the full taskforce and steering group.

For assessment purposes, the status of R&D progress toward each milestone is summarized in one of three categories:

- ACCOMPLISHED: Milestone has been accomplished, fully or partially
- IN PROGRESS: Relevant research outcomes reported, indicating progress toward the milestone
- NO PROGRESS: No relevant research outcomes identified, indicating no progress toward the milestone since September 2021

**TOPIC 1: VIROLOGY APPLICABLE TO VACCINE DEVELOPMENT**

| ***Strategic Goal 1.1:*** Improve understanding of human and animal influenza virus evolution. |
| --- |
| *High-Priority Milestone*  **Milestone 1.1.a***:* *Refine strategies to improve efficiencies in obtaining sequences and isolates of circulating influenza viruses in human populations and a broad range of animal species, including species not previously recognized as important for influenza, such as bovine.*  IN PROGRESS   - As an extension of ongoing of sampling strategies and assessment, data will be derived from the WHO Global Influenza Surveillance and Response System ([GISRS](https://www.who.int/initiatives/global-influenza-surveillance-and-response-system)); additional sources include the [WHO Global Influenza Programme](https://www.who.int/teams/global-influenza-programme/surveillance-and-monitoring), the [Global Influenza Hospital Surveillance Network](https://www.gihsn.org/) (GIHSN), and the [WHO Collaborating Centers](https://www.who.int/initiatives/global-influenza-surveillance-and-response-system/who-collaboration-center-erl?CxitPEOtTWx0xUd5TJdODSXcnyJqzYd7FZeivpn7xcI=).  - [WHO 2024](https://www.who.int/publications/i/item/9789240101432) provided updated guidance on surveillance standards and operational approaches for an expanded GISRS that aims to strengthen national sentinel surveillance capacities for respiratory viruses and improve preparedness and response to future health emergencies caused by respiratory viruses. - Additional sources of data relevant to progress toward this milestone may include Africa CDC [Institute for Pathogen Genomics (IPG)](https://africacdc.org/institutes/ipg/) and [PAHO](https://www.paho.org/en/topics/influenza-and-other-respiratory-viruses) collaborations on surveillance for influenza and other respiratory diseases. - The US CDC has provided funding and technical support to WHO GISRS for surveillance, global guidance, research on transmission zones, and regional network activities. - NIAID [Centers of Excellence for Influenza Research and Response (CEIRR)](https://www.ceirr-network.org/) program supports research on influenza virus evolution, emergence, transmission, and pathogenicity, with an international research infrastructure to respond to emerging and pandemic influenza. |
| **Milestone 1.1.b**: *Convene a group of stakeholders to determine how best to use new tools, such as computational approaches, machine learning, and systems biology, to enhance understanding of influenza virus evolution and to improve capabilities to predict circulating influenza virus strains, including emergence of novel viruses.*  IN PROGRESS   - [Huddleston 2024](https://www.medrxiv.org/content/10.1101/2024.09.11.24313489v1) quantified the effects of reducing forecast horizons and submission lags on the accuracy of forecasts for A/H3N2 populations to determine whether technologies for more rapid vaccine development could improve long-term forecasts for seasonal influenza. Results showed the potential to improve the accuracy of existing influenza forecasting models by using new influenza vaccine platforms such as mRNA and increasing global sequencing capacity. - [Kim 2024](https://pubmed.ncbi.nlm.nih.gov/37961288/) used a cross-sectional antibody-derived estimate of population susceptibility to different clades of influenza A (H3N2) could predict the success of clades in the following season and found that representative measures of population immunity might improve evolutionary forecasts and inform selective pressures on influenza. - [Parino 2024](https://pubmed.ncbi.nlm.nih.gov/39737444/) developed a multiscale epidemiological model calibrated on worldwide genetic data through phylogeographic inference, simulating migration fluxes between epidemics occurring in different countries and identifying model parameterizations to predict global influenza virus circulation. - [Welsh 2024](https://www.cell.com/cell-host-microbe/fulltext/S1931-3128(24)00233-6) used use deep mutational scanning to map how mutations to HA proteins of two H3N2 strains affect neutralization by serum from individuals of a variety of ages, to investigate how antigenic effects of viral mutations vary across the human population and how this heterogeneity affects virus evolution. |
| **Milestone 1.1.c**: *Integrate research to develop and implement a plan to improve genetic and antigenic characterization of emerging and circulating influenza viruses (including epitopes beyond HA and NA, such as T-cell epitopes), using techniques such as deep-sequencing technology, computational biology, and phylogenetic analysis. This plan should outline specific goals for how analytic characterization can be used to predict antigenic drift. Ongoing screening of emerging strains with a standardized, regularly updated panel of monoclonal antibodies (mAbs) binding key epitopes could also be considered as part of this effort.*  NO PROGRESS |
| *High-Priority Milestone*  **Milestone 1.1.d**: *Develop a comprehensive landscape of population-based serosurveillance studies in different age groups and geographic regions to better understand the relationship between population immunity to seasonal influenza viruses and antigenic drift, which is needed to determine how serosurveillance data can be used to improve decisions regarding vaccine strain selection for seasonal influenza vaccine production.*  IN PROGRESS   - [Guiomar 2024](https://www.ncbi.nlm.nih.gov/pmc/articles/PMC11128746/) conducted the first annual seroprevalence survey from 2014 to 2019 of infection- and vaccination-induced protective Abs against influenza A(H1N1)pdm09, A(H3N2), B/Victoria, and B/Yamagata in Portugal to assess the seroprevalence of protective Abs against influenza virus and to evaluate the correlation of seroprevalence measured in summer with the influenza like-illness (ILI) rate in the following influenza season. Researchers found significant differences in the seroprevalence of protective Abs against influenza in the population, according to age, vaccination status, and geographic region, providing insight into the relationship between the seroprevalence of protective Abs against influenza and the reduction of the ILI incidence rate in the next season. |
| ***Strategic Goal 1.2:*** Enhance the ability to forecast viruses that are likely to circulate in the upcoming season to improve the antigenic match between circulating influenza viruses and viral strains selected for vaccine production. |
| **Milestone 1.2.a***: Determine how to use genetic sequence data and technologies for modeling and forecasting to improve the antigenic match between vaccine strains and circulating strains, and develop methods to improve forecasting of circulating viruses.*  IN PROGRESS |
| - [Cai 2024](https://pubmed.ncbi.nlm.nih.gov/38498763/) developed the FluPMT model that integrates virus mutation temporal information and antigenic information using multi-task learning to predict the predominant strains of the following year and identify key residue-level factors driving viral evolution in HA sequences. Use of the model on two influenza datasets demonstrated the predictive performance of FluPMT. - [Catani 2024](https://pubmed.ncbi.nlm.nih.gov/38805550/) examined how NA accumulates mutations and evaluated how these mutations affect immune responses; analyzed the antigenic diversity of a panel of N2 NAs derived from H3N2 viruses circulating between 2009 and 2017, identifying at least four major phylogenetic groups; determined that amino acid residues in N2 NA near the catalytic site have a major impact on NAI susceptibility by immune sera; and developed a machine learning method to predict the impact of mutations in the N2 NA panel on NAI. - [Shah 2024](https://pubmed.ncbi.nlm.nih.gov/38714654/) developed a machine learning model to predict (normalized) outputs of HI assays involving circulating human IAV H3N2 viruses, using their HA1 sequences and associated metadata. The model distinguishes antigenic variants from non-variants and adaptively characterizes seasonal dynamics of HA1 sites having the strongest influence on antigenic change. - [Hayati 2023](https://pubmed.ncbi.nlm.nih.gov/37922357/) trained machine-learning models to predict which sequences are most likely to grow during the upcoming influenza season based on features of phylogenetic trees and explored choices for sequences to be considered for inclusion in the following year’s seasonal influenza vaccine. - [Lee 2023](https://pubmed.ncbi.nlm.nih.gov/37068231/) created an interactive visualization tool to inform the analysis of influenza virus evolution by displaying serologic data in a phylogenetic context, which enables direct comparison of antigenic distances between vaccine candidates. - [Liu 2023](https://pubmed.ncbi.nlm.nih.gov/37211247/) developed PREDAC-H1pdm, a model to predict antigenic relationships between H1N1pdm viruses and identify antigenic clusters for post-2009 pandemic H1N1 strains. - [Perofsky 2023](https://pubmed.ncbi.nlm.nih.gov/37873362/) compared experimental and sequence-based measures of A(H3N2) evolution in predicting regional epidemic dynamics in the United States across 22 seasons, from 1997 to 2019, and considered the effects of other co-circulating influenza viruses, prior immunity, and vaccine-related parameters, such as coverage and effectiveness, on A(H3N2) incidence. Results indicated that evolution in HA and NA contributes to variability in epidemic magnitude across seasons, though viral fitness appears to be secondary to subtype interference in shaping annual outbreaks. - [Thadani 2023](https://pubmed.ncbi.nlm.nih.gov/37821700/) developed [EVEscape](https://evescape.org/), a computational method for predicting the likelihood of antibody escape for viral mutations, including influenza HA mutations. - [NIAID CEIRR](https://www.ceirr-network.org) is supporting research on the evolutionary dynamics of influenza to predict evolutionary trajectories of circulating viruses and [improve strain selection for seasonal influenza vaccines](https://grants.nih.gov/grants/guide/rfa-files/RFA-AI-20-055.html); development of tools to assess selection pressure and viral fitness; and development of models and platforms for predicting future dominant seasonal virus strains. |
| **Milestone 1.2.b**: *Reconvene the World Health Organization (WHO) expert group on improving influenza vaccine virus selection to review activities for improving the antigenic match between circulating viruses and annual vaccine seed strains, as outlined in 2015 by the WHO; develop consensus on new strategies to improve forecasting; and update the 2015 consensus report as needed to reflect new developments and strategies.*  NO PROGRESS |
| *High-Priority Milestone*  **Milestone 1.2.c:** *Continue to develop, harmonize, and implement methods (e.g., the use of predictive artificial intelligence and other new technologies) to inform antigenic characterization of H1N1 and H3N2 viruses.*  IN PROGRESS   - [Huang 2024](https://pubmed.ncbi.nlm.nih.gov/39205645/) used a pseudovirus platform to evaluate the alignment between the 2023 and 2024 seasonal influenza vaccine H3N2 strain (A/Darwin/6/2021) and currently circulating influenza strains. - [Harvey 2023](https://pubmed.ncbi.nlm.nih.gov/36972311/) developed a new approach using a Bayesian model for integrating genetic and antigenic data to identify genetic changes in H3N2 virus that underpin antigenic drift. - [Peng 2023](https://pubmed.ncbi.nlm.nih.gov/37515165/) developed a novel quantitative prediction method to predict the antigenic distance between virus strains using attribute network embedding techniques. Results show a strong positive correlation between supplementing genetic features and antigenic distance prediction accuracy. - [Galli 2022](https://www.mdpi.com/2076-393X/10/8/1359) developed and described a high-throughput whole-genome sequencing protocol for A(H3N2) viruses, providing a fast and accurate method to characterize the complete genome of H3N2 viruses directly from clinical respiratory samples. - The US CDC is developing a human epitope map to identify escape variants. Researchers have screened hundreds of Abs for inclusion in a library of human mAbs that recognize antigenic sites of the A(H1N1)pdm09 HA molecule, and mAbs have been characterized and mapped to antigenic sites in the HA. Work continues to add to this panel of reagents to generate a comprehensive library of mAbs that map the antigenic sites of contemporary A(H1N1)pdm09 influenza viruses. - The US CDC is characterizing NA antigenicity of A(H3N2) viruses, which includes optimizing methods for analysis and assessing contemporary A(H3N2) viruses as part of the WHO vaccine recommendation process. |
| ***Strategic Goal 1.3:*** Improve the ability to detect and understand the emergence of novel influenza viruses with pandemic potential. |
| **Milestone 1.3.a**: *Expand surveillance of influenza viruses at the human-animal interface, including in wild birds and in Africa, Asia, and South America, to identify geographic surveillance gaps, highlight the need for coordination among international groups, improve understanding of the emergence of novel and potentially pandemic viruses in animal reservoirs, and promote data sharing and integration across different surveillance systems. Social scientists should be involved in planning and implementing human-animal interface studies.*  IN PROGRESS   - Key organizations with active surveillance systems include [OFFLU](https://www.offlu.org/) (Global Network of Expertise on Animal Influenza), the UK [Flu-MAP](https://gtr.ukri.org/projects?ref=BB%2FX006204%2F1) project (monitoring HPAI in wildlife and domestic animals in the UK and Europe), [FAO](https://www.fao.org/animal-health/animal-diseases/avian-flu-qa/en#:~:text=FAO%20is%20closely%20monitoring%20avian,sources%2C%20including%20OIE%20and%20WHO.) (tracking circulating animal viruses, One Health emphasis), the WHO, and/or the NIAID [CEIRR](https://www.ceirr-network.org/) network. |
| **Milestone 1.3.b**: *Identify funding and implement the expanded surveillance plan detailed in Milestone 1.3.a (to be funded for at least 5 years). One option is to create a coordinated global network of centers with the capability to sample wild birds, domestic animals, and humans at the human-animal interface to test for influenza viruses.*  IN PROGRESS   - The [CEIRR](https://www.ceirr-network.org/) network, established in 2021 and funded through 2028, supports projects to determine how influenza viruses evolve, adapt and transmit between humans and at interspecies interfaces, including conducting global surveillance of influenza viruses at the human-animal interface. |
| ***Strategic Goal 1.4:*** Enhance understanding of factors associated with viral transmissibility. |
| *High-Priority Milestone*  **Milestone 1.4.a**: *Review optimal study designs for evaluating influenza transmission, including controlled human influenza virus infection model (CHIVIM) studies and household and school-based transmission studies.*  IN PROGRESS   - [Lane 2024](https://pubmed.ncbi.nlm.nih.gov/39440405/) summarized discussions and outcomes from NIAID’s workshop (*CHIVIM studies: current status and future directions for innovation*, Nov. 13-14, 2023), which addresses issues regarding the feasibility of conducting transmission studies using CHIVIM studies. - [Shetty 2024](https://pubmed.ncbi.nlm.nih.gov/39589151/) used a CHIVIM with H3N2 seasonal influenza virus to study influenza transmission, including symptom progression and the dynamics of virus shedding, to inform the design of future challenge studies focused on modeling and limiting transmission. - [NIAID supported EMIT-2 study](https://reporter.nih.gov/search/u57CqGLe4km26bTNP2h0jA/project-details/10645155) has developed and implemented analytical models of influenza transmission to optimize a hotel quarantine facility for conducting CHIVIMs that mitigates the risk of unwanted transmission outside of the experimental environment. - [Belser 2023](https://pmc.ncbi.nlm.nih.gov/articles/PMC9997909/) analyzed practical obstacles and limitations on performing virus transmission studies in laboratory environments, the additional challenges posed by conducting these experiments concurrent with in vivo experimentation, and how continued investment in this work will provide greater understanding of the role aerosols play in viral transmission. - [Nguyen-Van-Tam 2020](https://pubmed.ncbi.nlm.nih.gov/32658939/) conducted a human influenza transmission challenge study to assess the importance of aerosol transmission in human-to-human transmission of influenza viruses. |
| **Milestone 1.4.b**: *Conduct at least two comprehensive influenza virus transmission studies in animals (to include naive and infected or vaccinated animals).*  IN PROGRESS   - [Septer 2024](https://journals.asm.org/doi/10.1128/mbio.02161-24) used a ferret model to assess if vaccine-induced NA immunity could disrupt transmission of the 2009 pH1N1 virus. Results showed that 2009 pH1N1 virus transmitted efficiently through chains of transmission in the presence of NA immunity, although NA-vaccinated animals shed significantly less virus and had accelerated viral clearance, demonstrating that vaccine-induced NA immunity is not sufficient to prevent infection via airborne exposure and onward airborne transmission of 2009 pH1N1 virus. - [Hu 2023](https://www.science.org/doi/10.1126/sciadv.adf5182) conducted a comprehensive investigation on H3N2 influenza vaccine reference viruses and circulating viruses using a ferret model to address the contributions of HA stability to vaccine reference virus selection and pandemic risk assessment. - [Belser 2022](https://pubmed.ncbi.nlm.nih.gov/35862762/) used the ferret transmission model to conduct a multicenter standardization exercise to improve the interpretation of influenza transmission data generated during risk assessment activities. - [Pulit-Penaloza 2022](https://pubmed.ncbi.nlm.nih.gov/35537045/) used the ferret model to assess the pathogenesis and transmission capability of four genetically and antigenically diverse swine A(H1) IAVs isolated from humans between 2017 and 2020. |
| **Milestone 1.4.c**: *Conduct at least one modeling study to predict influenza virus transmission across geographic regions and within species (e.g., humans, wild birds, domestic animals, agricultural animals).*  IN PROGRESS   - [Gass 2023](https://pubmed.ncbi.nlm.nih.gov/36239465/) quantified the migratory connectivity of Iceland within the global context and the transmission dynamics that govern the spread of IAVs within Iceland and between adjacent geographic regions, using Bayesian phylogeographic and phylodynamic inference. - [Dai 2022](https://pubmed.ncbi.nlm.nih.gov/35752386/) simulated the infectivity of the main infection subtypes of influenza in China to provide a scientific basis for the prevention and control of different influenza subtypes. |

**TOPIC 2: IMMUNOLOGY AND IMMUNE CORRELATES OF PROTECTION**

| ***Strategic Goal 2.1:*** Promote the development and standardization of immunologic tools to inform the development of universal, broadly protective, and next-generation influenza vaccines. |
| --- |
| **Milestone 2.1.a:** *Develop a comprehensive list of clinical trials globally that are ongoing or planned (such as ongoing cohort studies) and develop and share guidance to support the collection, management, storage, and distribution of clinical samples (including samples from commercial entities) for immunologic research relevant to improved influenza vaccines.*  IN PROGRESS   - The NIH [Flu Hub Cohort Studies](https://www.fluhub.org/resources/cohort) database includes a searchable catalog of DMID-funded and other clinical studies to support research related to the NIAID universal influenza vaccine strategic initiative; resources include study samples (e.g., serum, bone marrow, and nasopharyngeal swabs), which are available to researchers. |
| **Milestone 2.1.b:** *Develop standardized reference reagents and harmonized assay protocols for studying immunoglobulin responses to influenza infection and vaccination, such as qualified assays for detecting mucosal antibodies.*  IN PROGRESS   - [Vincent 2024](https://pmc.ncbi.nlm.nih.gov/articles/PMC11596312/) developed a high-throughput novel modified validated HAI assay for evaluating vaccine immunogenicity and efficacy using egg-derived influenza viruses or recombinant VLPs as agglutinins and human RBCs to measure anti-influenza HA Ab titers in human serum, to test sera from influenza vaccine clinical trials. Results showed that titers from the validated HAI assay correlated well with those of a qualified microneutralization assay. - [Koutsakos 2023](https://pubmed.ncbi.nlm.nih.gov/37564999/) assessed the utility of different types of samples from the human respiratory tract, including nasopharyngeal swabs (for assessing antibodies in nasal mucosa) and bronchoalveolar lavage (for assessing antibodies from the lower respiratory tract). This study informs the value of different sample types for measuring mucosal Abs. - [Waldock 2023](https://pubmed.ncbi.nlm.nih.gov/37143658/) (FLUCOP consortium) conducted a head-to-head comparison of harmonized HAI and MN protocols to evaluate the relationship between HAI and MN titers, and the impact of assay harmonization and standardization on inter-laboratory variability, and agreement between these methods. - [Waldock 2023](https://pubmed.ncbi.nlm.nih.gov/36926342/) (FLUCOP consortium) developed a feasibility study for conducting an external quality assessment scheme for influenza serology methods, to improve performance, reduce inter-laboratory variation, and support the use of harmonized protocols and biological standards for seasonal influenza serology testing. - [Bernard 2022](https://pubmed.ncbi.nlm.nih.gov/35784305/) (FLUCOP Consortium) present a freely available and detailed standard operating procedure for N1 influenza antigen using a validated ELLA-NI. The assay performed consistently with both influenza A and influenza B antigens. - [Waldock 2021](https://pubmed.ncbi.nlm.nih.gov/34319129/) (FLUCOP consortium) assessed the impact of harmonizing the HAI assay protocol/reagents and using standards on interlaboratory variability. - NIAID CIVICs is qualifying assays including secretory IgA MSD to look at mucosal response to novel flu vaccines; Duke, University of Maryland—Baltimore, and Imperial College London have harmonized protocols and are conducting proficiency panels to measure harmonization; reagents, viruses, mAb are available at these sites or in the [BEI Resources](https://www.beiresources.org/) portal. NIAID has also identified mAb panels for use in assays for CIVICs trials. - [Levy 2023](https://pubmed.ncbi.nlm.nih.gov/36567516/) developed a novel antigen microarray-based assay for rapid high-throughput antibody profiling, which can be used for profiling IgG, IgA, and IgM responses to multiple antigens simultaneously, requiring minimal amounts of sample and antigens. - [Rumfelt 2023](https://pubmed.ncbi.nlm.nih.gov/37127782/) developed a luciferase MN assay that combines the advantages of the conventional MN assay with the ease of the HAI assay. - [Sawant 2023](https://pubmed.ncbi.nlm.nih.gov/37090729/) developed a qualified and adaptable influenza serology method and analysis strategy to measure quantifiable HAI titers to define correlates of vaccine-mediated protection in human clinical trials. |
| **Milestone 2.1.c:** *Develop standardized and lower cost assays (e.g., simplified high-throughput testing) for measuring T-cell responses to vaccination or infection.*  IN PROGRESS   - [Waerlop 2023](https://pubmed.ncbi.nlm.nih.gov/37918618/) (FLUCOP Consortium) reported results of two proficiency tests for cellular immune responses, influenza-specific IFN-ɣ ELISpot and ICS assays, organized to evaluate the impact of FLUCOP’s harmonization effort on assay results and the performance of participating FLUCOP partners. - [Begue 2022](https://pubmed.ncbi.nlm.nih.gov/36341380/) (FLUCOP Consortium) developed a harmonized consensus protocol for the ICS assay, which permits quantitative and qualitative evaluation of influenza vaccine-induced T-cell responses and may allow for future comparisons of T-cell responses to different next-generation influenza vaccine candidates. The harmonized ICS assay permits quantitative and qualitative evaluation of influenza vaccine-induced T-cell responses. - [Waerlop 2022](https://www.frontiersin.org/articles/10.3389/fimmu.2022.984642) (FLUCOP Consortium) described the harmonization and qualification of the influenza-specific IFN-ɣ ELISpot assay to detect and qualify vaccine-induced cellular immune responses. |
| ***Strategic Goal 2.2:*** Gain better understanding of human immunology to inform influenza vaccine development through research focused on new tools and technologies. |
| **Milestone 2.2.a***:* *Convene periodic international workshops to identify advances in understanding immune responses to influenza infection and vaccination that can be applied to improving seasonal vaccines and to developing durable, broadly protective or universal vaccines; disseminate a summary report of key findings from each workshop.*  ACCOMPLISHED   - [Reperant 2024](https://pubmed.ncbi.nlm.nih.gov/38561784/) summarized key presentations from the 9^th^ ESWI Conference in Valencia, Spain, 17-20 Sep 2023. - [Krammer 2024](https://pmc.ncbi.nlm.nih.gov/articles/PMC11461279/) summarized outcomes of the 2023 ISIRV meeting on influenza correlates of protection and [Krammer 2020](https://pubmed.ncbi.nlm.nih.gov/31837101/) summarized outcomes of the 2019 meeting. The next ISIRV meeting on influenza correlates of protection will be held in Vienna, Austria, in October 2025. |
| *High-Priority Milestone*  **Milestone 2.2.b***:* *Determine key mechanisms of durability of protective immunity following influenza virus infection, including the discovery of early biomarkers associated with durable immune responses.*  IN PROGRESS   - [Cortese 2025](https://pubmed.ncbi.nlm.nih.gov/39747435/) used systems vaccinology to identify factors influencing the magnitude and durability of Ab responses in humans to H5N1 vaccine with and without AS03 adjuvant. The findings suggested a platelet-associated signature that predicted Ab response longevity, highlighting a conserved mechanism for vaccine durability. - [Arroyo-Diaz 2023](https://pubmed.ncbi.nlm.nih.gov/37699392/) investigated the mechanisms that control lung-resident memory B-cell responses after intranasal influenza virus infection. - [Robinson 2023](https://pubmed.ncbi.nlm.nih.gov/37164016/) analyzed the turnover and persistence of antigen-secreting plasma cells, which produce antibodies that underlie multiple forms of long-lasting immunity, in different tissues and at different ages. Results inform the understanding of the establishment and maintenance of long-lived humoral immunity. - [Sergeeva 2023](https://pubmed.ncbi.nlm.nih.gov/38006063/) evaluated the production of NAI Abs after immunization with seasonal influenza vaccines, the duration of Ab persistence, and the relationship with other quantitative parameters of the immune response to vaccination. - [Palin 2022](https://pubmed.ncbi.nlm.nih.gov/36456737/) reported on outcomes from a NIAID-convened workshop in July 2022 to identify knowledge gaps and research opportunities regarding durable vaccine protection. - [Langley 2022](https://pubmed.ncbi.nlm.nih.gov/35404084/) examined the contribution of memory B-cells to the maintenance of virus-specific antibody levels following acute influenza virus infection in the murine model; data showed that virus-specific plasma cells in the bone marrow are intrinsically long-lived and can maintain serum antibody titers for extended periods of time without requiring significant replenishment from memory B-cells. - [Swain 2021](http://cshperspectives.cshlp.org/content/13/11/a038182) examined mechanisms underlying CD4 T- and B-cell effector responses and memory after influenza virus infection and found that CD4 T-cells require strong initial and extended signals from antigen and pathogen recognition to drive memory and specialized CD4 effectors (such as T follicular helper cell generation). |
| **Milestone 2.2.c**: *Identify distinctions between immune responses to influenza infection and vaccination in different age-groups and birth-year cohorts.*  IN PROGRESS   - [Ugale 2023](https://www.ncbi.nlm.nih.gov/pmc/articles/PMC10590771/) examined influenza infection-related changes in immune cell phenotypes in younger and older individuals. Younger infected individuals had decreased B- and NK-cell frequencies and more effector-like T-cell phenotypes compared with older individuals. - [Ertesvåg 2022](https://pubmed.ncbi.nlm.nih.gov/35750781/) investigated the breadth and durability of influenza A/H3N2-specific HAI Abs after LAIV in children and after IIV or infection in adults; findings suggested that early A/H3N2 exposure and frequent seasonal vaccination could increase the breadth and seropositivity of Ab responses, which may improve vaccine protection against future viruses. - [Shapiro 2021](https://pubmed.ncbi.nlm.nih.gov/34887436/) conducted a multi-season, longitudinal study of older adults over 75 years of age to examine the role of host factors, including age and sex, in determining the effect of repeated vaccination and levels of pre-existing humoral immunity to influenza. Results showed that pre-vaccination titers, rather than host factors or repeated vaccination, strongly predicted post-vaccination Ab titer outcomes. |
| **Milestone 2.2.d**: *Characterize human immune responses to influenza vaccines in diverse populations, according to a range of host factors, including age, sex, pregnancy, obesity, presence of coinfections or other comorbidities, concurrent use of immunotherapies, geographic region, and socioeconomic factors.*  IN PROGRESS   - [Honce 2024](https://pubmed.ncbi.nlm.nih.gov/38637722/) used a mouse model of diet-induced obesity to assess the systemic and specific effects of diet on influenza vaccine immunogenicity; results demonstrated that (1) the systemic meta-inflammation generated by high-fat diet exposure limited T-cell maturation to the memory compartment at the time of vaccination and (2) that the metabolic dysfunction of T-cells was reversed if weight loss occurred 4 weeks before vaccination, restoring a functional recall response. While this study involved mice, the model can be used to inform studies in humans of the effects of diet on influenza immunogenicity. - [King 2024](https://pubmed.ncbi.nlm.nih.gov/39271784/) analyzed data from the US influenza vaccine effectiveness ([Flu VE](https://www.cdc.gov/flu-vaccines-work/php/vaccine-effectiveness/us-flu-ve-network.html)) network over seven seasons to evaluate the relationship between obesity and influenza VE in the outpatient setting and found that elevated BMI was not associated with reduced VE against laboratory-confirmed, outpatient influenza illness. - [Tadount 2024](https://academic.oup.com/ofid/article/11/5/ofae222/7657737) conducted meta-analyses using data from phase 3 RCTs to assess sex differences in the immunogenicity and efficacy of influenza vaccines in healthy adults. Results suggested a higher immunogenicity and VE in females compared to males in older adults. - [Abd Alhadi 2023](https://www.ncbi.nlm.nih.gov/pmc/articles/PMC10269616/) analyzed influenza-specific IgG and IgA Ab repertoires among individuals with healthy weights and obesity prior to and 30 days after vaccination with TIV and found that obesity may impair immune history and cannot be overcome by seasonal vaccination, especially in younger individuals with decreased lifetime exposure to infections and seasonal vaccines. - [Sánchez-de Prada 2023](https://pubmed.ncbi.nlm.nih.gov/37313413/) examined the induction of HA stalk-specific antibodies after seasonal influenza vaccination, considering the age of the cohorts; found that seasonal influenza vaccines can induce cross-reactive anti-stalk Abs against group 1 and group 2 HAs, but that low responses were observed in older groups, highlighting the impact of immunosenescence in adequate humoral immune responses.  - [Zhang 2023](https://pubmed.ncbi.nlm.nih.gov/37383182/) assessed immune responses to IIV at cellular and humoral levels in a cohort of immunocompromised hematopoietic stem cell transplant (HSCT) recipients, at least 1 year post-transplantation, and found that IIV significantly increased HAI titers in HSCT recipients, similar to healthy controls. - [Chou 2022](https://pubmed.ncbi.nlm.nih.gov/35996998/) used a multi-omics approach to identify age-related metabolomic signatures associated with influenza vaccine responses. - [Fourati 2022](https://pubmed.ncbi.nlm.nih.gov/36316476/) identified a common pre-vaccination peripheral blood transcriptional signature is predictive of antibody responses across 13 different vaccines, including live and inactivated influenza vaccine. Results demonstrated that wide variations in the transcriptional state of the immune system can be a key determinant of responsiveness to vaccination. |
| ***Strategic Goal 2.3:*** Improve understanding of aspects of the B-cell immune response to influenza infection and vaccination that are important for developing better vaccines and optimal strategies for vaccination, particularly in the context of partial preexisting immunity from continual exposure to influenza viruses. |
| **Milestone 2.3.a**: *Determine the mechanisms underlying the production of durable, broadly protective B-cell immunity driven by long-lived plasma cells in the bone marrow.*  IN PROGRESS   - [Matz 2024](https://www.biorxiv.org/content/10.1101/2024.10.10.617255v1) assessed B-cell responses in peripheral blood and draining lymph nodes in vaccinees receiving licensed inactivated (Fluarix) or investigational mRNA-based quadrivalent influenza (Moderna mRNA-1010) vaccine, characterizing the dynamics of germinal center (GC) reactions that enable the generation of broad and durable antibody responses. - [McIntire 2024](https://rupress.org/jem/article-abstract/221/8/e20240668/276825/Maturation-of-germinal-center-B-cells-after) examined the maturation of B cells within germinal centers (GC) that affect breadth and durability of B-cell responses to influenza vaccination. Using fine-needle aspiration of draining lymph nodes to longitudinally track antigen-specific GC B-cell responses to seasonal influenza vaccination, the study found that antigen-specific GC B cells persisted for at least 13 weeks after vaccination in two of seven healthy adult volunteers 13 weeks after vaccination and, in some cases, clones that developed in the persistent GC exhibited increased affinity to vaccine antigens and could bind and neutralize diverse influenza viruses.  - [Motsoeneng 2024](https://pubmed.ncbi.nlm.nih.gov/38743692/) measured H1 stalk-specific antibody-dependent cellular phagocytosis (ADCP), complement deposition (ADCD) and cellular cytotoxicity (ADCC) in plasma samples from pregnant women enrolled in a randomized trial in Soweto, South Africa, to evaluate mechanisms through which vaccine-elicited HA stalk-specific antibodies confer protection against influenza illnesses. Results showed that H1 stalk-specific ADCP and ADCD, but not ADCC potential, were boosted by seasonal TIV and that H1 stalk-specific ADCD correlated with protection against influenza illness.  - [Edgar 2023](https://pubmed.ncbi.nlm.nih.gov/37871218/) examined the mechanisms by which passive transfer of broadly reactive stalk-specific IgG Abs, induced in humans in a phase 1 clinical trial using a chimeric HA (cHA) vaccine candidate, protect against lethal influenza virus challenge in humanized mice. Results showed that cHA vaccination-induced IgG Abs fully protected FcγR humanized mice but not FcγR-deficient mice, suggesting a major role for FcγR pathways in the protective function of vaccine-elicited IgG antibodies. - [Nellore 2023](https://pubmed.ncbi.nlm.nih.gov/36958335/) showed that intramuscular influenza vaccination elicits HA-specific memory B cells that differed in expression of surface marker FcRL5 and transcriptional factor T-bet. The T-bet-expressing effector memory subset response correlated with long-lived Ab responses to influenza vaccination. - [Piepenbrink 2023](https://pubmed.ncbi.nlm.nih.gov/37318331/) demonstrated that IIV vaccination stimulates H3N2-specific mAbs in humans that are broad and potent in their neutralization of virus in vitro, provide protection from H3N2 infection in a mouse model, and persist in long-lived Ab-producing plasma cells in the bone marrow. - [Yang 2023](https://www.ncbi.nlm.nih.gov/pmc/articles/PMC10541104/) characterized the breadth and diversity of the polyclonal serum Ab response elicited after H2 vaccination, showing that previous H2 exposure results in higher responses to the variable HA head domain while initial responses in H2-naïve participants are dominated by Abs targeting conserved epitopes. - [Burton 2022](https://pubmed.ncbi.nlm.nih.gov/36351385/) examined how aging impacts the memory B-cell response, using single-cell RNA sequencing and flow cytometry of influenza-vaccine-specific B-cells to delineate changes in B-cell memory generation, antibody mutation, and their subsequent selection in older persons. Results indicated age-related skewing in the memory B-cell compartment 6 weeks after vaccination. - [Moin 2022](https://pubmed.ncbi.nlm.nih.gov/36356572/) examined the role of the immunosubdominant yet conserved HA stem as a target for bnAbs and showed that the co-immunization of two HA stem immunogens derived from group 1 and 2 IAVs (based on a ferritin nanoparticle platform) elicits cross-group protective immunity and nAb responses in mice, ferrets, and NHPs. |
| **Milestone 2.3.b**: *Identify the factors that determine immunodominance hierarchies of B-cell responses to surface glycoproteins and identify methods to target subdominant, functionally conserved antigenic sites.*  IN PROGRESS   - [Mantus 2025](https://pubmed.ncbi.nlm.nih.gov/39742506/) characterized the stem-specific repertoire of individuals vaccinated with one of three group 2 influenza subtypes (H3, H7, or H10), using epitope mapping to identify two complementary epitope supersites on the group 2 HA stem: a central epitope that was broadly cross-reactive and a lower epitope with narrower breadth but higher potency against H3 subtypes. These results suggest that vaccine strategies that target both of these stem epitopes would elicit broader and more potent immune protection against seasonal and pandemic influenza viruses. - [Ataca 2024](https://pubmed.ncbi.nlm.nih.gov/39580804/) used transgenic mice that reproduce the human IGHV1-69*01 germline-encoded Ab response to the conserved stem epitope on group 1 HA to show that this germline-endowed response can be overridden by a subdominant yet cross-group reactive public Ab response. The results can inform the design of vaccines intended to engage an Ig repertoire with broader cross-reactivity to IAVs. - [Yang 2024](https://pubmed.ncbi.nlm.nih.gov/38717904/) performed a comprehensive structural and biochemical characterization of immune memory on the breadth and diversity of the polyclonal serum Ab response elicited following vaccination with a ferritin nanoparticle vaccine displaying H2 HA in H2-naive and H2-exposed adults. They found that previous H2 exposure results in higher responses to the variable HA head domain. In contrast, initial responses in H2-naive participants are dominated by Abs targeting conserved epitopes. - [Martínez 2024](https://pubmed.ncbi.nlm.nih.gov/38627145/) examined the immunodominance hierarchy of the classical antigenic sites of the H1 protein, based on an analysis of HI titers in 39 human serum samples from the Stop Flu NYU cohort. Results indicated the immunodominance profile displayed by each person is strongly associated with the level of HI titers against the A/Michigan/45/2015 strain and that although biological sex does not appear to influence the hierarchy of the antigenic sites, age seems to play a role in the probability of displaying a specific immunodominance profile. - [Frey 2023](https://pubmed.ncbi.nlm.nih.gov/36689343/) demonstrated in a murine model that nanoparticles presenting HA in an inverted orientation generate tenfold higher anti-stalk antibody titers after a prime immunization and fivefold higher anti-stalk titers after a boost than nanoparticles displaying HA in its regular orientation. - [Puente-Massaguer 2023](https://pubmed.ncbi.nlm.nih.gov/37703367/) identified a method to direct humoral immune responses in a mouse model to conserved HA regions (e.g., stalk, trimer interface) and to the N2 NA through the use of sequential vaccination with cHA vaccine constructs. - [Rijnink 2023](https://pubmed.ncbi.nlm.nih.gov/37916834/) investigated the role of non-neutralizing Abs that target conserved influenza virus proteins in protecting against IAV-induced morbidity and mortality. They identified six human mAbs isolated from two H3N2-infected donors that showed robust binding against the conserved internal NP and M1 of IAV strains and passively transferred these mAbs to mice. Results suggested that human NP and M1 antibodies that are elicited following IAV infection/vaccination do not protect from substantial weight loss in the mouse model and imply that protection afforded targeting these antigens following vaccination/infection is most likely the result of cellular-based immunity. - [Van Reeth 2023](https://doi.org/10.1038/s41467-023-43339-3) used a swine model to induce a pan-H1N1 Ab response by prime-boost immunization with whole inactivated, adjuvanted vaccines based on distinct H1 swine IAV lineages and measured Abs against the HA head, HA stalk, NA, and known H1 antigenic sites. Results showed that three sequential administrations induced detectable nAbs against 88% of a diverse panel of swine and human H1 virus strains, a strategy that outperformed any two-dose regimen or three doses of homologous monovalent or trivalent vaccine, suggesting that three-dose heterologous prime-boost vaccination enhances reactivity with conserved epitopes in the HA head. - [Caradonna 2022](https://pubmed.ncbi.nlm.nih.gov/36351401/) used epitope enrichment to alter immunodominance patterns to favor responses targeting conserved, broadly protective HA epitopes, preferentially inducing B-cell responses toward conserved epitopes. - [Sicca 2022](https://www.frontiersin.org/journals/immunology/articles/10.3389/fimmu.2022.987984/full) evaluated preexisting immunity against IAV by determining levels of nAbs and binding Abs to five strains of IAV in three age cohorts (young, adult, and elderly) at two time-points five years apart. Results demonstrated that in each age cohort, the highest nAb titers were seen for a virus strain that circulated early in life but the highest increase in titer was found for the most recent virus strains. In contrast, the highest virus-binding (IgG) titers were seen against recent virus strains but the biggest increase in titer occurred against older strains; and significant increases in nAb titers against a newly encountered virus strain were observed in all age cohorts demonstrating that pre-existing immunity did not hamper Ab induction. |
| **Milestone 2.3.c**: *Define the B-cell repertoire following infection and vaccination to clarify differences between the responses to natural infection compared with vaccination, which are relevant to inform broadly protective vaccination strategies.*  IN PROGRESS     - [León 2025](https://pubmed.ncbi.nlm.nih.gov/39912630/) used negative stain electron microscopy polyclonal epitope mapping to structurally characterize IgG Ab responses to HA in individuals vaccinated with QIV or infected with IAVs during the 2018-2019 flu season and assess the prevalence of central stem-targeting Abs. Results identified IgGs targeting highly conserved regions (including stem and anchor) on H1 and H3 HAs and established a baseline for assessing polyclonal Ab responses in vaccination and infection. - [Reis 2024](https://pubmed.ncbi.nlm.nih.gov/39635527/) assessed the phenotypic and functional profile of H1N1 HA-specific B-cell response among participants who presented distinct immune response patterns prior to and after vaccination with the split-inactivated quadrivalent seasonal influenza virus vaccine (Fluzone, Sanofi). - [Jia 2024](https://pubmed.ncbi.nlm.nih.gov/38615070/) used serum from a RCT of seasonal TIV in children (NCT00792051) conducted at the onset of the 2009 H1N1 pandemic and monitored for infection with pH1N1. Results suggested that seasonal vaccination can have benefits against pandemic influenza viruses, and some children already have broadly reactive Abs with Fc potential without vaccination. - [Boudreau 2023](https://pubmed.ncbi.nlm.nih.gov/36921600/) applied a systems approach to profile HA- and NA-specific humoral signatures that track with the evolution of broad immunity in a cohort of vaccinated individuals and validate these findings in a second longitudinal cohort. Multivariate analysis revealed the presence of a unique pre-existing Fc-gamma-receptor-binding Ab profile in individuals that evolved broadly reactive HAI and suggesting that preexisting Fc-gamma-R2B binding Abs are a key correlate of the evolution of broadly protective influenza-specific Abs. - [Einav 2023](https://pubmed.ncbi.nlm.nih.gov/38177625/) developed a form of antigenic cartography (a neutralization landscape) that visualizes and quantifies Ab-virus interactions for Abs targeting the influenza HA stem, as a tool to analyze how an individual’s Ab repertoire evolves after vaccination or infection. |
| ***Strategic Goal 2.4:*** Determine the impact of prior influenza virus infection or vaccination on future immune responses to influenza viruses or vaccines. |
| **Milestone 2.4.a***:* *Establish ongoing longitudinal clinical studies to follow cohorts of different age-groups in various geographic locations to enable characterization of immune responses to naturally occurring influenza infection and vaccination over time.*  ACCOMPLISHED   - [NCT05518500](https://clinicaltrials.gov/study/NCT05518500) will prospectively profile immune responses to seasonal influenza vaccine in a cohort of 75 older adults over three annual influenza seasons to identify mechanisms that lead to a loss of response to the vaccines over time.  - [NCT05436184](https://classic.clinicaltrials.gov/show/NCT05436184) (IMPRINT: Immunological memory to prior influenza over time): NIAID- and Open Philanthropy-funded Infant Immunome and Influenza Cohort establishes a long-term, prospective observational cohorts in the US and Mexico to examine immune responses to the infants’ initial influenza exposure (vaccine or infection) and subsequent influenza exposures. - [DIVINCI](https://www.stjude.org/research/global-impact/divinci-flu-research.html) (Dissection of Influenza Vaccination and Infection for Childhood Immunity) establishes a consortium of birth cohort studies in Nicaragua, New Zealand and the US to evaluate mechanisms of imprinting, determine what constitutes protective immunity in early and subsequent influenza infections, including the effects of vaccination, and identify B- and T-cell correlates of protection. - [NCT05108818](https://clinicaltrials.gov/study/NCT05108818), a clinical trial led by the University of Pennsylvania, establishes a NIAID-sponsored long-term cohort study designed to measure influenza virus cellular and humoral immune responses in individuals of different birth years before and after influenza vaccination. |
| *High-Priority Milestone*  **Milestone 2.4.b***:* *Determine through birth-year cohort or clinical studies how repeated influenza vaccinations affect immune responses to subsequent influenza vaccinations, including immune responses to HA, NA, and other antigens.*  IN PROGRESS     - [Fox 2025](https://pubmed.ncbi.nlm.nih.gov/40178253/) conducted a retrospective serologic analysis of clinical samples collected during the PIVOT (Potent Influenza Vaccination strategies in Older adults—randomized immunogenicity) Trial in Hong Kong comparing Ab responses following adjuvanted, high-dose, recombinant HA, or standard dose seasonal influenza vaccine administration annually, or not at all, for 5 years. Results suggested that adjuvanted and recombinant HA vaccines may improve breadth of protection against H3N2 influenza viruses but may not overcome attenuating effects of repeated vaccination. - [Cowling 2024](https://pubmed.ncbi.nlm.nih.gov/39041887/) conducted a randomized clinical trial of repeat influenza vaccination with Flublok (Sanofi) in Hong Kong as part of the DRIVE study; preliminary results from the first 2 years of the study found that repeat and first-time vaccinees had similar postvaccination geometric mean titers to all four seasonal influenza vaccine strains, indicative of similar levels of clinical protection. - [Shannon 2024](https://pubmed.ncbi.nlm.nih.gov/39269455/) examined how the CD4 T-cell response to IIV is established and develops throughout early childhood by quantifying influenza-specific CD4 T-cell responses following IIV over two influenza seasons in 47 vaccinated children who had no documented history of natural influenza infection during the study. Results showed that IIV elicits a CD4 T-cell response to H3 and HAB, with increases in the magnitude of the CD4 T-cell response and changes in cellular functionality throughout childhood. - [Zhang 2024](https://pubmed.ncbi.nlm.nih.gov/38884419/), in a prospective observational cohort study in China, examined Ab changes and B-cell receptor repertoire characteristics among 100 subjects singly or repeatedly immunized with influenza vaccines including 3C.2a1 or 3C.3a1 A(H3N2) during the 2018–2019 and 2019–2020 influenza seasons. They found that vaccination elicited cross‐reactive Ab responses against future emerging strains and observed broader nAbs to A(H3N2) viruses and more diverse B-cell repertoires in the repeated vaccination group. - [Sung 2023](https://pubmed.ncbi.nlm.nih.gov/37624957/) analyzed associations between host factors and antibody responses in a repeated vaccination setting, finding disparate vaccine-elicited immune responses in adults when they were repeatedly vaccinated for at least two seasons, such as interactive effects between age and BMI on overall immune responses and between sex at birth and BMI in the adult age group. - [Jones-Gray 2022](https://www.thelancet.com/journals/lanres/article/PIIS2213-2600(22)00266-1/fulltext) found through a meta-analysis of 41 published studies that vaccination in two consecutive years provides better protection than no vaccination, even though vaccination in the previous year attenuates vaccine effectiveness*.* - [Hinojosa 2021](https://pubmed.ncbi.nlm.nih.gov/33090202/) evaluated effects of Ab landscapes on vaccine responses using sera collected from children over two influenza seasons and found that A(H3N2) Ab landscapes in children were largely determined by age-related immune priming, rather than recent vaccination or infection. - [NCT05110911](https://clinicaltrials.gov/study/NCT05110911): NIAID award R01AI141534 to the University of Melbourne to investigate the long-term consequences of repeated annual influenza vaccination among healthcare workers (not a birth-year cohort study). |
| *High-Priority Milestone*  **Milestone 2.4.c***:* *Determine how the initial encounter with an influenza virus or vaccine (i.e., immune imprinting) affects B- and T-cell responses, including immunologic responses to subsequent influenza virus infection and/or vaccination.*  IN PROGRESS   - [Matz 2025](https://pubmed.ncbi.nlm.nih.gov/40272481/) examines the available evidence that repeated annual influenza virus vaccination may have effects on future vaccine responses and synthesizes the available data with studies that may indicate potential immunological mechanisms underlying these effects; the goal is to determine whether these mechanisms can be redirected to improve the efficacy of seasonal influenza vaccines. - [Spangler 2025](https://pubmed.ncbi.nlm.nih.gov/40023164/) examined the impact on influenza vaccine responses of memory B cells established during childhood influenza exposure by evaluating H2-specific memory B cells in clinical trial participants born before 1968 (exposed to H2N2) and after 1968 (naive to H2N2) vaccinated with an H2 HA DNA plasmid and/or an H2 ferritin nanoparticle vaccine. Results showed that pre-existing and newly generated memory B cells in the two cohorts differed in neutralizing potency, isotype usage, cross-reactivity, epitope targeting, and phenotype, suggesting that pre-existing immunity established early in life continues to affect vaccine responses half a century later. - [Mallajosyula 2024](https://pubmed.ncbi.nlm.nih.gov/39700292/) used mice and human tonsil organoids to examine vaccine designs that can overcome subtype bias (immune imprinting). Results showed that covalent coupling of heterologous HA antigens can limit Ab subtype bias by enhancing the recruitment of T cell help by strain-specific B cells through the presentation of a broader array of HA peptides, thereby improving VE. - [NCT05436184](https://classic.clinicaltrials.gov/show/NCT05436184) (IMPRINT: Immunological memory to prior influenza over time): NIAID- and Open Philanthropy-funded Infant Immunome and Influenza Cohort establishes a long-term, prospective observational cohorts in the US and Mexico to examine immune responses to the infants’ initial influenza exposure (vaccine or infection) and subsequent influenza exposures. - [DIVINCI](https://www.stjude.org/research/global-impact/divinci-flu-research.html) (Dissection of Influenza Vaccination and Infection for Childhood Immunity) establishes a consortium of birth cohort studies in Nicaragua, New Zealand and the USA to evaluate mechanisms of imprinting, determine what constitutes protective immunity in early and subsequent influenza infections, including the effects of vaccination, and identify B- and T-cell correlates of protection. - [Carlock 2024](https://pubmed.ncbi.nlm.nih.gov/38917104/) examined pre-existing immune responses to previous influenza virus exposures as a factor influencing host responses to seasonal influenza vaccination among participants 10 to 86 years old who were vaccinated with split-inactivated influenza vaccine over six consecutive influenza seasons. Results showed that the magnitude and breadth of Ab responses were modulated by age, vaccination history in the past 1 to 2 years, and antigen dose. - [Edler 2024](https://pubmed.ncbi.nlm.nih.gov/38890491/) evaluated the effects influenza exposures early in life on future susceptibility to influenza infections by analyzing HI titers in 1,451 cross-sectional samples collected between 1992 and 2020, from individuals born between 1917 and 2008, against IBV isolates from 1940 to 2021. Results showed that immunological biases are conferred by early life IBV infection and result in lineage-specific cross-reactivity of a birth cohort towards future IBV isolates. These results lead to differential estimates of susceptibility between birth cohorts towards infection by IBV lineages and can predict lineage-specific birth-cohort distributions of observed medically attended IBV infections. - [Guiomar 2024](https://pubmed.ncbi.nlm.nih.gov/39591121/) evaluated the impact of repeated TIV uptake on vaccine-derived immunity against influenza in a cohort of HCWs. Results showed that previous vaccination can influence the immune response without substantially compromising the immunogenicity of annual influenza vaccination. - [Liu 2024](https://pubmed.ncbi.nlm.nih.gov/38177116/) investigated whether repeat vaccination with non-egg-based vaccines can overcome the effect of prior repeat vaccination with egg-based vaccines. Results showed that RIV4 induced more robust HI and MN Ab responses than ccIIV4 and egg-based IIV4 against multiple vaccine strains including cell-propagated A(H3N2) and that repeat vaccination with non-egg-based vaccines could overcome pre-existing egg/cell titer differences in nAb levels by re-directing vaccine-induced nAb responses away from egg-adapted epitopes, resulting in higher Ab responses to circulating cell-grown viruses, even with frequent prior vaccination with egg-based influenza vaccines in healthcare workers. - [Lu 2024](https://pubmed.ncbi.nlm.nih.gov/37981659/) evaluated whether preexisting HAI Abs targeting the K163 epitope on the HA affected Ab responses following vaccination with A/California/07/2009-like A(H1N1)pdm09 influenza viruses in humans. Results showed dose-dependent suppression of Ab responses by preexisting Abs: at high K163 Ab levels, all HAI Ab responses were suppressed, but at moderate K163 Ab levels, only epitope-specific K163 Abs were suppressed. Novel HAI antibody responses targeting the non-K163 epitopes were induced by vaccination. - [Einav 2023](https://pubmed.ncbi.nlm.nih.gov/36851590/) used a computational tool to track progression of HAI within ferret antisera elicited by repeated influenza A/H3 infections and analyzed the influence of prior exposures on the de novo Ab response to evolved viruses. Results indicate that repeat infections can induce a bnAb signature despite immune imprinting. - [Lobby 2023](https://pubmed.ncbi.nlm.nih.gov/37982700/) assessed the impact of pre-existing humoral and cellular immunity on the ability of LAIV to generate de novo Ag-specific CD8 T_RM_ in the respiratory tract, demonstrating that pre-existing humoral and cellular immunity can limit the effectiveness of LAIVs. - [Tsang 2023](https://pubmed.ncbi.nlm.nih.gov/36637115/) analyzed population-level data from Hong Kong to determine the degree of protection from imprinting and estimated that imprinting protections were weaker than estimates in western countries, suggesting the existence of multiple factors affecting the strength of imprinting protections, such as geographic or season-specific effects. - [Auladell 2022](https://www.nature.com/articles/s41591-022-01690-w) reported on a longitudinal household cohort study of the effect of prior H3N2 infection on HAI responses induced by seasonal vaccine in a vaccine-naïve population in Vietnam. Results showed that recent H3N2 infection can overcome early life HA imprinting, leading to updated responses to more recent strains and immune memory elicited by influenza infection can induce broader subtype-specific protection. - [Brouwer 2022](https://journals.plos.org/plospathogens/article?id=10.1371/journal.ppat.1010317) found that Ab responses to a virus depends on one’s age when a related virus from the same antigenic cluster first circulated, not when that specific virus circulated. In addition, young children may have Abs that cross-react with virus strains that have not yet circulated, possibly indicating that their immune systems are creating a wide array of Abs, which could inform possible mechanisms of antigenic seniority. - [Fox 2022](https://pubmed.ncbi.nlm.nih.gov/35336877/) combined data from two different study populations (vaccine-naïve adults who had participated in influenza surveillance for nine years in Viet Nam and HCWs in Australia with varying prior exposures to vaccination) to directly compare Ab titers against 35 A(H3N2) viruses spanning 1968–2018. Results indicate that recall of existing memory can enhance Ab titers induced by IIVs but may concurrently limit the generation of memory against variant epitopes of vaccines, accounting for the detrimental effect of repeated vaccination. - [Moritzky 2022](https://pubmed.ncbi.nlm.nih.gov/35199825/) examined the impact of accumulated immunity from annual influenza vaccination to split, subunit, and recombinant protein-based influenza vaccines in a cohort of over 230 subjects. Results indicate that the accumulated immunity specific to influenza A H1 and H3 proteins is associated with diminished future responses. - [Wraith 2022](https://pubmed.ncbi.nlm.nih.gov/35246548/) examined the effects of influenza infection on subsequent infection with the same influenza virus subtype/lineage across multiple seasons in a large pediatric cohort in Nicaragua and found that protection wanes as time or antigenic distance increases. - [Yegorov 2022](https://doi.org/10.1016/j.xcrm.2022.100509) evaluated the impact of repeated influenza vaccination across three seasons and the vaccine-elicited induction of group 1 influenza virus HA stalk broadly neutralizing Abs in children (median age 9 years), comparing the impact of IIV vs LAIV. The study found that repeated vaccination results in significant boosting of a durable bnAb response and that IIV and LAIV formulations elicit comparable boosting of serological bnAb titers (anti-stalk IgG and IgA). - [Shapiro 2021](https://pubmed.ncbi.nlm.nih.gov/34887436/) evaluated pre- and post-vaccination strain-specific HAI titers in adults over 75 years of age who received a high-dose influenza vaccine in at least four of six seasons to estimate the impact of repeat vaccination on Ab responses to HD vaccination and its dependence on age, sex, frailty, BMI, and pre-existing immunity. Pre-vaccination titers, rather than host factors and repeated vaccination, were significantly associated with post-vaccination HAI titer outcomes, and displayed an age-by-sex interaction. |
| ***Strategic Goal 2.5:*** Clarify the role of T cells in generating or supporting protective immunity to influenza virus infection and/or vaccination. |
| **Milestone 2.5.a**: *Identify major influenza epitopes recognized by CD4 and CD8 T cells for a comprehensive range of human leukocyte antigen (HLA) types and define the epitopes that elicit broadly protective T-cell immunity.*  IN PROGRESS   - [Grant 2024](https://pubmed.ncbi.nlm.nih.gov/39584190/) assessed the conservation of known CD8 T-cell epitopes in the recently circulating H5N1 viruses to determine the potential for pre-existing immunity to H5N1 clade 2.3.4.4b viruses in humans. Results showed that > 64% of the CD8 T-cell epitopes are highly conserved in the H5N1 viruses, suggesting the potential for T-cell cross-recognition against the H5N1 viruses providing some protection in humans. - [Menon 2024](https://pubmed.ncbi.nlm.nih.gov/38684663/) identified nine IBV CD8 T-cell epitopes restricted by prominent HLA-B*07:02, HLA-B*08:01, and HLA-B*35:01 alleles, increasing the number of known IBV CD8 T-cell epitopes by 50% for potential application in the design of cross-reactive vaccines to elicit CD8 T-cell immunity against IBV. - [van de Sandt 2023](https://pubmed.ncbi.nlm.nih.gov/37749325/) defined epitope-specific CD8 T-cell immunity across the human lifespan in newborns, children, adults and older adults. The authors performed ex vivo phenotypic, transcriptome, functional, and TCRαβ repertoire analyses of A2/M1_58_-specific CD8 T cells, and identified age-related TCR repertoire shifts within older epitope-specific CD8 T cells, stemming from newborn/child-like molecular signatures detected in older adults. |
| **Milestone 2.5.b**: *Determine CD4 or CD8 T-cell effector functions that are most needed to enhance protective immunity or to modulate disease severity and determine whether T cells require localization in lymph nodes or mucosal tissue to be protective, and characterize the drivers and markers of T-cell homing to the respiratory mucosa.*  IN PROGRESS     - [Schattgen 2024](https://pubmed.ncbi.nlm.nih.gov/39164477/) profiled blood and draining lymph node samples from human volunteers for over 2 years after two influenza vaccines were administered 1 year apart to define the evolution of the CD4 TFH cell response. They identified several influenza-specific TFH cell clonal lineages, including multiple responses targeting internal influenza virus proteins, and found that each TFH cell state was attainable within a clonal lineage, indicating that human TFH cells form a durable and dynamic multi-tissue network. - [Lobby 2022](https://pubmed.ncbi.nlm.nih.gov/36162870/) examined the mechanisms driving enhancement of T_RM_ cells in the respiratory tract after immunization with replication-deficient Ad vectors vaccines in a murine model. - [Zheng 2022](https://pubmed.ncbi.nlm.nih.gov/36426954/) investigated how pre-existing Ab immunity to influenza virus established from prior immunizations affects the development of CD8 T-cell responses evoked after vaccination with an LAIV. Results showed that pre-existing influenza-specific Abs directed against the vaccine backbone attenuate the size and quality of the vaccine-induced CD8 T-cell response, but that increasing the vaccine dose can overcome this impediment, resulting in improved vaccine-induced circulating and tissue-resident memory CD8 T-cell responses, which were protective against heterologous influenza challenge. - [Paterson 2021](https://pubmed.ncbi.nlm.nih.gov/34256007/) investigated the kinetics, phenotypes, and function of influenza virus-specific CD8 T_RM_ cells in the lower airway. Results indicated that CD8 T_RM_ cells in the human lung display innate-like gene and protein expression, demonstrating blurred divisions between innate and adaptive immunity. - [Thapa 2021](https://pubmed.ncbi.nlm.nih.gov/34890254/) used a mouse co-transfer model to examine how infant T cells generate greater numbers of lung-homing effector cells in response to influenza infection compared with adult T cells in the same host, due to augmented T-cell receptor–mediated signaling. |
| **Milestone 2.5.c**: *Define the role of heterologous infections (e.g., sequential infection with different influenza A virus strains) on the establishment and maintenance of CD4 and CD8 T cells.*  IN PROGRESS   - [Sircy 2024](https://pubmed.ncbi.nlm.nih.gov/39283916/) used a heterologous infection or immunization priming strategy to seed an antigen-specific memory CD4 T-cell pool prior to influenza infection in mice to evaluate the effect of recalled memory T follicular helper cells in increased help to influenza-specific primary B cells and enhanced generation of neutralizing antibodies. Results showed that heterologous priming induced an increase in both CD4 T cells and B cells early following influenza infection, suggesting successful target enhancement of the germinal center, but did not result in an increase in influenza-specific antiviral antibodies. - [Bull 2023](https://pubmed.ncbi.nlm.nih.gov/36401824/) determined the early cytokine impact of HA mismatch during infection of mice vaccinated with a T-cell activating next-generation influenza vaccine, showing the negative effect of exuberant cytokine production and subsequent mild morbidity in sublethal seasonal influenza infection, outcomes that were outweighed by the essential benefit of survival against highly lethal AIVs and higher doses of seasonal influenza viruses. - [Currenti 2023](https://pubmed.ncbi.nlm.nih.gov/37063867/) examined the TCR repertoire and transcriptional profile of vaccine-induced circulating T follicular helper (cTfh) cells in individuals who received sequential seasonal influenza vaccines and identified the expansion and transcriptomic profile of vaccine-induced cTfh cells important for B-cell help. |
| ***Strategic Goal 2.6:*** Improve understanding of the role of mucosal immunity in protecting against influenza. |
| *High-Priority Milestone*  **Milestone 2.6.a***:* *Characterize the role of mucosal immunity (including antibodies and T cells) in protecting against influenza virus infection, disease, and transmission.*  IN PROGRESS   - [Gailleton 2025](https://pubmed.ncbi.nlm.nih.gov/40112112/) examined the origin and dynamics of B cell responses to influenza virus in the nasal mucosa using an upper respiratory tract-restricted infection method in a mouse model that mimics infection dynamics in humans. Results demonstrated that intranasal infection and immunization trigger a specific germinal center B cell response in the nasal turbinate which fuels the local B cell and Ab response. - [NCT06501963](https://clinicaltrials.gov/study/NCT06501963) Revealing Protective Immunity to Influenza Using Systems Immunology (PRISM) was initiated in 2024 to examine why the LAIV given as a nasal spray does not work as well in adults as it does in children. - [Kazer 2024](https://pubmed.ncbi.nlm.nih.gov/38964332/) generated a single-cell RNA-sequencing atlas of the murine nasal mucosa, sampling three regions during primary influenza infection and rechallenge, and showed that influenza infection induces stepwise changes in nasal epithelial and immune subsets. - [Kastenschmidt 2023](https://pubmed.ncbi.nlm.nih.gov/37478854/) used a human tonsil organoid model to examine how antigen-specific B and T cells were activated and participated in adaptive immune responses within the mucosal site by tracking the differentiation and kinetics of the adaptive immune response to influenza vaccine and virus modalities; each antigen format elicited distinct B- and T-cell responses, including differences in magnitude, diversity, phenotype, function, and breadth. - [Mahallawi 2023](https://pubmed.ncbi.nlm.nih.gov/37766886/) used a human NALT model to evaluate local B-cell immunity induced by LAIV. Mucosal antibody responses in NALT to HAs of a number of influenza A and one influenza B viruses were investigated following *in vitro* stimulation of tonsillar cells with LAIV vaccine. Significant Ab responses of IgG, IgA and IgM to the HA of pH1N1 virus were observed in tonsillar cells following LAIV stimulation, suggesting that human NALT tissues are likely to be a major induction site of immune response against influenza following LAIV at the nasopharynx site. - [Thwaites 2023](https://pubmed.ncbi.nlm.nih.gov/38052824/) inoculated 40 healthy young adults with LAIV and performed detailed analyses of immune activation after vaccination. Results indicated the efficacy of LAIV may result from Ab responses confined to the nasal mucosa, which may provide protection not reflected by blood-based studies. - [Rattan 2022](https://pubmed.ncbi.nlm.nih.gov/35215193/) developed a mouse model of intranasal infection with influenza B virus and identified a series of CD4 T-cell peptide specificities to enable in depth analyses of the role of influenza B-specific CD4 T cells elicited by infection and vaccination and to assess the impact of candidate vaccines on the abundance, functionality, and localization of the elicited CD4 T cells. - [Oh 2021](https://www.ncbi.nlm.nih.gov/pmc/articles/PMC8762609/) used a murine model to evaluate the phenotype, residency, and role of IgA-secreting B cells in the lung and found that tissue-resident IgA-secreting B cells are the source of luminal IgA in the lung that confers sterilizing protection against challenge with homologous influenza virus and quicker recovery from heterologous challenge (preclinical study in mice). - [Paterson 2021](https://pubmed.ncbi.nlm.nih.gov/34256007/) used a human challenge model to evaluate human lung CD8 T_RM_ cells finding innate-like gene and protein expression between innate and adaptive immunity. |
| **Milestone 2.6.b**: *Clarify drivers of myeloid and lymphoid cell differentiation and migration to protect the upper and/or lower respiratory airway and determine how this information can be translated into vaccine R&D.*  IN PROGRESS   - [Madissoon 2023](https://pubmed.ncbi.nlm.nih.gov/36543915/) profiled 5 proximal-to-distal locations of healthy human lungs using multi-omic single cell/nuclei and spatial transcriptomics ([lungcellatlas.org](http://lungcellatlas.org/)); identified 80 cell types/states from >190,000 cells including 11 new cell types and discovered new anatomical compartments s, providing a survival niche for IgA plasma cells in the airway submucosal glands. |
| **Milestone 2.6.c**: *Identify how the route of vaccination (e.g., intranasal, oral, sublingual, or other mucosal route) affects the magnitude of mucosal humoral and cellular immunity.*  IN PROGRESS   - [Zhang 2024](https://pubmed.ncbi.nlm.nih.gov/39508450/) examined mechanistic differences between IM and IN delivery of a recombinant adenovirus carrying NP fused with a bifunctional CD40 ligand and found that CD40-targeted nasal delivery improved the magnitude and breadth of protection, including against lethal challenge with a newly isolated HPAI H5N1 strain. - [Yu 2023](https://pubmed.ncbi.nlm.nih.gov/37044258/) used a mouse model to compare the induction of lung tissue-resident CD8 T cells following vaccination via microneedle array, IM, and ID immunizations. Results indicated stronger lung CD8 T-cell responses against influenza viral infection via microneedle array than by ID or IM immunization. - [Künzli 2022](https://pubmed.ncbi.nlm.nih.gov/36459542/) characterized the abundance, phenotype, and anatomic distribution of mRNA vaccine–elicited antigen-specific CD8 and CD4 memory T cells in a mouse model. Results showed that the routes of mRNA vaccination influenced humoral and cell-mediated immunity and intramuscular prime-boosting established lung T_RM_ that can be further expanded by an additional intranasal immunization. |
| ***Strategic Goal 2.7:*** Develop novel correlates of protection for assessing seasonal influenza vaccines and broadly protective or universal influenza vaccines as part of clinical studies that demonstrate efficacy against a disease endpoint. |
| **Milestone 2.7.a***:* *Develop functional assays that are fit for clinical trial purpose to accurately capture the breadth and range of protective responses other than virus neutralization, such as influenza virus–specific ADCC, antibody-dependent cellular phagocytosis, and complement dependent cytotoxicity.*  IN PROGRESS   - [de Vries 2023](https://www.ncbi.nlm.nih.gov/pmc/articles/PMC9992787/) compiled ADCC data obtained in four separate studies to get a comprehensive overview of the induction of ADCC-mediating Abs after influenza virus infection, or immunization with monovalent, trivalent, or quadrivalent influenza vaccines. - [Meade 2023](https://pubmed.ncbi.nlm.nih.gov/36533948/) used influenza virus protein microarray technology to measure Ab responses (to group 1 and 2 HAs and influenza B HAs) induced by universal influenza vaccine cHA constructs in phase 1 clinical trials, showing that cHA vaccines induce very broad cross-subtype Ab responses. - [Mettelman 2023](https://pubmed.ncbi.nlm.nih.gov/37592015/) defined baseline immune cell subsets correlated with protection against symptomatic influenza independently from or synergistically with humoral responses and investigated the contributions of baseline cellular and humoral immune responses in mediating protective anti-influenza virus immunity. Results demonstrated that the baseline composition of peripheral cells improved the prediction of influenza susceptibility over serology, vaccination, or demographics alone. - [Bernard 2022](https://pubmed.ncbi.nlm.nih.gov/35784305/) (FLUCOP Consortium) validated a harmonized ELLA-NA Inhibition (NI) SOP for N1 influenza antigen (providing a publicly available SOP for ELLA-NI), tested the SOP with NA from IBVs, showed the assay performed consistently with both IAV and IBV antigens, and demonstrated that recombinant NA could be used as a source of antigen in ELLA-NI. - [Chen 2022](https://doi.org/10.1016/j.virol.2022.02.004) developed four novel cell-based assays to assess ADCC antibodies against HA or NA proteins to assess the contribution of ADCC antibody to vaccine immunogenicity. - [Cheung 2022](https://pubmed.ncbi.nlm.nih.gov/36146550/) developed two ELISA-based potency assays for group 1 influenza A viruses using cross-reactive nanobodies. - [Waerlop 2022](https://www.frontiersin.org/articles/10.3389/fimmu.2022.984642) (FLUCOP Consortium) described the harmonization and qualification of the influenza-specific interferon-gamma ELISpot assay to detect and qualify vaccine-induced cellular immune responses. |
| *High-Priority Milestone*  **Milestone 2.7.b***:* *Develop correlates of protection for mucosal immunity based on different vaccine platforms (e.g., live-attenuated influenza virus) and routes of administration (e.g., intranasal).*  IN PROGRESS   - [Bean 2024](https://pubmed.ncbi.nlm.nih.gov/38193710/) conducted a CHIVIM study in vaccinated and unvaccinated healthy volunteers to investigate mucosal and systemic immunity after influenza vaccination and infection. They found that correlations between mucosal IgA and serum IgG against specific antigens were low, whether before or after challenge, suggesting a compartmentalization of immune responses. - [Rattan 2022](https://pubmed.ncbi.nlm.nih.gov/35215193/) developed a mouse model of intranasal infection with influenza B virus to analyze the localization of infection-elicited virus-specific CD4 T cells in secondary lymphoid tissues and lung and examined the distribution of IBV-specific CD4 T cells in lung sub-compartments. - [McIlwain 2021](https://doi.org/10.1016/j.chom.2021.10.009) identified new single and multi-variable cellular correlates of protection following oral vaccination with an Ad5-based influenza vaccine candidate (Vaxart VXA-A1.1). The authors found that XA-A1.1 elicits mucosal homing cell subsets that correlate with protection. |
| *High-Priority Milestone*  **Milestone 2.7.c**: *Conduct side-by-side standardized comparative studies of immune markers, beyond serum HAI titers, as potential correlates of protection in vaccine efficacy/effectiveness studies or in studies that use a controlled human influenza virus infection model.*  IN PROGRESS   - [Waldock 2023](https://pubmed.ncbi.nlm.nih.gov/37143658/) (FLUCOP Consortium) conducted a head-to-head comparison of harmonized HAI and MN protocols to evaluate the relationship between HAI and MN titers, and the impact of assay harmonization and standardization on inter-laboratory variability, and agreement between these methods. |
| *High-Priority Milestone*  **Milestone 2.7.d:** *Develop correlates of protection for influenza vaccine candidates based on antigens other than HA (e.g., NA, NP, HA stem), for vaccines produced using novel platforms (e.g., nanoparticles and nucleic acids), and relevant to a variety of endpoints (e.g., cellular immune responses, severe disease).*  IN PROGRESS   - [Wagoner 2025](https://pubmed.ncbi.nlm.nih.gov/39986275/) used a systems immunology approach combined with a tonsil organoid model to investigate how host and antigen features affect mucosal and lymphoid tissue responses to influenza vaccines. The study identified immune signatures correlated with nAb responses across seven different influenza vaccines and antigens, including elevated T helper (Th)1 signatures associated with Ab responses to inactivated influenza vaccines. - [Hoy 2024](https://pubmed.ncbi.nlm.nih.gov/38723107/) used data from two household influenza virus transmission studies across 3 influenza seasons in Nicaragua to identify the HA stem as an independent correlate of protection against H3N2 infection; results also showed that the role of anti-HA stem responses in infection is dependent on age and influenza exposure history. - [Mettelman 2023](https://pmc.ncbi.nlm.nih.gov/articles/PMC10566627/) evaluated baseline variations in cellular and humoral immune responses among 206 vaccinated or unvaccinated adults in relation to protection from (and increased susceptibility to) symptomatic influenza infection after vaccination. Results showed that protection correlated with diverse and polyfunctional CD4+ and CD8+ T, circulating T follicular helper, T helper type 17, myeloid dendritic and CD16+ natural killer (NK) cell subsets; increased susceptibility correlated with nonspecific inflammatory populations. |

**TOPIC 3: VACCINOLOGY FOR SEASONAL INFLUENZA VACCINES**

| ***Strategic Goal 3.1:*** Promote strategies that shorten the lag time from identification of candidate vaccine viruses through the process of annual vaccine production and release. |
| --- |
| **Milestone 3.1.a:** *Streamline the production of new potency assays to ensure timely release of annual vaccine preparations.*  ACCOMPLISHED   - [Odin 2024](https://pmc.ncbi.nlm.nih.gov/articles/PMC11497102/) used a mammalian cell transfection method to produce recombinant HAs as immunogens for the SRID assay, which measures the potency of inactivated influenza vaccines. Results suggest that this method can be used to prepare a library of reagents for influenza vaccine potency assays. - [Bodle 2023](https://pubmed.ncbi.nlm.nih.gov/37033922/) demonstrated the applicability of the homologous mAb ELISA as a replacement for the SRID assay for HA antigen quantitation and stability assessment. - [Narayan 2023](https://pubmed.ncbi.nlm.nih.gov/37457687/) developed a Surface Plasmon Resonance-based method using influenza subtype or lineage HA-specific mAbs to measure the HA concentration in multivalent influenza vaccines, which eliminates the need for annually produced antisera reagents. - [Qian 2023](https://pubmed.ncbi.nlm.nih.gov/37587402/) developed a streamlined subtype-specific, reference antigen and antisera-free method (CombE-IDMS) for rapidly assaying the potency of influenza vaccines as an alternate to the SRID assay. - [Cheung 2022](https://www.ncbi.nlm.nih.gov/pmc/articles/PMC9503116/) developed two ELISA-based potency assays for group 1 influenza A viruses using cross-reactive nanobodies. |
| **Milestone 3.1.b:** *Provide funding (such as through private-public partnerships) to further develop the most promising egg-independent vaccine production methods.*  IN PROGRESS   - [BARDA Strategic Plan 2022-2026](https://www.medicalcountermeasures.gov/media/38717/barda-strategic-plan-2022-2026.pdf) outlined a plan to improve the manufacturing and fill-finish capacity for seasonal influenza vaccines and to advance the development of vaccines against pandemic influenza through public-private partnerships, as part of BARDA’s role in the [National Influenza Vaccine Modernization Strategy (NIVMS), 2020-2030](https://www.phe.gov/Preparedness/planning/nivms/Pages/default.aspx). |
| **Milestone 3.1.c:** *Continue to conduct studies comparing efficacy, safety, and relative effectiveness of new egg-independent production methods to each other and to egg-based methods.*  IN PROGRESS   - [Askar 2025](https://onlinelibrary.wiley.com/doi/10.1002/rmv.70020) updated a previous systematic review to reassess evidence on the safety and efficacy/effectiveness of newer and/or enhanced influenza vaccines, including mRNA-based vaccines, compared with standard influenza vaccines. Newer data accumulated since 2020 were reassuring regarding safety of the vaccines. Evidence regarding relative VE of the vaccines against lab-confirmed outcomes has only slightly improved. - [Fowlkes 2024](https://pubmed.ncbi.nlm.nih.gov/39395753/) conducted a randomized immunogenicity trial of RIV4 vs. standard-dose IIV4 among frequently and infrequently vaccinated healthcare personnel and found that RIV4 had improved immunogenicity for influenza vaccine strains compared to IIV4. - [Essink 2022](https://pubmed.ncbi.nlm.nih.gov/36214072/) conducted a phase 3 RCT to evaluate the safety and immunogenicity of a cell-based QIV (QIVc) compared with egg-based QIV; QIVc was well tolerated and immune responses were similar to QIV in children 6 to 47 months of age. - [Gouma 2022](https://journals.asm.org/doi/10.1128/jvi.01723-22) used a murine model to directly compare nucleoside-modified mRNA-LNP vaccines expressing wild-type and egg-adapted H3 with a conventional inactivated egg-based influenza vaccine, and found that nucleoside-modified mRNA-LNP-based vaccines can circumvent problems associated with egg adaptations with recent H3N2 viruses. - [Rockman 2022](https://pubmed.ncbi.nlm.nih.gov/36679895/) examined the benefits of cell-based vaccines produced using mammalian (MDCK) or insect (baculovirus recombinant) cell lines to improve seasonal influenza vaccine effectiveness, compared with currently available egg-based production strategies. - [Izurieta 2021](https://pubmed.ncbi.nlm.nih.gov/33211809/) compared the effectiveness of five types of conventional seasonal influenza vaccines (e.g., egg-based, recombinant, cell-based, high-dose, and adjuvanted) administered to Medicare beneficiaries ages 65 years and older. - [Murchu 2021](https://pubmed.ncbi.nlm.nih.gov/35106885/) conducted a systematic review of data on the efficacy and effectiveness of trivalent recombinant HA seasonal influenza vaccines compared with conventional QIV in adults aged 50 or older. |
| ***Strategic Goal 3.2:*** Identify strategies and policies to optimize seasonal influenza vaccines and improve vaccine benefit-risk profiles. |
| **Milestone 3.2.a:** *Identify lessons learned from COVID-19 vaccine development that are applicable to improving seasonal influenza vaccines. Include a summary of the current landscape of next-generation influenza vaccine candidates based on mRNA and other new platform technologies used in COVID-19 vaccines.*  ACCOMPLISHED   - The scope of technologies in the [Universal Influenza Vaccine Technology Landscape](https://ivr.cidrap.umn.edu/universal-influenza-vaccine-technology-landscape) was expanded to include next-generation influenza vaccines, as defined in the [IVR](https://ivr.cidrap.umn.edu/roadmap), and COVID/seasonal influenza combination vaccines in clinical development. - [Bresee 2023](https://pubmed.ncbi.nlm.nih.gov/37872012/): WHO and LSHTM, with funding from US CDC as part of a 5-year cooperative agreement ending in 2024, are engaged in a full value of influenza vaccine assessment (FVIVA) project aimed at identifying critical success factors that would enable the development, approval, and introduction of next-generation influenza vaccines ([WHO FVVA webpage](https://www.who.int/teams/immunization-vaccines-and-biologicals/product-and-delivery-research/full-vaccine-value-assessments)). This project will also include additional lessons learned from the COVID-19 experience. - [NASEM 2022](https://nap.nationalacademies.org/catalog/26282/vaccine-research-and-development-to-advance-pandemic-and-seasonal-influenza-preparedness-and-response) [consensus study report] provided recommendations for basic and translational research, clinical evaluation, manufacturing, and regulatory science for seasonal and pandemic influenza vaccines, based on an expert committee’s review of the rapid development, evaluation, licensing, and deployment of effective COVID-19 vaccines. - [IFPMA 2022](https://www.ifpma.org/resource-centre/applying-lessons-learned-from-covid-19-to-create-a-healthier-safer-more-equitable-world/) summarized lessons learned for vaccine manufacturing during a pandemic (e.g., regarding pathogen surveillance and data sharing, equitable distribution, and pharmaceutical partnerships) to accelerate R&D and manufacturing. |
| *High-Priority Milestone*  **Milestone 3.2.b***:* *Review the development and safety of novel vaccine platforms to identify how best to apply them to develop improved seasonal influenza vaccines.*  IN PROGRESS   - [Arevalo 2022](https://pubmed.ncbi.nlm.nih.gov/36423275/) generated a nucleoside-modified mRNA-LNP vaccine expressing HA antigens from all known influenza virus subtypes and found that this multivalent vaccine elicits diverse Abs that protect mice and ferrets against matched and mismatched viral strains. - Interest in applying novel platforms to seasonal influenza vaccines is evident; 10 mRNA-based next-generation influenza vaccine candidates are now in clinical development (see [Landscape](https://ivr.cidrap.umn.edu/universal-influenza-vaccine-technology-landscape)):   - Phase 3: Moderna mRNA-1010; Pfizer/BioNTech modified mRNA   - Phase 2: CureVac/GSK multivalent modified mRNA; Moderna mRNA-1011/1012; Moderna mRNA-1020/1030; Sanofi mRNA; Pfizer sa-RNA   - Phase 1: Arcturus/CSL sa-RNA (ARCT-2138); CSL Seqirus sa-mRNA(Sq012); DCVC H1 HA mRNA-LNP |
| **Milestone 3.2.c:** *Document that at least two combined COVID-19 and seasonal influenza vaccines are being evaluated in clinical trials.*  ACCOMPLISHED  Seven COVID+Influenza combination vaccine candidates are in active clinical development, as profiled in the [Landscape](https://ivr.cidrap.umn.edu/universal-influenza-vaccine-technology-landscape):   - Novavax CIC nanoparticle ([NCT04961541](https://clinicaltrials.gov/ct2/show/NCT04961541), [NCT05519839](https://clinicaltrials.gov/ct2/show/NCT05519839)) - Moderna mRNA-1083 ([NCT06097273](https://clinicaltrials.gov/study/NCT06097273); [NCT05375838](https://clinicaltrials.gov/ct2/show/NCT05375838), [NCT05827926](https://clinicaltrials.gov/ct2/show/NCT05827926)) - Moderna mRNA-1230, including RSV ([NCT05585632](https://clinicaltrials.gov/ct2/show/NCT05585632)) - Pfizer/BioNTech modRNA ([NCT05596734](https://clinicaltrials.gov/ct2/show/NCT05596734)) - GSK/CureVac mRNA ([NCT06680375](https://clinicaltrials.gov/study/NCT06680375)) - Sanofi RIV3+NVXC19 ([NCT06695130](https://clinicaltrials.gov/study/NCT06695130)) - Sanofi TIV-HD + NVXC19 ([NCT06695117](https://clinicaltrials.gov/study/NCT06695117)) |
| **Milestone 3.2.d**: Determine the utility of further adjusting antigen doses in existing seasonal influenza vaccines for different populations and age-groups.  IN PROGRESS   - [Naleway 2023](https://pubmed.ncbi.nlm.nih.gov/37305842/) compared antibody responses to high-dose egg-based inactivated (HD-IIV3), recombinant (RIV4), and cell culture–based (ccIIV4) vs standard-dose egg-based inactivated (SD-IIV4) influenza vaccine among health care personnel aged 18 to 65 years in 2 influenza seasons. They found that HD-IIV3 did not induce higher antibody responses than SD-IIV4, but, consistent with previous studies, RIV4 was associated with higher post-vaccination antibody titers. - [Pahmeier 2023](https://pubmed.ncbi.nlm.nih.gov/37434014/) used an age-stratified deterministic compartment model to calculate the impact of vaccinating the German population aged 60 years and over for the 2019-2020 season with IIV4-HD compared to standard-dose influenza vaccines (IIV4-SD) with regard to health outcomes and costs. simulate the course of influenza infection for the 2019-2020 season. They found that achieving a higher vaccination coverage with IIV4-SD in persons aged 60 years and older would result in lower costs and fewer influenza infections compared with the scenario with IIV4-HD and actual vaccination rates. |
| **Milestone 3.2.e**: *Conduct clinical trials to assess combinations of licensed seasonal influenza vaccines and vaccine components (e.g., adjuvants) to identify strategies that offer improved effectiveness against influenza.*  IN PROGRESS   - [Hegmann 2025](https://pubmed.ncbi.nlm.nih.gov/40107003/) conducted a phase 1, randomized, double-blind trial among 241 healthy adults 18-45 years of age to evaluate the effects of two newer adjuvants, AF03 (a squalene-based oil-in-water emulsion) or Advax-CpG55.2 (a novel combination adjuvant), used in combination with two different licensed QIVs, Fluzone and Flublok. Results showed that both QIVs were safe and well tolerated when combined with either adjuvant and that there were no significant differences between the immunogenicity elicited by the two adjuvants. |
| *High-Priority Milestone*  **Milestone 3.2.f***:* *Evaluate the immunogenicity and efficacy of alternate routes of vaccine delivery (e.g., intranasal, oral, intradermal needle-free) to enhance mucosal immunity and/or block transmission.*  IN PROGRESS   - [Eiden 2024](https://pubmed.ncbi.nlm.nih.gov/39004096/), [Eiden 2023](https://academic.oup.com/jid/advance-article/doi/10.1093/infdis/jiac433/6807529), [Eiden 2022](https://pubmed.ncbi.nlm.nih.gov/34323977/), and [Eiden 2021](https://pubmed.ncbi.nlm.nih.gov/34960134/) reported on clinical trials evaluating the safety, immunogenicity, and efficacy (via a human challenge study) of FluGen M2SR influenza vaccine administered intranasally. - [Tong 2023](https://journals.aai.org/jimmunol/article-abstract/doi/10.4049/jimmunol.2200956/266461/) compared vaccine-induced humoral immune responses induced by two seasonal influenza vaccines, intramuscular Fluzone IIV and the intranasal LAIV FluMist, to assess whether different vaccine platforms/delivery methods can induce functional humoral immunity in a distinct manner. Results highlighted differences in Ab Fc-effector profiles induced systemically by the two distinct influenza vaccine platforms delivered IM vs IN. - NIH initiated a phase 1 clinical trial [NCT05027932](https://clinicaltrials.gov/ct2/show/NCT05027932) to evaluate beta-propiolactone (BPL)-inactivated quadruple influenza virus cocktail vaccine (BPL-1357) administered intranasally or intramuscularly in two doses 28 days apart. - [Krishnan 2021](https://pubmed.ncbi.nlm.nih.gov/33914729/): US CDC conducted a 2-year RCT of intranasal LAIV versus intramuscular IIV among children aged 2 to 10 years in India, in collaboration with All-India Institute of Medical Sciences (2015-17). - [Liebowitz 2020](https://pubmed.ncbi.nlm.nih.gov/31978354/) evaluated the safety, immunogenicity, and efficacy of Vaxart VXA-A1.1, a human-Ad5-based oral influenza vaccine candidate, in a phase 2 human challenge study; [McIlwain 2021](https://pubmed.ncbi.nlm.nih.gov/34784508/) further analyzed the clinical samples to characterize cellular correlates of protection for VXA-A1.1. - [NCT05397119](https://classic.clinicaltrials.gov/ct2/show/NCT05397119) evaluates the safety and immunogenicity of a novel mucosal recombinant H5 influenza vaccine with a nanoemulsion adjuvant followed by a parenteral boost of licensed inactivated influenza A H5N1 vaccine (phase 1 randomized clinical study).   Several reports describe preclinical evaluations of intranasal, oral, or transdermal routes of delivery of influenza vaccines. Examples include:   - [Nesovic 2023](https://pubs.rsc.org/en/content/articlelanding/2023/BM/D3BM00305A) used a murine model to evaluate the immunogenicity of AuNP-M2e+sCpG, a gold nanoparticle-conjugated M2e vaccine with a soluble CpG adjuvant, administered via microneedle patch. - [Kunzli 2022](https://pubmed.ncbi.nlm.nih.gov/36459542/) found that IM and IN routes of mRNA vaccination in mice influence humoral and cell-mediated immunity, and that IM prime-boosting establishes respiratory tract resident memory T cells (Trm) that can be further enhanced by additional IN immunization. - [Kawai 2021](https://pubmed.ncbi.nlm.nih.gov/34379511/) (see also below under 3.5.d) found that intranasal administration of rNA, but not rHA, conferred cross-protection against antigenically heterologous challenge in mice. - [Stinson 2021](https://pubmed.ncbi.nlm.nih.gov/34391593/) developed a microneedle array patch (MIMIX) designed to release antigen over 1 to 2 weeks, mimicking the time course of an influenza infection, and evaluated it in a preclinical murine model. |
| ***Strategic Goal 3.3:*** *Further assess the role of existing and new adjuvants in creating next-generation seasonal influenza vaccines.* |
| *High-Priority Milestone*  **Milestone 3.3.a***:* *Determine, through clinical studies, if any promising adjuvant candidates under investigation can substantially improve vaccine efficacy in the elderly, and assess their safety profiles.*  IN PROGRESS   - [van der Plas 2024](https://pubmed.ncbi.nlm.nih.gov/38772837/) conducted a first-in-human, randomized, double-blind, controlled, dose-escalation study in the Netherlands, randomizing participants aged 18 to 49 and 65 and older in a 3:1 ratio to receive an intranasal gram-positive enhancer matrix (FluGem), a bacterium-like particle*,* in ascending doses (two-dose regimens) together with a standard TIV or unadjuvanted TIV only; results showed that FluGem was safe and well-tolerated and has potential as a novel mucosal adjuvant in combination with TIV in the elderly. - [Sajkov 2023](https://pubmed.ncbi.nlm.nih.gov/38157402/) evaluated the safety and immunogenicity of trivalent inactivated influenza vaccine (TIV) alone or formulated with Advax delta inulin adjuvant in those of older age (> 60 years) or with chronic disease. Results showed that Advax had a positive impact on anti-influenza IgM responses and on H3N2 and B strain seropositivity as assessed by HAI. - [Shinde 2022](https://pubmed.ncbi.nlm.nih.gov/34563277/) reported that the Novavax Matrix-M-adjuvanted quadrivalent nanoparticle influenza vaccine (qNIV) was well tolerated and produced qualitatively and quantitatively enhanced humoral and cellular immune response in older adults compared with IIV4 (phase 3 randomized clinical trial). - [Crofts 2022](https://pubmed.ncbi.nlm.nih.gov/35455242/) found that R848 increased IgG antibody responses in elderly NHP following responses observed in newborn NHP [preclinical study]. - [Gorse 2022](https://linkinghub.elsevier.com/retrieve/pii/S0264410X22000585) found that MAS-1-adjuvanted IIV(an investigational water-in-oil emulsion-based adjuvant/delivery system comprised of stable nanoglobular aqueous droplets) induced higher HAI antibody responses with prolonged durability including against historical strains, potentially providing greater VE in the elderly throughout an influenza season ([phase 1 study](http://clinicaltrials.gov/show/NCT02500680)). |
| *High-Priority Milestone*  **Milestone 3.3.b***:* *Determine, through clinical studies, if adjuvants substantially improve vaccine efficacy in the very young, and assess their safety profiles.*  IN PROGRESS   - [Barman 2022](https://pubmed.ncbi.nlm.nih.gov/36028220/) found that individually encapsulated and admixed cGAMP-PS and CL075-PS shape the quantity and quality of neonatal immune responses and Th1 polarized neonatal rHA-specific humoral and cell-mediated immune responses [preclinical study in mice]. - [Clemens 2022](http://www.nature.com/articles/s41541-022-00523-8) found that inclusion of TLR7/8 adjuvant R848 in an inactivated IAV vaccine can promote a lasting IgG response to the HA stem. - Seqirus is sponsoring a Phase 2 trial to evaluate responses to vaccination with different MF59 adjuvanted pandemic influenza vaccine formulations of an H5N1 vaccine in pediatric subjects ([NCT04669691](https://clinicaltrials.gov/show/NCT04669691)). |
| ***Strategic Goal 3.4:*** *Determine the role of NA as a vaccine antigen for improving the effectiveness and immunogenicity of seasonal influenza vaccines.* |
| **Milestone 3.4.a***:* *Generate standardized, harmonized, and validated assays for measuring NA content in seasonal influenza vaccines.*  IN PROGRESS   - [Bernard 2022](https://www.frontiersin.org/articles/10.3389/fimmu.2022.909297/full) validated an ELLA-NI SOP for N1 influenza antigen and provided a detailed, harmonized SOP for ELLA-NI. |
| **Milestone 3.4.b**: *Measure and compare the antigenic variation of NA in seasonal influenza vaccines.*  NO PROGRESS |
| **Milestone 3.4.c**: *Investigate options for altering manufacturing processes to retain or add NA to inactivated or recombinant influenza vaccines, and establish methods to confirm, measure, and standardize NA content during development and manufacture.*  NO PROGRESS |
| *High-Priority Milestone*  **Milestone 3.4.d***:* *Determine if the presence of NA improves new or next-generation seasonal influenza vaccines, and, if so, establish the optimal dose of NA that improves immunogenicity and effectiveness and maintains an acceptable safety profile.*  IN PROGRESS     - [Page 2025](https://pubmed.ncbi.nlm.nih.gov/40153499/) developed animal models to investigate mechanisms underlying asymmetric cross-protection between contemporary influenza B viruses lineages (Victoria lineage immunity provides robust cross-protection against the Yamagata lineage, whereas Yamagata immunity offers limited protection against the Victoria lineage). Results indicate that this differential cross-protection is driven by Victoria-elicited NA-specific Abs, which show crosslineage reactivity, unlike those from Yamagata infections. - [Youhanna 2025](https://pubmed.ncbi.nlm.nih.gov/39794704/) collated expert opinions on the importance of NA in influenza vaccines in a two-stage Delphi survey. Results indicated that anti-NA responses induced by infection or vaccination are associated with protective immunity independently of HA and that NA provided additional advantages including improving disease severity metrics. The experts identified several knowledge gaps concerning heterologous cross-reactivity of vaccine-induced anti-NA Abs, correlations between anti-NA titres and reduced transmission or infection risks, and differences in anti-NA responses to seasonal influenza vaccines. - [Cortés 2024](https://www.nature.com/articles/s41541-024-01011-x) demonstrated that recombinant NA (rNA) protein is highly immunogenic in both naïve mice and ferrets, as well as in pre-immune ferrets, irrespective of the level of match with preexisting immunity, and that the addition of rNA components is a viable option to increase the immunogenicity and potential efficacy of already licensed influenza vaccines by means of boosting NA immunity. - [Guzman Ruiz 2024](https://www.frontiersin.org/journals/immunology/articles/10.3389/fimmu.2024.1425842/full) compared the efficacy of NA vaccination in a mouse model administered either as soluble protein or displayed on the surface of VLP vaccine platforms. Results showed that exposure of the N2 on the VLP surface results in strong anti-NA immunity and protection from lethal influenza virus infection. - [Hoy 2024](https://www.medrxiv.org/content/10.1101/2024.06.14.24308936v1) conducted household transmission studies in Managua, Nicaragua, to examine the impact of anti-NA Abs on influenza A/H3N2 susceptibility and infectivity. Results showed that influenza vaccines designed to elicit NA immunity in addition to HA immunity may not only contribute to protection against infection but also reduce infectivity in a household transmission setting. - [Wong 2024](https://pubmed.ncbi.nlm.nih.gov/38250908/) used a new reverse genetics system to generate hybrid LAIV reassortants with HA and NA genes belonging to the opposite IBV lineages, in a new approach to develop IBV vaccines with broad cross-protection. - [Daulagala 2023](https://pubmed.ncbi.nlm.nih.gov/37070986/) characterized the NA antigenicity of seasonal A(H1N1) viruses from 1977 to 1991 to complete the NA antigenic profile of A(H1N1) and A(H1N1)pdm09 viruses circulating in humans. Comparisons of imprinting patterns of anti-HA and anti-NA Ab responses indicated broader cross-reactivity of anti-NA Ab responses than anti-HA Ab responses, supporting the inclusion of NA protein in influenza vaccine preparation to enhance vaccine efficacy.   Several preclinical studies have examined the potential for NA antigens to enhance immunogenicity of different influenza vaccine constructs.   - [Rosu 2022](https://pubmed.ncbi.nlm.nih.gov/35107371/) demonstrated the potential of NA immunity to protect against disease, virus replication in the lower respiratory tract, and virus shedding in the ferret model. - [Strohmeier 2022](https://www.nature.com/articles/s41541-022-00486-w) (NIAID/CIVICs rNA vaccine development) characterized the immunogenicity of CpG 1018-adjuvanted rNA vaccines (N1-MPP, N2-MPP, and B-NA-MPP) in a naïve mouse model. CpG 1018-adjuvanted rNA vaccines induced strong and robust immune response against NA, and provided full protection after high lethal challenges in vivo. - [Gao 2021](https://pubmed.ncbi.nlm.nih.gov/34613807/) found that optimizing the design of rNA (via tetramerization motifs and NA domains included in the rNA construct design) affects the immunogenicity and protective efficacy of the influenza vaccine in mice. - [Kawai 2021](https://pubmed.ncbi.nlm.nih.gov/34379511/) found that NA antigen in an intranasal vaccine confers broad cross-protection in the upper respiratory tract by inducing NA-specific IgA that recognizes a wide range of epitopes in mice. |

**TOPIC 4: VACCINOLOGY FOR BROADLY PROTECTIVE OR UNIVERSAL INFLUENZA VACCINES**

| ***Strategic Goal 4.1:*** Identify the most promising broadly protective or universal influenza vaccine candidates that elicit durable protection against influenza viruses in preclinical studies, with a focus on targeting conserved regions of the virus. |
| --- |
| **Milestone 4.1.a***:* *Develop a set of preferred product characteristics (PPCs) for broadly protective and universal influenza vaccines, in collaboration with the WHO’s efforts to revise its 2017 guidance on PPCs for next-generation influenza vaccines.*  IN PROGRESS     - [Bresee 2023](https://pubmed.ncbi.nlm.nih.gov/37872012/): WHO is updating the [2017 PPCs](http://apps.who.int/iris/bitstream/10665/258767/1/9789241512466-eng.pdf) for Next-Generation Influenza Vaccines to guide R&D efforts towards improved influenza vaccines suitable for use in low-resource settings (publication expected in 2025). |
| **Milestone 4.1.b***:* *Develop a summary analysis of influenza vaccine approaches for broadly protective or universal influenza vaccines and create a mechanism to update this summary at least annually.*  ACCOMPLISHED   - CIDRAP developed and maintains the [Universal Influenza Vaccine Technology Landscape](https://ivr.cidrap.umn.edu/universal-influenza-vaccine-technology-landscape), a database of potentially universal, broadly protective, and next-generation influenza vaccines, as defined in the IVR. - [Mosmann 2024](https://pubmed.ncbi.nlm.nih.gov/38698082/) summarized T-cell based influenza vaccines in development. - [Taaffe 2024](https://pubmed.ncbi.nlm.nih.gov/39369576/) analyzed universal, broadly protective, and next-generation influenza vaccines in clinical development, as a component of the WHO FVIVA process. |
| *High-Priority Milestone*  **Milestone 4.1.c:** *Continue research on new antigen design to enable the development of broadly protective or universal influenza vaccines.*  IN PROGRESS     - NIAID is evaluating a computationally designed mosaic nanoparticle composed of full-length HA trimers from 6 different influenza A and B strains in phase 1 trials [NCT06863142](https://clinicaltrials.gov/study/NCT06863142) and [NCT05968989](https://clinicaltrials.gov/study/NCT05968989). - [Houser 2022](https://www.nature.com/articles/s41591-021-01660-8) conducted a phase 1 trial ([NCT03186781](https://clinicaltrials.gov/ct2/show/NCT03186781)) to evaluate a novel H2HA-ferritin nanoparticle vaccine candidate, composed of 8 antigenically intact influenza H2 HA trimers arrayed on the nanoparticle surface. - NIAID developed and evaluated two stabilized (headless) HA stem-only nanoparticle vaccine candidates ([Andrews 2023](https://www.science.org/doi/10.1126/scitranslmed.ade4976), [Widge 2023](https://www.science.org/doi/10.1126/scitranslmed.ade4790)). - Osivax (France) is developing the OVX836 nanoparticle based on the nucleoprotein (NP) antigen from influenza A and B strains ([Jacobs 2024](https://www.mdpi.com/2076-393X/12/12/1391)). - Recombinant HA antigens using computationally optimized broadly reactive antigens (COBRA) are under development with various vaccine platform technologies, including whole inactivated virus or split inactivated virus formulations ([Shi 2025](https://pubmed.ncbi.nlm.nih.gov/39982912/)), mRNA-lipid nanoparticles ([Allen 2024](https://pmc.ncbi.nlm.nih.gov/articles/PMC10963417/); [Hendy 2024](https://www.sciencedirect.com/science/article/abs/pii/S016836592400350X)), recombinant NA ([Uno 2024](https://journals.asm.org/doi/10.1128/jvi.00354-24)), recombinant HA ([Allen 2024](https://www.mdpi.com/2076-393X/12/12/1364)), virus-like particles ([Zhang 2024](https://doi.org/10.1080/21645515.2024.2403175)), and recombinant adenovirus-associated virus vectors ([Wiggins 2024](https://pubmed.ncbi.nlm.nih.gov/39078191/)). - [Arevalo 2022](https://pubmed.ncbi.nlm.nih.gov/36423275/) developed a multivalent mRNA-lipid nanoparticle vaccine candidate encoding HA antigens from all 20 known influenza A and B virus subtypes. - [Oftung 2022](https://www.mdpi.com/2076-393X/10/9/1528) incorporated conserved regions of the M1, M2, NP-A, and NP-B influenza virus proteins in a peptide-based influenza vaccine candidate. |
| *High-Priority Milestone*  **Milestone 4.1.d***:* *Convene a workshop to review the scientific challenges and recent advancements in development of novel vaccine platforms to determine how best to guide R&D for broadly protective influenza vaccines for regulatory approval, global scale-up, and rapid deployment in response to pandemic influenza viruses.*  IN PROGRESS   - Although a workshop has not been convened, interest in applying novel platforms to seasonal influenza vaccines is evident; 10 mRNA-based next-generation influenza vaccine candidates are now in clinical development (see [Landscape](https://ivr.cidrap.umn.edu/universal-influenza-vaccine-technology-landscape)). - [Kok 2024](https://pubmed.ncbi.nlm.nih.gov/39553979/) developed a high-resolution antigenic map of the global antigenic evolution of avian influenza A(H5) HA to engineer 30 vaccine HA antigens designed to elicit Ab responses that broadly cover the A(H5) antigenic space. |
| *High-Priority Milestone*  **Milestone 4.1.e***:* *Define selection criteria (e.g., including immunogenicity data from pre-immune animal models and considerations for global manufacturing capacity) for advancing preclinical influenza vaccine candidates into clinical evaluation.*  IN PROGRESS   - [Särnefält 2024](https://pubmed.ncbi.nlm.nih.gov/39054065/): CEPI developed a CMC (Chemistry, Manufacturing, and Controls) framework to expedite the progression of vaccine candidates from research to deployment, reduce delays, mitigate risks, and optimize the overall development process. - Multiple public and private sector sponsors have selected a wide range of candidates for clinical trials based on positive results in animal models (see [Landscape](https://ivr.cidrap.umn.edu/universal-influenza-vaccine-technology-landscape#database) for a full list of trials and publications). |
| ***Strategic Goal 4.2:*** Evaluate the most promising broadly protective or universal influenza vaccine candidates, using at least several different platforms, in clinical trials. |
| **Milestone 4.2.a:** *Develop use cases for broadly protective vaccines, including, for example, pan-H5 vaccines for pandemic response, to define how, where, and under what circumstances such vaccines would be used.*  IN PROGRESS     - WHO’s FVIVA project, aimed at identifying critical success factors that would enable the development, approval, and introduction of next-generation influenza vaccines, includes the development of use cases (see also Milestone 6.1.a). |
| *High-Priority Milestone*  **Milestone 4.2.b:** *Define and prioritize clinical endpoints for evaluating the efficacy of broadly protective influenza vaccines (e.g., prevention of laboratory-confirmed influenza, severe complications of influenza, hospitalization, death) to compare outcomes across studies.*  NO PROGRESS |
| *High-Priority Milestone*  **Milestone 4.2.c***:* *Identify an initial set of vaccine candidates that demonstrate broad-based immunity—humoral, cell-mediated, or both—in preclinical research and assess them for safety and immunogenicity in phase 1 clinical trials in healthy adults.*  ACCOMPLISHED   - Potentially universal, broadly protective, or next-generation influenza vaccine candidates in phase 1 trials include (see [Landscape](https://ivr.cidrap.umn.edu/universal-influenza-vaccine-technology-landscape) for links to clinical trials and publications), - A large and diverse group of candidates are in active preclinical development (see [Landscape](https://ivr.cidrap.umn.edu/universal-influenza-vaccine-technology-landscape)). |
| *High-Priority Milestone*  **Milestone 4.2.d***:* *Determine biomarkers correlated with immune protection for different vaccine platforms.*  IN PROGRESS   - [ISIRV](https://www.isirv.org/site/) conference, *Correlates of protection for next generation influenza vaccines: lessons learned from the COVID pandemic*, held Mar 1-3, 2023, focused on immunologic assays and correlates of protection for evaluating next-generation influenza vaccines. The next ISIRV Correlates of Protection conference will be held in Vienna in October 2025. - [Wellcome Trust 2023](https://wellcome.org/reports/towards-reformed-research-and-development-ecosystem-infectious-disease), *Toward a reformed research and development ecosystem for infectious disease,* highlights the need for greater support for regulatory science tools, standards and approaches, such as identifying correlates of protection, to support safety and efficacy determination in the development of novel vaccines. - US CDC is developing serological assays to measure correlates of protection for the next‐generation influenza vaccines. |
| *High-Priority Milestone*  **Milestone 4.2.e***:* *Continue to analyze outcomes from broadly protective vaccine candidates in phase 1 trials and advance promising candidates into phase 2 and phase 3 clinical trials, including in high-risk populations.*  PARTIALLY ACCOMPLISHED   - [WHO SAGE 2022](https://www.who.int/publications/i/item/who-wer9719) defined “high-risk” groups for developing severe disease resulting in hospitalization or death (such as older adults, pregnant women and women up to 2 weeks postpartum, children, people with a BMI or 40 or higher, and people with comorbidities) and those at increased risk of exposure to or transmission of influenza virus (such as health workers). - Several potentially universal, broadly protective, or next-generation influenza vaccine candidates are in phase 2 trials (see [Landscape](https://ivr.cidrap.umn.edu/universal-influenza-vaccine-technology-landscape) for links to clinical trials and publications). |
| *High-Priority Milestone*  **Milestone 4.2.f***:* *Identify the most promising vaccine candidates from phase 2 trials and facilitate good manufacturing practice (GMP) and evaluation in phase 3 trials, such as through sponsorship in public-private partnerships.*  IN PROGRESS   - No potentially universal or broadly protective influenza vaccines have reached phase 3 trials. However, several next-generation, improved seasonal vaccine candidates are currently in phase 3 trials (see [Landscape](https://ivr.cidrap.umn.edu/universal-influenza-vaccine-technology-landscape) for links to clinical trials and publications). |

**TOPIC 5: ANIMAL MODELS AND THE CONTROLLED HUMAN INFLUENZA VIRUS INFECTION MODEL (CHIVIM)**

| ***Strategic Goal 5.1:*** Optimize animal models for influenza vaccine research. |
| --- |
| **Milestone 5.1.a***:* *Sustain mechanisms for broad sharing of animal model resources (e.g., standardized and validated viruses, protocols, reagents, and tissue samples) that can be used among a wide range of investigators as standards for influenza vaccine research, which is particularly important given constraints on the availability of experimental animals.*  PARTIALLY ACCOMPLISHED   - NIAID created a centralized repository of research resources, [Flu Hub](https://www.fluhub.org/), to advance influenza research supporting the NIAID Universal Influenza Vaccine Strategic Plan ([Erbelding 2018](https://academic.oup.com/jid/article/218/3/347/4904047)). Flu Hub provides information on available research facilities, preclinical services, clinical resources, reagents, datasets, and bioinformatics tools. |
| *High-Priority Milestone*  **Milestone 5.1.b***:* *Sustain funding for validated reagents, updated viral stocks, and harmonized assays, which are needed to evaluate innate and adaptive immune responses in ferrets and other animals such as hamsters and to facilitate comparison of studies across laboratories.*  PARTIALLY ACCOMPLISHED   - NIAID CEIRR provides access to the scientific community to over 1,000 free and unique [immunological reagents for the ferret model](https://www.ceirr-network.org/resources/ferret-reagents) produced by CEIRR investigators. |
| **Milestone 5.1.c***:* *Harmonize best practices for conducting influenza virus transmission studies in animals such as hamsters and guinea pigs, as was done in ferrets, to include naive and infected or vaccinated animals.*  IN PROGRESS   - [Belser 2022](https://pubmed.ncbi.nlm.nih.gov/35862762/) conducted a cross-laboratory standardization exercise for influenza risk assessment studies in ferrets to improve the interpretation of transmission data generated during risk assessment activities. - CEPI established a [preclinical models network](https://cepi.net/research_dev/vaccine-science/) for testing vaccines against priority pathogens, which could serve as a guide in influenza vaccine development, which may support the development of models other than ferrets and the resolution of regulatory issues involving preclinical data requirements. |
| **Milestone 5.1.d***:* *Continue development of pre-exposure animal models to address the fact that humans generally have pre-existing immunity to influenza.*  IN PROGRESS   - NIAID conducted a workshop in 2024 on animal models for SARS-CoV-2 and influenza, which included evaluation of preexisting immunity to influenza and a report is in progress. |
| *High-Priority Milestone*  **Milestone 5.1.e**: *Develop comparative vaccine studies using the high-risk ferret model to inform predictions of vaccine responses in high-risk human populations.*  IN PROGRESS   - NIAID established research projects [([PAR-19-247](https://grants.nih.gov/grants/guide/pa-files/par-19-247.html) (R21) and [PAR-19-248](https://grants.nih.gov/grants/guide/pa-files/PAR-19-248.html) (R01)] to improve the predictive value of animal models, including ferret models, for evaluating novel universal influenza vaccines. - [Knoll 2024](https://pubmed.ncbi.nlm.nih.gov/38785423/) used a diet-induced obesity ferret model and the A/Hong Kong/1073/1999 (H9N2) strain to evaluate the impact of host obesity on viral genetic variation and adaptation. Results suggested that host obesity may result in a unique selective environment affecting intrahost IAV evolution. - [Meliopoulos 2023](https://pubmed.ncbi.nlm.nih.gov/37808835/) use a novel diet-induced obese ferret model to demonstrate that obesity resulted in significant changes to the lung microenvironment leading to increased clinical disease and viral spread to the lower respiratory tract. - [Thakur 2023](https://pubmed.ncbi.nlm.nih.gov/37739789/) summarizes copathogenesis studies using ferrets as a model system for investigating influenza virus-bacteria interactions. - [Sutton 2022](https://pubmed.ncbi.nlm.nih.gov/36300929/) evaluated sequential rounds of airborne transmission as an approach to enhance the predictive accuracy of the ferret model. |
| *High-Priority Milestone*  **Milestone 5.1.f***:* *Review a comprehensive analysis of the predictive value of different animal models, including natural hosts such as pigs and horses, for influenza vaccine studies (for both seasonal and broadly protective vaccines).*  IN PROGRESS   - [Kanekiyo 2023](https://pubmed.ncbi.nlm.nih.gov/37766976/) refined the lethal H5N1 infection macaque model to assess the relationship between inhaled dose and disease course, and to evaluate whether vaccination against seasonal influenza using an adjuvanted QIV could offer protection from mortality and/or morbidity caused by the HPAI H5N1 infection in macaques. - [Yuan 2023](https://pubmed.ncbi.nlm.nih.gov/36992230/) developed swine model system (pregnant sow-neonate model) to evaluate the impact of influenza vaccination on maternal immunity and fetal/neonatal development. - [Dibben 2021](https://pubmed.ncbi.nlm.nih.gov/33712628/) defined a low, ferret-appropriate vaccine dose for LAIV, suggesting a key role for the ferret model in dose optimization and LAIV strain selection. |
| *High-Priority Milestone*  **Milestone 5.1.g**: *Develop and validate animal models for evaluating immune responses—including durability and mucosal immunity—to broadly protective influenza vaccines.*  IN PROGRESS   - [Wei 2025](https://pubmed.ncbi.nlm.nih.gov/40266241/) developed the HLA-A2/DR1 and HLA-A11/DR1 transgenic mouse models for direct screening and validation of influenza virus T-cell epitopes for use in designing T cell vaccines against influenza viruses. - [Sanders 2024](https://pubmed.ncbi.nlm.nih.gov/38286428/) used three different “dirty” mouse models with different additional microbial experience, compared with commonly used specific pathogen-free mice, to determine how previous microbial exposure affects subsequent immune responses to influenza vaccination and to determine which models best recapitulate human immune responses to vaccination. Results showed differing responses among the mouse models to prior exposure environments and to the magnitude of immune responses to vaccination, suggesting that different model systems may be needed to recapitulate the range of human immune responses to vaccination. - [Schewe 2024](https://pubmed.ncbi.nlm.nih.gov/39591178/) demonstrated the ability of a ferret efficacy model, optimized for a low vaccine dose and clinically relevant endpoints, to reproduce clinical immune responses to H1N1 LAIV. - NIAID-supported research is in progress on the predictive value of animal models (mouse, ferret, pig, and hamster) in recapitulating human immunity to influenza infection and vaccination (e.g., [PAR-19-247](https://grants.nih.gov/grants/guide/pa-files/par-19-247.html) and [PAR-19-248](https://grants.nih.gov/grants/guide/pa-files/par-19-248.html) awards). - [Vazquez-Pagan 2023](https://pubmed.ncbi.nlm.nih.gov/37961247/) established a novel ultrasound-based mouse pregnancy model and used it to evaluate the efficacy and immunogenicity of distinct influenza vaccine platforms administered during pregnancy and how these may influence the response to influenza infection in the offspring. - [Fiege 2021](https://pubmed.ncbi.nlm.nih.gov/34731647/) found that laboratory mice exposed to pathogens from pet-store mice exhibit impaired humoral immunity to influenza vaccination and display gene expression signatures that more authentically reflect human vaccine responses. |
| **Milestone 5.1.h**: *Characterize the major histocompatibility complex (MHC) epitopes in the ferret model to better allow assessment of T cell–based vaccines.*  IN PROGRESS   - [Walsh 2023](https://papers.ssrn.com/sol3/papers.cfm?abstract_id=4557205) developed new B- and T-cell receptor assays, (e.g., single-cell based Ig TCR assays), to reflect ferret immune repertoire diversity. |
| ***Strategic Goal 5.2:*** Address steps needed to further develop and refine the CHIVIM. |
| *High-Priority Milestone*  **Milestone 5.2.a***:* *Generate guidance, including ethical and safety considerations, for using the CHIVIM.*  ACCOMPLISHED   - [Lane 2024](https://pubmed.ncbi.nlm.nih.gov/39440405/) summarized the presentations, discussions, key takeaways, and future directions for innovations in CHIVIMs from a workshop (*CHIVIM studies: current status and future directions for innovation*, Nov. 13-14, 2023) convened by NIAID on use cases and regulatory and ethical considerations for CHIVIM studies; participants included industry, academic, and government researchers. This meeting was a follow-up to the 2018 meeting in London on the influenza human challenge model for universal influenza vaccine development ([Innis 2019](https://pubmed.ncbi.nlm.nih.gov/31362819/)). - [UK Pandemic Ethics Accelerator 2023](https://ukpandemicethics.org/wp-content/uploads/2023/03/The-ethics-of-controlled-human-infection-model-studies-for-mitigating-pandemic-risks-Report.pdf) provides guidance on when it may be appropriate to conduct controlled human infection model studies and on the requirements for their ethical conduct. - [Wellcome Trust 2023](https://wellcome.figshare.com/articles/online_resource/Use_of_Human_Infection_Studies_in_Vaccine_Development/21997619/1) summarizes outcomes of an international multi-stakeholder meeting on how human infection study data can inform regulatory approval of new vaccines. - [Abo 2023](https://pubmed.ncbi.nlm.nih.gov/37573871/) reviewed vaccine development following trials in human challenge models, highlighting the potential for human challenge trials to accelerate development of vaccines against priority pathogens and in pandemic and non-pandemic settings. - [WHO 2022](https://www.who.int/publications/i/item/9789240037816) provides global guidance on the ethical conduct of controlled human infection studies, which includes language on influenza. - [Williams 2022](https://pubmed.ncbi.nlm.nih.gov/35210119/) reviews the early efforts of international and national institutions to define the ethical standards required for COVID-19 human challenge studies and create the frameworks to ensure rigorous and timely review of these studies. - [Dayananda 2022](https://pubmed.ncbi.nlm.nih.gov/35704096/) reviews the use of human challenge models in RSV vaccine R&D, including ethical and logistical considerations, potential benefits, their role in streamlining and accelerating novel vaccines, and potential extension to include relevant at-risk populations. - [HIC-Vac](https://www.hic-vac.org/) (UK) provides information and expertise regarding the use of human infection challenge studies for vaccine R&D particularly in the UK and LMICs. |
| *High-Priority Milestone*  **Milestone 5.2.b***:* *Ensure the broad availability to investigators of a biorepository of diverse, well-characterized challenge viruses, cell lines, and human samples for manufacture and CHIVIM studies.*  IN PROGRESS   - NIAID is in the process of making challenge strains available for H3N2 and H1N1. - NIAID released the [Notice to “Inform the Extramural Influenza Research Community of the Availability of Materials for Controlled Human Influenza Virus Infection Model (CHIVIM) Clinical Studies and Trials](https://grants.nih.gov/grants/guide/notice-files/NOT-AI-23-067.html) (Sep 15, 2023). |
| **Milestone 5.2.c**: *Share data, information, and protocols across multiple centers conducting CHIVIM studies to maximize benefit.*  IN PROGRESS   - [Ortiz 2023](https://pubmed.ncbi.nlm.nih.gov/36702771/) conducted a multicenter CHIVIM study ([NCT04044352](http://clinicaltrials.gov/show/NCT04044352)) to assess clinical response, immunological response, and safety of intranasal administration of (H1N1)pdm09 virus in healthy adults; the study was reported to have advanced NIAID goals to expand capacity for conducting CHIVIM studies by collecting substantial data and specimens from the 4 centers for future immunologic analyses alternative clinical outcome definitions. |

**TOPIC 6: POLICY, FINANCING, AND REGULATION**

| ***Strategic Goal 6.1:*** Catalyze broad support and sustained funding for developing improved seasonal influenza vaccines and broadly protective or universal influenza vaccines. |
| --- |
| *High-Priority Milestone*  **Milestone 6.1.a***:* *Develop and disseminate a full value of vaccine assessment (FVVA; also referred to as the full value of influenza vaccine assessment [FVIVA]) for improved seasonal and broadly protective, universal influenza vaccines that addresses different vaccine use cases and includes an assessment for low- and middle-income countries (LMICs).*  IN PROGRESS   - [White 2024](https://pubmed.ncbi.nlm.nih.gov/38675817/) summarized WHO’s FVVA framework for documenting evidence for the value of vaccines, including the broader socioeconomic consequences of vaccines, and prioritizing vaccine candidates for investment and deployment in LMICs. - [Bresee 2023](https://pubmed.ncbi.nlm.nih.gov/37872012/): WHO is developing the FVIVA, which is expected to be completed by late 2024 and includes three workstreams: (1) vaccine products and supply; (2) market potential for next-generation influenza vaccines; and (3) expected health and economic impact of global distribution and uptake of next-generation seasonal influenza vaccines. - [Hutubessy 2023](https://pubmed.ncbi.nlm.nih.gov/37400797/) published a conceptual framework for the FVVA, designed to: (1) guide the assessment and communication regarding new vaccines, (2) facilitate alignment across key stakeholders, and (3) enhance decision-making on investment in vaccine development, policymaking, and deployment particularly for vaccines intended for use in LMICs. |
| *High-Priority Milestone*  **Milestone 6.1.b***:* *Develop targeted and creative communications and advocacy strategies and necessary communication tools that build on the FVIVA and provide information on economic costs, the risk of future influenza pandemics, and the need for investment in influenza vaccine R&D.*  IN PROGRESS   - The Sabin Vaccine Institute’s [Influenzer Initiative Resource Library](https://www.playbook.com/s/influenzerinitiative/Bu7VeV2p4h4AtAM8sSkv4Jv8/SQQ3YoWLucjpCCLH6sz5SSZD) is an asset library comprising 43 video and audio elements, now hosted on the CIDRAP IVR website, and providing communications and education on collaborative vaccine distribution systems, investment in sustainable influenza vaccine development and its connection to health and economic security, the potential of mRNA vaccine technology, and the role of influenza vaccine R&D in pandemic prevention. - WHO’s FVIVA will include communication tools and advocacy strategies. - [Fisman 2024](https://pubmed.ncbi.nlm.nih.gov/38835218/) summarized published cost-effectiveness analyses of cell-based influenza vaccines in children and adults less than 65 years of age, critically assessed the assumptions and approaches used in these analyses, and considered the role of cell-based influenza vaccines for children and adults. - [Goodfellow 2024](https://www.medrxiv.org/content/10.1101/2024.09.19.24313950v1) estimated the cost-effectiveness of different vaccination scenarios for universal, broadly protective, next-generation, and current seasonal influenza vaccines and used modelling to project the health and economic impact of the vaccines in 186 countries and territories. Results showed that improved influenza vaccines have the potential to substantially reduce influenza burden and be cost-effective in lower income countries. - [Langer 2024](https://pubmed.ncbi.nlm.nih.gov/38261171/) conducted a systematic literature review on the humanistic burden of influenza in adults 65 and older, considering the impact of influenza on a patient’s health-related quality of life, daily activities, and caregiver health and/or quality of life, and economic findings, including direct and indirect costs associated with influenza. - [Procter 2024](https://pubmed.ncbi.nlm.nih.gov/39557448/) used a combined epidemiologic and economic model to estimate the health impact and cost-effectiveness of next-generation influenza vaccines (NGIVs) in Thailand. Results showed that adoption of NGIVs could substantially reduce the burden of influenza in Thailand and are likely to be cost-effective, but that there could also be large up-front costs. - [Krauland 2023](https://pubmed.ncbi.nlm.nih.gov/36536801/) modeled the impact of more effective influenza vaccines on the burden of seasonal influenza and found that highly effective vaccines could dramatically reduce influenza burden. - [Langer 2023](https://pubmed.ncbi.nlm.nih.gov/36790682/) characterized the global clinical burden of seasonal influenza among adults 65 years of age and older and concluded that influenza exerts a considerable burden on older adults and healthcare systems, with high incidence of hospitalization and mortality. - [Maleki 2023](https://pubmed.ncbi.nlm.nih.gov/37470942/) characterized the global burden of influenza in the 18 to 64 years population, based on a systematic review of influenza-associated clinical and economic outcomes in several global regions. They concluded that high levels of hospitalization and outpatient visits demonstrated a clinical influenza-associated burden on patients and healthcare systems, which is exacerbated by comorbidities. - [Waterlow 2023](https://pubmed.ncbi.nlm.nih.gov/37633749/) used mathematical modelling to assess the potential health, epidemiologic, and economic impact of next-generation influenza vaccines in England and Wales, providing evidence for the FVVA and an investment case for their development and rollout. - [Waterlow 2023](https://pubmed.ncbi.nlm.nih.gov/36949456/) evaluated the cost-effectiveness of next-generation influenza vaccines in children under 5 years of age in a low-income country with year-round influenza seasonality (Kenya). |
| **Milestone 6.1.c**: *Create, implement, and maintain a mechanism to track influenza vaccine R&D funding trends to better assess where funding is being allocated and identify gaps in funding for priority research.*  ACCOMPLISHED   - CIDRAP at the University of Minnesota, with funding from the Wellcome Trust, developed and maintains the [IVR Funding Tracker](https://ivr.cidrap.umn.edu/ivr-funding-tracker-dashboard), a database of global funding aligned with priorities identified in the [roadmap](https://ivr.cidrap.umn.edu/roadmap). |
| **Milestone 6.1.d**: *Determine the feasibility of establishing a new public-private partnership, with robust funding, to identify and implement creative approaches to de-risk and accelerate development of universal, broadly protective, and next-generation influenza vaccines for pandemic preparedness and seasonal influenza.*  NO PROGRESS |
| ***Strategic Goal 6.2:*** Promote innovation for developing improved seasonal influenza vaccines and broadly protective or universal influenza vaccines. |
| *High-Priority Milestone*  **Milestone 6.2.a***:* *Distill lessons learned for influenza vaccines from experience with COVID-19 vaccine R&D, including clinical research and study designs, manufacturing, distribution, advocacy, financing, and global collaboration.*  ACCOMPLISHED   - [Krammer 2023](https://pubmed.ncbi.nlm.nih.gov/37436465/) reviewed COVID-19 vaccine development during the pandemic and highlighted scientific, technological, and policy issues that need to be addressed for pandemic preparedness, including the need to provide sufficient R&D funding and to counteract vaccine hesitancy, misinformation, and anti-vax conspiracy movements. - [Palache 2023](https://pubmed.ncbi.nlm.nih.gov/37598027/) (IFPMA) summarized lessons learned for vaccine manufacturing during a pandemic (e.g., regarding pathogen surveillance and data sharing, equitable distribution, and pharmaceutical partnerships to accelerate R&D and manufacturing). - [Arinaminpathy 2022](https://pubmed.ncbi.nlm.nih.gov/35482858/) summarized lessons learned for influenza vaccine R&D from the COVID-19 pandemic in the topic areas of epidemiological implications, economic implications, global production capacity, and roles for donors and policy-makers. - [Ballou 2022](https://nam.edu/the-influenza-imperative-an-urgent-need-to-leverage-lessons-from-covid-19-to-prepare-for-a-global-response-to-seasonal-and-pandemic-influenza/), [NASEM 2022](https://nap.nationalacademies.org/catalog/26352/international-workshop-on-covid-19-lessons-to-inform-pandemic-influenza-response): the [National Academy of Medicine](https://pubmed.ncbi.nlm.nih.gov/35171549/) (collaborator: US CDC/[PIVI](https://pivipartners.org/about-pivi/)) convened a workshop focused on lessons learned from COVID-19 to inform and advance pandemic and seasonal influenza vaccine preparedness efforts and subsequent response. - [Johnson 2022](https://pubmed.ncbi.nlm.nih.gov/36302122/) (BARDA) summarized lessons learned from the development of COVID-19 vaccines, including gaps and challenges that must be addressed to prepare for future outbreaks caused by pandemic viruses. |
| **Milestone 6.2.b**: *Maintain engagement with industry partners to regularly examine market challenges and evaluate potential solutions, including market incentives to help de-risk vaccine R&D and develop a market share for producing improved influenza vaccines.*  IN PROGRESS   - [Taaffe 2025](https://pubmed.ncbi.nlm.nih.gov/39970592/) provided an updated assessment of the global production capacity for seasonal and pandemic influenza vaccines, based on WHO’s 2023 manufacturer survey. The results suggest that seasonal influenza vaccine production capacity has remained relatively stable since 2019 at 1.53 billion doses and pandemic vaccine capacity at 4.13 and 8.26 billion doses for moderate and best case scenarios, respectively. Production is concentrated in high and upper-middle income countries, indicating a global imbalance that could result in unequal access to vaccines in LMICs during a pandemic. - [WHO 2024](https://cdn.who.int/media/docs/default-source/immunization/mi4a/who_mi4a_global_market_study_seasonal_influenza_vaccine.pdf?sfvrsn=9f24acbf_3&download=true) published a report, *Global Market Study: Seasonal Influenza Vaccine*, examining global trends and drivers of seasonal influenza vaccine supply and demand; key findings include: (1) demand for seasonal influenza vaccines is stable and highly concentrated in high- and upper-middle income countries, which together consume over 95% of seasonal influenza vaccines; (2) only 27 (34%) LMICs reported having a policy for seasonal influenza vaccination; (3) the seasonal influenza vaccine supplier base is large, but more than 85% of annual volumes are supplied by seven producers; (4) demand for seasonal influenza vaccines is forecasted to increase by ~10% in the next 10 years and there is significant potential to increase seasonal influenza vaccine use, particularly in LMICs; and (5) in the short term, limited access risks exist due to the regulatory and implementation complexities necessitated by WHO’s recommended removal of the B/Yamagata strain from QIVs. |
| **Milestone 6.2.c**: *Similar to the “evidence to recommendation” framework created by the WHO Strategic Advisory Group of Experts on Immunizations Working Group on COVID-19 vaccines, create a framework that identifies the critical evidence needed to inform (1) the design of clinical trials for universal or broadly protective influenza vaccines and (2) policy recommendations for the use of universal or broadly protective influenza vaccines once they are approved.*  IN PROGRESS   - [Yang 2024](https://pubmed.ncbi.nlm.nih.gov/38944025/) used modeling to evaluate vaccine formulation and strategies, based on host immunological history and the co-existence of groups 1 and 2 HA strains, and developed a vaccine population-level target product profile (PTPP) for influenza vaccines showing that a future vaccine providing sufficiently broad and long-lived cross-group protection at a sufficiently high vaccination rate could prevent pandemic emergence and lower the pandemic burden. - WHO Strategic Advisory Group of Experts (SAGE) on Immunization published a 2022 [position paper on seasonal influenza vaccines](https://www.who.int/publications/i/item/who-wer9719), including [“evidence to recommendation” tables](https://cdn.who.int/media/docs/default-source/immunization/position_paper_documents/influenza/influenza-sage-annexes-04-05-22.pdf?sfvrsn=566a6082_1). |
| **Milestone 6.2.d**: *Develop an influenza prevention and control roadmap to advance the WHO Global Influenza Strategy that addresses health equity issues, encompasses the interests of LMICs—including the need for improved seasonal influenza vaccines—and is aimed at supporting country transitions from annual vaccination programs to the use of more durable, broadly protective or universal vaccines.*  IN PROGRESS   - The Partnership for International Vaccine Initiatives (PIVI) at the Task Force for Global Health is developing the [Roadmap for Influenza Prevention and Control](https://pivipartners.org/roadmap-for-influenza-prevention-and-control-request-for-expressions-of-interest-to-participate-in-the-technical-expert-group-teg/) to support implementation of the WHO Global Influenza Strategy, 2019-2030. The roadmap is expected to be completed by the end of 2025. - [Chadwick 2022](https://pubmed.ncbi.nlm.nih.gov/35810059/) summarized WHO efforts to assess outcomes and challenges with the Technology Transfer Initiative, a program that facilitates vaccine production capacity-building in LMICs, including production of next-generation influenza vaccines. - [Scarnà 2023](https://pubmed.ncbi.nlm.nih.gov/36649573/) identified potential benefits of vaccine microarray patches in LMICs for epidemic or pandemic response. |
| ***Strategic Goal 6.3:*** Promote information sharing aimed at moving influenza vaccine development forward. |
| **Milestone 6.3.a***:* *Create and maintain a comprehensive landscape of universal influenza vaccine technologies in preclinical and clinical development and develop a mechanism to update and analyze the landscape, including identifying key factors underlying successful R&D efforts as well as persistent challenges and obstacles.*  ACCOMPLISHED   - CIDRAP at the University of Minnesota, with funding from the [Global Funders Consortium for Universal Influenza Vaccine Development](https://unifluvac.org/), developed and maintains the [Universal Influenza Vaccine Technology Landscape](https://ivr.cidrap.umn.edu/universal-influenza-vaccine-technology-landscape), which is updated regularly. - [Taaffe 2024](https://pubmed.ncbi.nlm.nih.gov/39369576/) reviewed the next-generation influenza vaccine pipeline, focusing on products in clinical development, and compared their characteristics to currently approved seasonal influenza vaccines. |
| *High-Priority Milestone*  **Milestone 6.3.b***:* *Assess and document the ongoing impact of the Nagoya Protocol and possibly related national Access and Benefit Sharing (ABS) legislation, on sharing of influenza isolates and gene sequences in relation to influenza vaccine R&D; share findings with key policymakers (e.g., ministries of health/agriculture and global trade); and determine strategies to address potential unintended consequences.*  IN PROGRESS   - US CDC has supported this work through WHO working groups and input to the WHO World Health Assembly. - WHO has published several reports on the public health implications of the Nagoya protocol:   - Implementation of Decision WHA72(12), which included requests related to influenza virus sharing:   - [Report on influenza virus sharing](https://cdn.who.int/media/docs/default-source/pip-framework/governance/wha72-12-op1a-report-edited_en1a2d0386-152a-4bbd-b801-9ec5d71e8930.pdf?sfvrsn=80f75c02_16)   - [Summary report on national legislation and regulatory measures related to influenza](https://cdn.who.int/media/docs/default-source/pip-framework/governance/wha72-12-op1b-report-edited-en.pdf?sfvrsn=c4527c37_36)   - [Public health implications of the Nagoya Protocol](https://apps.who.int/gb/ebwha/pdf_files/EB148/B148_21-en.pdf) - [IFPMA](https://www.ifpma.org/publications/applying-lessons-learned-from-covid-19-to-create-a-healthier-safer-more-equitable-world/) noted agreement “across UN agencies and member states, along with science, public health, and legal experts, civil society, foundations, and industry… that access to pathogen samples and their genetic information is fundamental to improved preparedness and response.” One of the “approaches under consideration to improve pandemic preparedness and response related to pathogen sharing” includes “changes to the Convention on Biological Diversity’s Nagoya Protocol expressly to exclude outbreak pathogens…”. |
| **Milestone 6.3.c***:* *Implement a plan that improves existing data management and sharing among influenza R&D researchers that includes developing an approach for reusing influenza vaccine study data (e.g., secondary mining of datasets).*  ACCOMPLISHED  The NIAID [ImmPort](https://immport.niaid.nih.gov/home) is an open-access platform for research data sharing from basic research and clinical trials, designed to allow users to analyze data (e.g., from NIAID CIVICs and CEIRR studies), visualize results, and integrate data from multiple sources; As of January 2025 this platform included 115 studies ([ImmPort Search](https://gcc02.safelinks.protection.outlook.com/?url=https%3A%2F%2Fimmport.org%2Fshared%2Fsearch%3Ftext%3DCIVICs&data=05%7C02%7Cjennifer.gordon2%40nih.gov%7Ca40ffc9629f046d872c008dc17c15ba7%7C14b77578977342d58507251ca2dc2b06%7C0%7C0%7C638411366431610975%7CUnknown%7CTWFpbGZsb3d8eyJWIjoiMC4wLjAwMDAiLCJQIjoiV2luMzIiLCJBTiI6Ik1haWwiLCJXVCI6Mn0%3D%7C3000%7C%7C%7C&sdata=d5IchnLJGgTeT7bjYwiIMvRVlPxnI3lfKXr4G1ZLP1g%3D&reserved=0)). Recent developments in the portal include:   - A new interactive data submission application with live validation and error resolution capabilities to enable optimal sharing of data internally and to public databases - New standards to capture data produced in vaccine and cohort studies and maximize the dissemination of results - A new data search application which allows scientists with [iDPCC Portal](https://www.ceirr-network.org/centers/idpcc) access to view and export data - An improved Reagent page interface to allow influenza researchers to obtain specific reagents for vaccine studies |
| **Milestone 6.3.d***:* *Conduct mapping of intellectual property for improved influenza vaccines to identify synergies in approaches that may be used to develop new partnerships.*  IN PROGRESS   - WHO and LSHTM are developing the FVIVA, which will include mapping of intellectual property for improved influenza vaccines. |
| **Milestone 6.3.e**: *Taking into consideration ongoing global negotiations related to intellectual property (e.g., the Pandemic Agreement), develop a consensus vision for sharing intellectual property or proprietary technologies related to improved influenza vaccines that includes benefit sharing and equitable access for LMICs.*  NO PROGRESS |
| **Milestone 6.3.f**: *Identify a party to convene a group of key R&D stakeholders tasked with developing and establishing a set of harmonized data standards and explore mechanisms for broader data sharing.*  NO PROGRESS |
| ***Strategic Goal 6.4:*** Address regulatory challenges in the evaluation and licensure of next-generation, broadly protective, and universal influenza vaccines. |
| *High-Priority Milestone*  **Milestone 6.4.a***:* *Convene a workshop to address regulatory science issues regarding the evaluation of next-generation and broadly protective influenza vaccines for pandemic preparedness and seasonal influenza vaccination and publish a consensus summary report.*  IN PROGRESS   - WHO and LSHTM are engaged in the FVIVA project, which includes conducting an introductory workshop on regulatory considerations for next-generation influenza vaccines. - [Cavaleri 2025](https://pubmed.ncbi.nlm.nih.gov/40155114/) summarized a series of proposed actions for improving efficiencies in ethical and regulatory review of global clinical research needed for rapid responses to future epidemics or pandemics. The actions include leveraging existing clinical trial networks and capacity-building initiatives, advancing joint and parallel regulatory and ethics reviews, improving transparency in approval requirements, and facilitating export/import of investigational products for clinical trials. - EMA and FDA regulators participated in discussions regarding influenza vaccine R&D at the ISIRV *Correlates of Protection for Next Generation Influenza Vaccines* conference in Seattle, March 1-3, 2023. - [GloPID-R 2023](https://www.glopid-r.org/wp-content/uploads/2023/05/glopid-r-funders-living-roadmap-for-clinical-trial-coordination.pdf): the Global Research Collaboration for Infectious Disease Preparedness (GloPID-R) developed a guide for funding agencies to improve the coordination of clinical trials in response to infectious disease outbreaks, in part to help avoid delays in initiating clinical trials of novel vaccines such as the 2009 influenza A(H1N1) vaccine. - [Baylor 2022](https://pubmed.ncbi.nlm.nih.gov/36560546/) reviewed regulatory pathways and processes relevant to pandemic influenza, including how they can be strengthened and globally coordinated, and identified opportunities to provide better approaches, tools, and methods to accelerate and improve vaccine development and evaluation. - [Pecetta 2022](https://pubmed.ncbi.nlm.nih.gov/35353544/) summarized economic and regulatory lessons learned from the COVID-19 pandemic. |
| **Milestone 6.4.b**: *Identify a framework to address post-marketing assessment of safety and relative effectiveness of broadly protective or universal influenza vaccines compared with conventional seasonal influenza vaccines.*  IN PROGRESS   - [Baylor 2022](https://pubmed.ncbi.nlm.nih.gov/36560546/) identified opportunities for improving the monitoring of safety and effectiveness of new influenza vaccines in the United States. - [Diez-Domingo 2022](https://europepmc.org/article/MED/36261918) assessed the role of public-private partnerships in post-licensure monitoring of vaccine safety and efficacy, based on lessons learned from the European DRIVE (Development of Robust and Innovative Vaccine Effectiveness) project. - [Levison 2022](https://pubmed.ncbi.nlm.nih.gov/35913431/) conducted a retrospective nationwide population-based case–control study in Denmark to estimate the magnitude and duration of GBS risk following influenza vaccination, as an example of assessing post-marketing safety. - [Salmon 2021](https://pubmed.ncbi.nlm.nih.gov/34011502/) used vaccine safety case studies to illustrate potential safety issues with new vaccines (including pandemic influenza vaccine) and identify key lessons for evaluating the safety of new vaccines via post-marketing surveillance. - [Zuber 2021](https://pubmed.ncbi.nlm.nih.gov/34011500/) recognized that novel vaccine technologies may have different safety profiles requiring adapted pharmacovigilance approaches and highlights the role of the Global Advisory Committee on Vaccine Safety in post-licensure surveillance. |
| **Milestone 6.4.c**: *Develop consensus on best practices for using CHIVIM studies to support licensure of universal or broadly protective influenza vaccines.*  IN PROGRESS   - [Meln 2025](https://pubmed.ncbi.nlm.nih.gov/39824043/) summarized outcomes from an Inno4Vac workshop held on March 20, 2024, to address the standardisation of clinical procedures, ethical considerations, endpoints, and data integrity in CHIM studies, including influenza-related studies. - [Cavaleri 2024](https://pubmed.ncbi.nlm.nih.gov/38341355/) summarized discussions at the 4th Controlled Human Infection Model (CHIM) meeting on regulatory issues in CHIM studies, held on May 24, 2023; topics includedGMP production of challenge agents, ethics, community engagement, pre-existing immunity, and clinical, immunologic, and microbiologic endpoints. - [Wellcome Trust 2023](https://wellcome.figshare.com/articles/online_resource/Use_of_Human_Infection_Studies_in_Vaccine_Development/21997619/1) identified how human infection study data can inform regulatory approval of new vaccines (e.g., by providing insights into disease mechanisms, host responses to infection, correlates of protection, optimal doses for phase 3 trials, and down-selecting vaccine candidates for clinical trials.) |
| **Milestone 6.4.d**: *Develop consensus definitions on clinical endpoints for severe influenza disease in vaccine efficacy studies.*  IN PROGRESS   - [Braunfeld 2022](https://pubmed.ncbi.nlm.nih.gov/35717265/) found that among pediatric influenza vaccine efficacy trials, primary outcome measures and clinical specimen collection criteria were highly variable; policy and implementation decisions based on VE data are limited, given the absence of influenza vaccination programs in most LMICs. |
| **Milestone 6.4.e***:* *Develop and validate a standard scale for assessing influenza disease severity in clinical studies.*  PARTIALLY ACCOMPLISHED   - [Chow 2021](https://pubmed.ncbi.nlm.nih.gov/34673782/) (US CDC) developed a quantitative scale in adults hospitalized with influenza-associated lower respiratory tract infection demonstrating a broad distribution of physiologic severity; efforts are needed to further validate this scale to fully meet the milestone. |
| **Milestone 6.4.f**: *Develop and validate correlates of protection for severe influenza disease as a surrogate endpoint in clinical studies.*  NO PROGRESS |
| **Milestone 6.4.g**: *Develop a consensus approach on clinical methodologies for demonstrating vaccine effectiveness in preventing severe influenza disease in different geographical settings.*  NO PROGRESS |

| **Abbreviations:**  Ab: antibody; ABS: Access and benefit sharing; Ad5: Adenovirus-5; ADCC: Antibody-dependent cellular cytotoxicity; ADCP: Antibody-dependent cellular phagocytosis; BARDA: Biomedical Advanced Research and Development Authority (HHS); BMI: body-mass index; bnAb: broadly neutralizing antibody; ccIIV4: Cell-culture inactivated influenza virus, quadrivalent; CDC: Centers for Disease Control and Prevention (HHS); CEIRR: Centers of Excellence for Influenza Research and Response (NIAID); CEPI: Coalition for Epidemic Preparedness Innovations; cGAMP: Cyclic guanosine monophosphate–adenosine monophosphate; cHA: chimeric hemagglutinin; CHIVIM: Controlled human influenza virus infection model; CIDRAP: Center for Infectious Disease Research and Policy (University of Minnesota); CIVICs: Collaborative Influenza Vaccine Innovation Centers (NIAID); CMC: Chemistry, manufacturing, and controls; COVID-19: Coronavirus disease 2019; DIVINCI: Dissection of Influenza Vaccination and Infection for Childhood Immunity (consortium); DRIVE: Dynamics of the immune responses to repeat influenza vaccination exposures study; ELISA: Enzyme-linked immunoassay; ELISpot: Enzyme-Linked ImmunoSpot; ELLA: Enzyme-linked lectin assay; ELLA-NI: enzyme-linked lectin assay-neuraminidase inhibition; EMA: European Medicines Agency; ESWI: European Scientific Working Group on Influenza; FAO: Food & Agriculture Organization of the United Nations; F_c_: Fragment crystallizable; FcγR: Fragment crystallizable gamma receptor; FDA: US Food and Drug Administration; FVIVA: Full value of influenza vaccine assessment; FVVA: Full value of vaccine assessment; GBS: Guillain–Barré syndrome; GC: Germinal center; GIHSN: Global Influenza Hospital Surveillance Network; GISRS: Global Influenza Surveillance and Response System; HA: Hemagglutinin; HA1: Hemagglutinin 1; HAI: Hemagglutinin inhibition; HCW: Health-care worker; HHS: US Department of Health and Human Services; HI: Hemagglutinin inhibition; HLA: Human leukocyte antigen; HPAI: Highly pathogenic avian influenza; HSCT: hematopoietic stem cell transplant; IAV: Influenza A virus; IBV: Influenza B virus; ICS: Intracellular cytokine staining; ID: Intradermal; IFN-ɣ: Interferon-ɣ; IFPMA: International Federation of Pharmaceutical Manufacturers and Associations; IgA: Immunoglobulin A; IgG: Immunoglobulin G; IgM: Immunoglobulin M; IIV: Inactivated influenza virus vaccine; ILI: influenza-like illness; IM: Intramuscular; IMPRINT: Immunological memory to prior influenza over time; IN: Intranasal; IPG: Institute for Pathogen Genomics; ISIRV: International Society for Influenza and other Respiratory Virus Diseases; IVR: Influenza Vaccines Research & Development Roadmap; LAIV: Live-attenuated influenza vaccine; LMICs: Low- and middle-income countries; LSHTM: London School of Hygiene & Tropical Medicine; M1: matrix protein 1; mAb: monoclonal antibody; MAS-1: Macrophage activation syndrome-1; MDCK: Madin-Darby canine kidney cells; ME&A: Monitoring, evaluation, and adjustment; MN: Microneutralization; MPP: Measles virus phosphoprotein; mRNA: Messenger ribonucleic acid; mRNA-LNP: Messenger ribonucleic acid lipid nanoparticle; MSD: Meso Scale Discovery platform; N1: Neuraminidase-1; N2: Neuraminidase-2; NA: Neuraminidase; nAb: Neutralizing antibody; NAI: Neuraminidase inhibition; NALT: Nasal-associated lymphoid tissue; NASEM: National Academies of Sciences, Engineering, and Medicine; NGIV: Next-generation influenza vaccine; NHP: Nonhuman primate; NIAID: US National Institute of Allergy and Infectious Diseases; NIH: National Institutes of Health; NK: Natural killer T-cells; NP: Nucleoprotein; NYU: New York University; OFFLU: Global Network of Expertise on Animal Influenza; PAHO: Pan American Health Organization; PIVI: Partnership for International Vaccine Initiatives (Task Force for Global Health); PPCs: Preferred product characteristics; QIV: Quadrivalent inactivated influenza vaccine; R&D: Research and development; RBC: Red blood cell; RCT: Randomized clinical trial; rHA: Recombinant hemagglutinin; RIV4: Recombinant influenza virus vaccine, quadrivalent; rNA: Recombinant neuraminidase; RNA: Ribonucleic acid; RSV: Respiratory syncytial virus; rVE: Relative vaccine effectiveness; SARS-CoV-2: Severe acute respiratory syndrome coronavirus 2; SRID: Single-radial immunodiffusion assay; SOP: Standard operating procedure; TCR: T-cell receptor; TFH: T-follicular helper cells; TIV: Trivalent inactivated influenza vaccine; T_RM_: Resident memory T cells; UK: United Kingdom; US: United States; VE: Vaccine efficacy/effectiveness; VLP: Virus-like particle; WHO: World Health Organization. |
| --- |
